# An operational spatiotemporal prediction framework for sector congestion in air traffic management

**DOI:** 10.1371/journal.pone.0355927

**Published:** 2026-10-27

**Authors:** Huaqun Chen, Zhenyu Gao, Xiaoyu Yan, Yuehui Yi, Jintao Gong, Lin Wu

**Affiliations:** 1 Air Traffic Management Institute, Civil Aviation Flight University of China, Guanghan, Sichuan, China; 2 Operation Management Center, Civil Aviation Administration of China Air Traffic Management Bureau, Beijing, China; National University of Defense Technology, CHINA

## Abstract

En-route sector congestion poses a critical challenge to air traffic safety, operational efficiency, and resource-efficient airspace operation in high-density airspace. Because sector operations are spatially networked, locally emerging congestion may propagate through adjacent or upstream grid areas, leading to large-scale delays, increased fuel consumption, and elevated controller workload. This study proposes an operational spatiotemporal prediction framework for 15-minute-ahead sector congestion warning. First, processed grid-level operational states are converted into three congestion levels using K-means++ clustering based on six direct observable operational-state indicators. Second, a hybrid GNN-BiLSTM-Cross-Attention model predicts future congestion levels and local T2 hotspots from historical 5-minute grid-window sequences, without using future observed features. Third, SHAP/SHEP-style post-hoc grouped attribution is applied after inference to distinguish external triggering risk from congestion-propagation risk and to decompose external contributions from weather, restricted areas, controller workload, and wake intensity. A case study using 2023 processed grid-level operational data from the ZGGGAR15 high-altitude en-route sector achieves TEST@VAL Weighted F1 = 0.9773, L2-F1 = 0.9602, Precision = 0.9545, Recall = 0.9660, and AP = 0.9767 for rare high-congestion warning.The results indicate that the proposed framework provides accurate and spatially interpretable predictions with potential decision-support value for proactive air traffic flow management.

## 1. Introduction

En-route sector congestion has become an increasingly critical challenge in modern air traffic management, particularly under high-density and high-altitude en-route operations. As traffic demand approaches or exceeds the dynamic capacity of an airspace sector, operational efficiency degrades, controller workload intensifies, and flight trajectories are frequently adjusted through speed control, rerouting, or holding. These local disturbances do not remain isolated. Due to the structured topology of en-routes and the strong spatiotemporal coupling of aircraft movements, congestion can propagate across adjacent and upstream airspace, leading to large-scale delays, additional fuel consumption, and elevated flow-management pressure. Accurate and timely prediction of en-route sector congestion is therefore essential for ensuring the safety and efficiency of airspace operations, and for providing indirect support for future resource-efficiency and environmental assessment.

Unlike surface or terminal-area congestion, en-route sector congestion arises within a highly constrained aerospace system governed by strict flight rules, vertical stratification, wake turbulence separation standards, and air traffic control (ATC) procedures. Sector capacity is not a fixed quantity but a dynamic result of interactions among traffic demand, airspace structure, meteorological conditions, and human-machine control processes. In current operations, congestion assessment largely relies on air traffic controllers’ real-time judgment and experience, which, although effective in tactical control, lacks standardized quantitative criteria and predictive capability. This limitation restricts the ability of air traffic flow management (ATFM) systems to provide proactive congestion warnings and refined control strategies. In alignment with modern aviation initiatives such as Flight and Flow Information for a Collaborative Environment (FF-ICE), which emphasize data sharing, collaborative decision-making, and trajectory-based operations, there is a growing need for predictive models that support tactical control, identify spatial congestion risk in advance, and provide an operational basis for subsequent efficiency and environmental assessment. Shared flight intent and planned trajectory information can provide prior information for traffic demand, route pressure, and longer-horizon congestion prediction, but it is complementary to, rather than a substitute for, prediction from actual operating states.

Existing research on air traffic congestion prediction has primarily focused on airport surface operations and terminal areas, where traffic patterns are relatively localized and infrastructure constraints are more explicit. A variety of statistical and machine learning methods, including regression models, recurrent neural networks, and hybrid optimization frameworks, have been applied to predict delays and congestion levels in these environments. While these approaches have demonstrated promising performance, they cannot be directly extended to en-route sectors. En-route congestion exhibits distinct characteristics, such as network-wide propagation, strong spatial dependency along en-routes, and sensitivity to altitude structure and control interventions, which are not adequately captured by models designed for terminal-area scenarios.

At the sector level, several congestion indicators have been proposed, including aircraft count, traffic density, and controller workload. More recently, data-driven methods have been introduced to classify sector states and forecast congestion evolution. However, most existing models either rely on simplified airspace representations or treat spatial and temporal dependencies separately, limiting their ability to capture the coupled dynamics of congestion propagation. Moreover, many studies assume flexible airspace configurations and data environments that differ substantially from the rigid sectorization and complex civil–military coordination present in China’s airspace system, reducing their operational applicability.

To address these challenges, this study proposes a data-driven framework for fine-grained, 15-minute-ahead en-route sector congestion prediction that explicitly models the spatiotemporal dynamics of airspace operations. First, a congestion identification indicator system is constructed from the perspectives of traffic flow dynamics, control interventions, environmental constraints, and management pressure. Within this system, six direct operational-state variables are clustered into three congestion levels using the K-means++ algorithm, while external and management-related variables are retained as visible predictors and attribution factors rather than clustering inputs. Second, a hybrid deep learning architecture integrating Graph Neural Networks (GNN), Bidirectional Long Short-Term Memory networks (BiLSTM), and cross-attention is developed. The GNN captures spatial correlations and congestion propagation along the en-route network, the BiLSTM models temporal dependencies and inertia in traffic evolution, and the cross-attention module adaptively fuses spatial and temporal representations to enhance predictive capability. Finally, the proposed framework predicts future congestion levels and local T2 hotspot probabilities from historical 5-minute grid-window sequences without using future observed features, and then applies SHAP/SHEP-style post-hoc grouped attribution to summarize whether a warning is mainly related to external triggering factors or congestion propagation.

The main contributions of this study can be summarized as follows:

1. A sector-oriented congestion identification framework is established. It transforms observable grid-level operating states into objective three-level congestion labels through K-means++ clustering on direct operational-state indicators, while preserving external factors for prediction and interpretation.
2. A hybrid GNN-BiLSTM-Cross-Attention model is proposed to jointly model airspace network topology, temporal traffic evolution, and multi-factor interactions, addressing the spatiotemporal coupling inherent in en-route congestion propagation.
3. A prediction-and-explanation strategy is developed to report 15-minute-ahead congestion levels, local T2 hotspot probabilities, and post-hoc warning-source attribution. The attribution analysis is generated from the trained model and visible input features after inference, rather than being used as an additional supervised target in the training data.
4. A case study based on a representative busy sector demonstrates that the proposed method achieves high rare-congestion warning accuracy and practical inference efficiency, indicating its potential to support proactive congestion mitigation. Possible fuel-burn and carbon-emission benefits are discussed only as indirect operational implications that require dedicated environmental validation in future work.

## 2. Related work

Air traffic congestion prediction has been extensively studied in air traffic management, with research efforts spanning airport surface operations, terminal-area control, and en-route airspace. To clarify the positioning of the present study, existing work is reviewed from four perspectives: terminal-area and airport congestion prediction, en-route sector congestion identification and modeling, spatiotemporal learning for air traffic systems, and cross-domain intelligent-transportation studies that provide methodological or conceptual references.

### 2.1. Terminal-area and airport congestion prediction

A substantial body of research focuses on congestion and delay prediction in airport surface and terminal-area operations, where traffic patterns are relatively localized and infrastructure constraints are explicit. Statistical models and machine learning approaches have been widely applied to predict departure queues, surface congestion, and arrival delays. For example, Xu et al. employed a genetic algorithm–optimized LSTM model to predict congestion levels at large airports, demonstrating improved temporal prediction accuracy [1]. Wang et al. developed hybrid regression and machine learning models to estimate departure flow congestion under varying operational conditions [2]. Yin et al. proposed deep learning frameworks to uncover hidden dynamics in airport traffic networks and improve delay prediction performance [3]. Related studies have further explored multimodal deep learning and physics-informed learning architectures for terminal-area traffic flow prediction [4,5].

Although these studies have achieved promising results in airport and terminal environments, terminal-area congestion is fundamentally different from en-route sector congestion. Terminal operations are dominated by runway capacity, ground infrastructure, and localized sequencing constraints, whereas en-route congestion emerges from network-wide interactions among multiple sectors, altitude layers, and air traffic control strategies. Consequently, models designed for terminal-area scenarios cannot be directly extended to en-route airspace without explicitly addressing spatial propagation and sector-level coupling effects.

### 2.2. En-route sector congestion identification and modeling

At the en-route level, congestion has been recognized as a multi-factor phenomenon driven by imbalances between traffic demand and sector capacity, influenced by weather conditions, airspace restrictions, controller workload, and wake turbulence separation requirements [6–9]. Several congestion indicators have been proposed, including aircraft count, traffic density, occupancy rate, and controller workload metrics [10,11]. Adacher et al. investigated cooperative sector management strategies for mitigating congestion through coordinated control [6], while Balakrishnan analyzed congestion from a control and optimization perspective within air transportation systems [12]. Probabilistic congestion modeling and complexity-based indicators have also been introduced to quantify sector congestion risk under uncertainty [7,8].

More recently, data-driven approaches have been adopted to classify sector states and predict congestion evolution [13]. Fuzzy clustering and probabilistic methods have been applied to sector congestion identification, and machine learning models have been explored to forecast congestion probabilities under capacity uncertainty [14–16]. However, many of these studies rely on coarse sector-level representations and static or weakly dynamic features. The spatial structure of en-routes and the propagation of congestion across neighboring sectors are often simplified or ignored, limiting the ability of these models to capture fine-grained congestion dynamics [11,17].

In addition, most existing sector-level studies assume flexible airspace configurations and data environments that differ substantially from the rigid sectorization and complex civil–military coordination present in China’s airspace system. As a result, their applicability to high-density, high-altitude en-route operations under strict regulatory constraints remains limited [9,18]. Studies conducted in the United States and Europe have examined sector capacity, trajectory-based operations, flow prediction, and network-level demand management in U.S. and European ATM contexts. These studies provide useful methodological references, but their data interfaces, sector configurations, and coordination mechanisms differ from China’s airspace system; their conclusions therefore require adaptation before being applied to the present setting [9,18].

### 2.3. Spatiotemporal learning for air traffic systems

With the increasing availability of high-resolution trajectory and operational data, spatiotemporal learning methods have attracted growing attention in air traffic research. Recurrent neural networks, such as LSTM and GRU, have been widely used to model temporal dependencies in traffic flow and delay propagation [1,19]. Graph-based models have been introduced to represent air traffic networks, enabling the modeling of spatial dependencies among airports, sectors, or route segments [11,20]. More advanced architectures, including graph convolutional networks and graph attention networks, have been applied to capture network-wide interactions and congestion propagation effects [5,17].

Recent studies have further explored hybrid spatiotemporal models that combine graph neural networks with recurrent architectures or convolutional neural networks [20,21]. Representative transportation forecasting architectures include ST-GCN, ASTGCN, DCRNN, and Graph WaveNet, which use graph convolution, attention, diffusion recurrence, or adaptive graph learning to model networked traffic dynamics [22–26]. Event-oriented models such as STGNPP and STENPP further show that sparse congestion events and traffic-flow tipping points can be treated as spatiotemporal event prediction problems rather than only dense traffic-state regression tasks [27,28]. Transformer and Informer-like attention mechanisms have also been introduced to enhance long-range dependency modeling and adaptive feature fusion in traffic flow prediction tasks [19,29]. These approaches have demonstrated strong performance in traffic flow and delay forecasting.

Cross-domain intelligent transportation and perception studies also provide useful references for shared representation learning, graph-based forecasting, uncertainty-aware warning, and resource-aware deployment [30]. Driver-state perception studies using dual-pathway multi-task learning show how heterogeneous traffic-related observations can be processed through shared and task-specific representations when several correlated outputs are required. Similarly, the M-GBT multi-region load forecasting framework illustrates how graph-based regional coupling and Transformer-style temporal modeling can be combined for multi-node short-term prediction under spatial dependence. These studies are not direct baselines for en-route sector congestion, but they provide methodological support for multi-output warning, graph-temporal fusion, and deployable modeling in structured operational systems [31,32].

Traffic perception studies such as vehicle-mounted traffic sign detection, spike-driven object detection, and metaverse-assisted ITS visual perception further broaden the conceptual framing of this work. Their main relevance lies not in transferring visual detection architectures to ATM, but in the shift from isolated prediction toward real-time traffic-state perception, multi-source scene understanding, robust recognition under complex conditions, and deployable decision-support systems. From this perspective, sector congestion prediction can be interpreted as a structured-airspace traffic perception problem: the model infers future operating states from grid-level observations, identifies spatial risk hotspots, and provides post-hoc warning explanations for operational decision support [33–35].

Nevertheless, most existing spatiotemporal models focus on dense traffic-flow or delay forecasting and do not explicitly address the joint operational need to predict congestion levels, local severe-congestion hotspots, and interpretable warning evidence under multi-factor sector conditions. Direct transfer from road-network models to en-route airspace is also limited because sector congestion is constrained by airway geometry, altitude-layer operation, weather-affected capacity, restricted areas, wake separation, controller workload, and tactical control interventions. Moreover, the interaction between spatial propagation and temporal evolution is often handled through simple feature concatenation or sequential stacking, which may be insufficient to capture the complex dynamics of congestion formation, propagation, and mitigation in en-route airspace.

### 2.4. Motivation and contribution of this study

In summary, although significant progress has been made in air traffic congestion prediction, several gaps remain in the context of en-route sector operations. Existing methods either focus on terminal-area scenarios, rely on coarse sector-level representations, or do not sufficiently couple spatial propagation with short-term temporal evolution at the grid level. In addition, limited attention has been paid to explaining predicted warnings in a way that distinguishes external triggering factors from congestion-propagation evidence, which is important for tactical air traffic flow management [14–16].

To bridge these gaps, this study proposes a sector-oriented spatiotemporal congestion prediction framework that integrates K-means++ congestion labeling with a hybrid GNN-BiLSTM-Cross-Attention architecture and SHAP/SHEP-style post-hoc grouped attribution. Unlike end-to-end road traffic models, each module is aligned with an ATM mechanism: graph modeling represents route-related spatial propagation, bidirectional recurrent modeling captures short-term congestion evolution, cross-attention fuses spatial and temporal representations, and post-hoc attribution summarizes warning evidence into external triggering and congestion-propagation groups. By jointly modeling airspace network topology, temporal traffic dynamics, and visible operational factors, the proposed approach aims to provide accurate, fine-grained, and interpretable predictions for en-route sector congestion under realistic operational constraints.

## 3. En-route sector congestion identification and evolution framework

This section formalizes the identification and evolution mechanism of en-route sector congestion from a human-machine-environment-management system perspective. It first describes the sector structure and congestion evolution mechanism, then defines the observable grid-level indicators used for K-means++ labeling, prediction modeling, and post-hoc attribution.

### 3.1. Sector operational structure

The en-route sector is the core control unit in air traffic management, defined as an irregular polygonal area within specific airspace boundaries. It serves as a key link between departure and arrival terminal areas. Traffic flows follow a departure terminal → multi-sector relay → arrival terminal logic, forming continuous, cross-regional air traffic flows. Altitude assignments comply with CAAC standards, RVSM vertical separation, and “odd/even east/west” route directions, creating a structured vertical layer system. To clarify the operational structure, spatial boundaries, and vertical organization of an en-route sector, Fig 1 illustrates the sector geometry, altitude layers, and traffic flow directions considered in this study.

**Fig 1:**
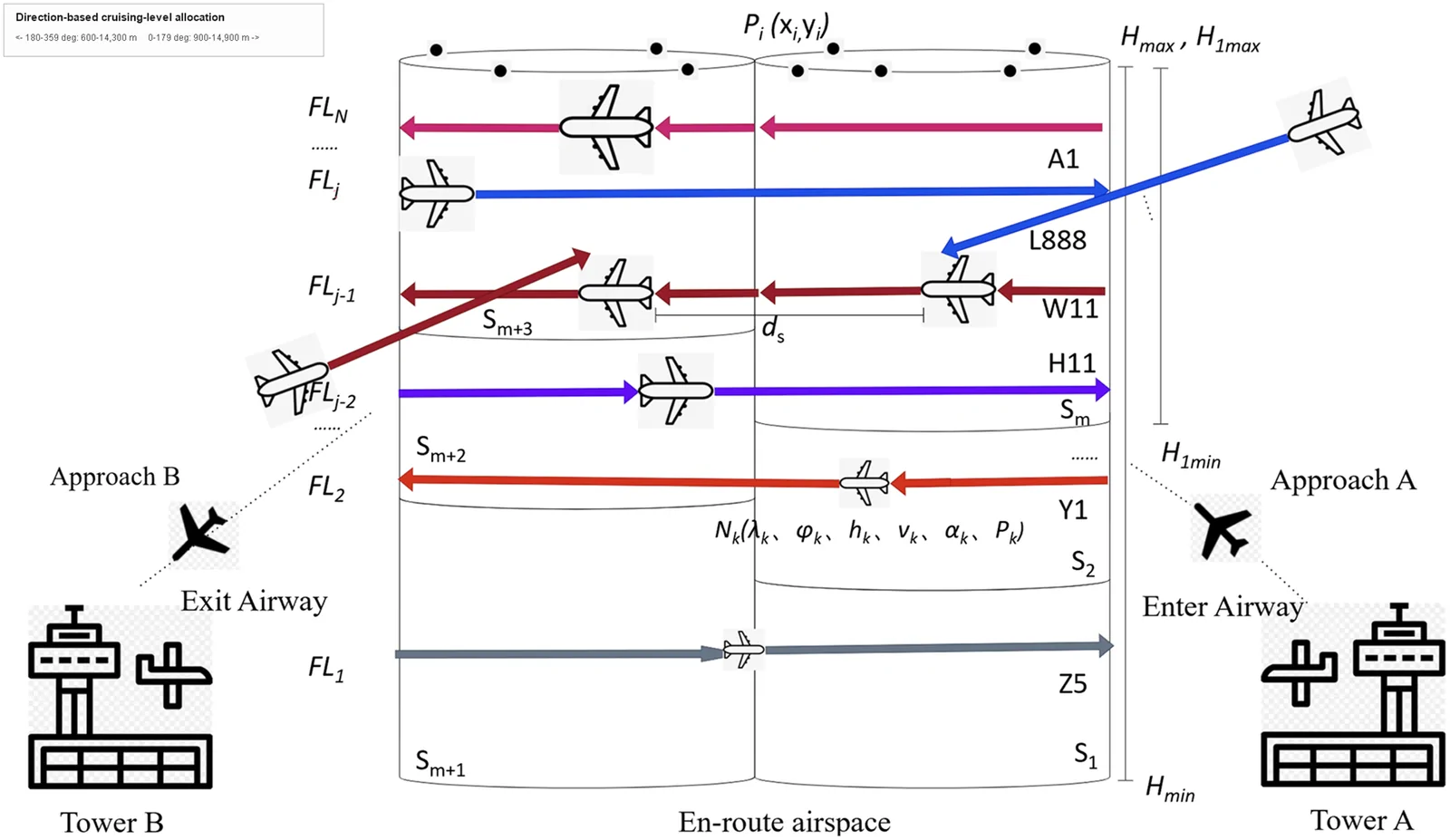
Illustration of en-route sector structure and operations.

The sector is defined by its horizontal boundaries ($P_{i}(X_{i},Y_{i})$) and vertical limits between $H_{\text{min}}$ and $H_{\text{max}}$, containing multiple flight levels ($FL_{\text{1}},\ldots ,FL_{N}$). Airways (e.g., $A_{\text{1}},L_{\text{888}}$) guide traffic flow, connecting neighboring sectors ($S_{\text{1}},S_{\text{2}},\ldots$). Aircraft within the sector are fully described by state vectors $N_{k}$ encompassing position (λ,ϕ,h), velocity (v,α), and type (P), operating under standard horizontal separation $d_{s}$.

### 3.2. Congestion evolution and management

En-route sector congestion is defined as a dynamic operational state in which the instantaneous traffic demand exceeds the effective sector capacity under the combined influence of airspace structure, controller workload, environmental disturbances, and traffic management constraints. Rather than representing a complete traffic standstill, congestion corresponds to a degraded operational condition characterized by reduced throughput efficiency, intensified control interventions, and elevated controller work-load. From an operational perspective, this imbalance manifests through prolonged aircraft dwell times, increased frequency of speed, heading, or altitude-related control adjustments, and a higher likelihood of holding instructions. Due to the continuous movement of aircraft and the networked structure of en-route airspace, locally emerging congestion can propagate across neighboring areas through traffic interactions, forming a spatiotemporally evolving congestion process at the sector level.

To illustrate the defined congestion formation and propagation mechanism under external disturbances and control actions, Fig 2 presents the conceptual evolution of sector congestion together with representative air traffic control measures.

**Fig 2:**
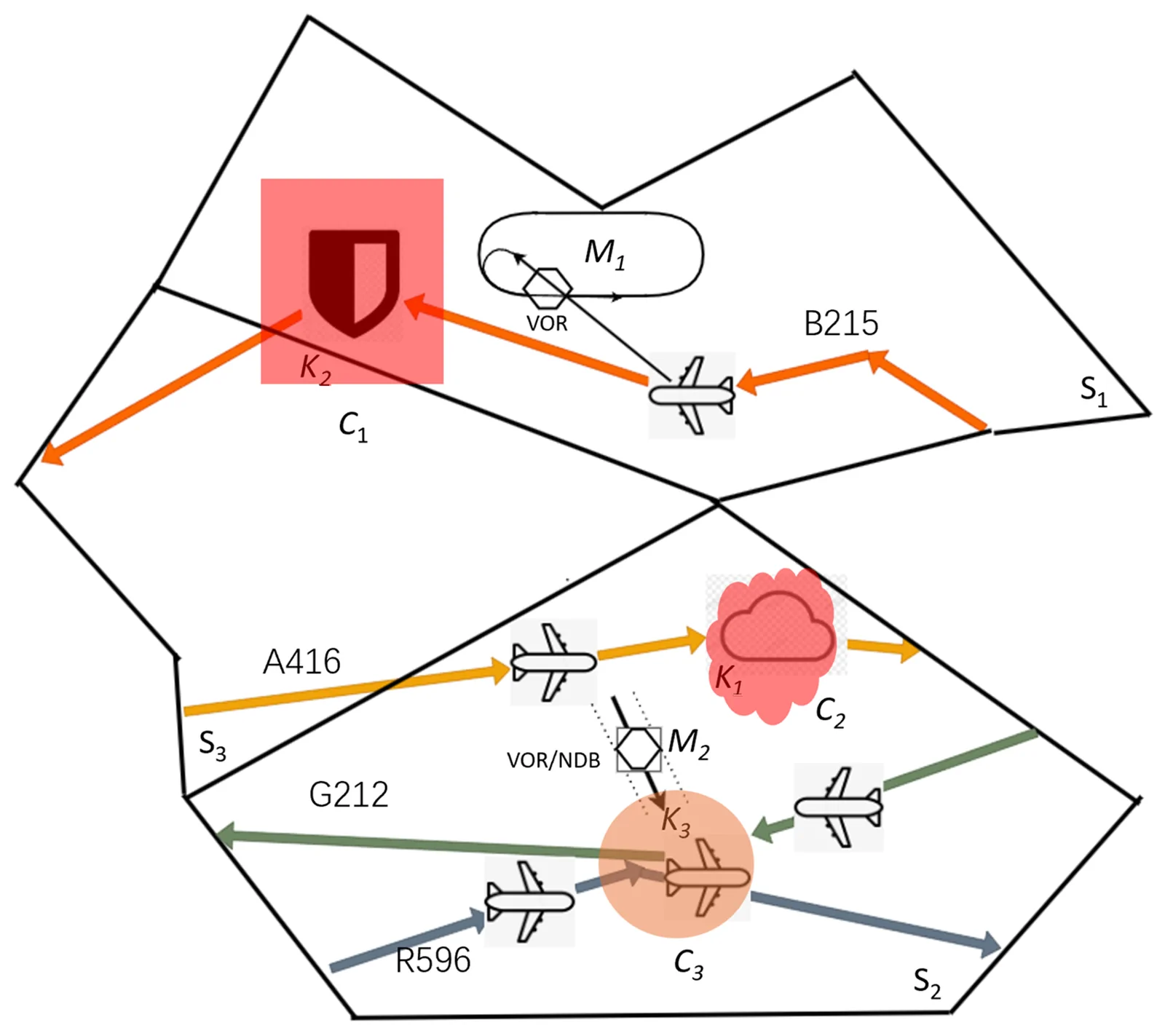
Congestion evolution and air traffic control measures.

Although the sector airspace exhibits an inherently three-dimensional structure, the proposed modeling framework focuses on horizontal spatial interactions in the latitude-longitude plane. This simplification is motivated by the fact that en-route traffic within the studied sector is organized into a limited number of discrete cruising altitude layers, and sector congestion formation is often dominated by horizontal traffic accumulation and route interactions. Nevertheless, vertical operations remain operationally important. Altitude conflicts, climb/descent interactions, flight-level imbalance, and tactical altitude changes can increase controller workload and reduce effective capacity. Therefore, the present model does not treat altitude as a full spatial dimension, but incorporates vertical complexity through altitude-change frequency and related control-intervention indicators. A more complete 3D graph representation that explicitly models altitude layers is left for future multi-sector work.

The figure illustrates the formation of congestion areas ($C_{\text{1}},C_{\text{2}},C_{\text{3}}$) under the influence of key factors: meteorological impacts ($K_{\text{1}}$), restricted airspace ($K_{\text{2}}$), and congestion-induced flow surges ($K_{\text{3}}$). Congestion severity is color-coded (red: severe; orange: moderate). In response, standard control measures such as holding patterns ($M_{\text{1}}$) and rerouting ($M_{\text{2}}$) are deployed to alleviate congestion and manage traffic flow.

Congestion evolution is a dynamic process driven by multiple factors with significant spatiotemporal propagation. Weather, restricted areas, wake-related separation pressure, and management workload may reduce effective capacity or increase operating constraints. When traffic demand persistently exceeds this dynamic threshold, local congestion emerges and can propagate to adjacent or upstream grid areas through flight-flow interactions and tactical control responses. Therefore, external factors are treated as triggering or capacity-affecting conditions, while the congestion state itself is identified from directly observable traffic and control-intervention indicators.

### 3.3. Congestion state representation

To construct a quantitative congestion identification and prediction model, abstract imbalance states must be translated into measurable features. Based on the human–machine–environment–management system perspective, the conceptual representation of congestion-related factors is summarized in Fig 3.

**Fig 3:**
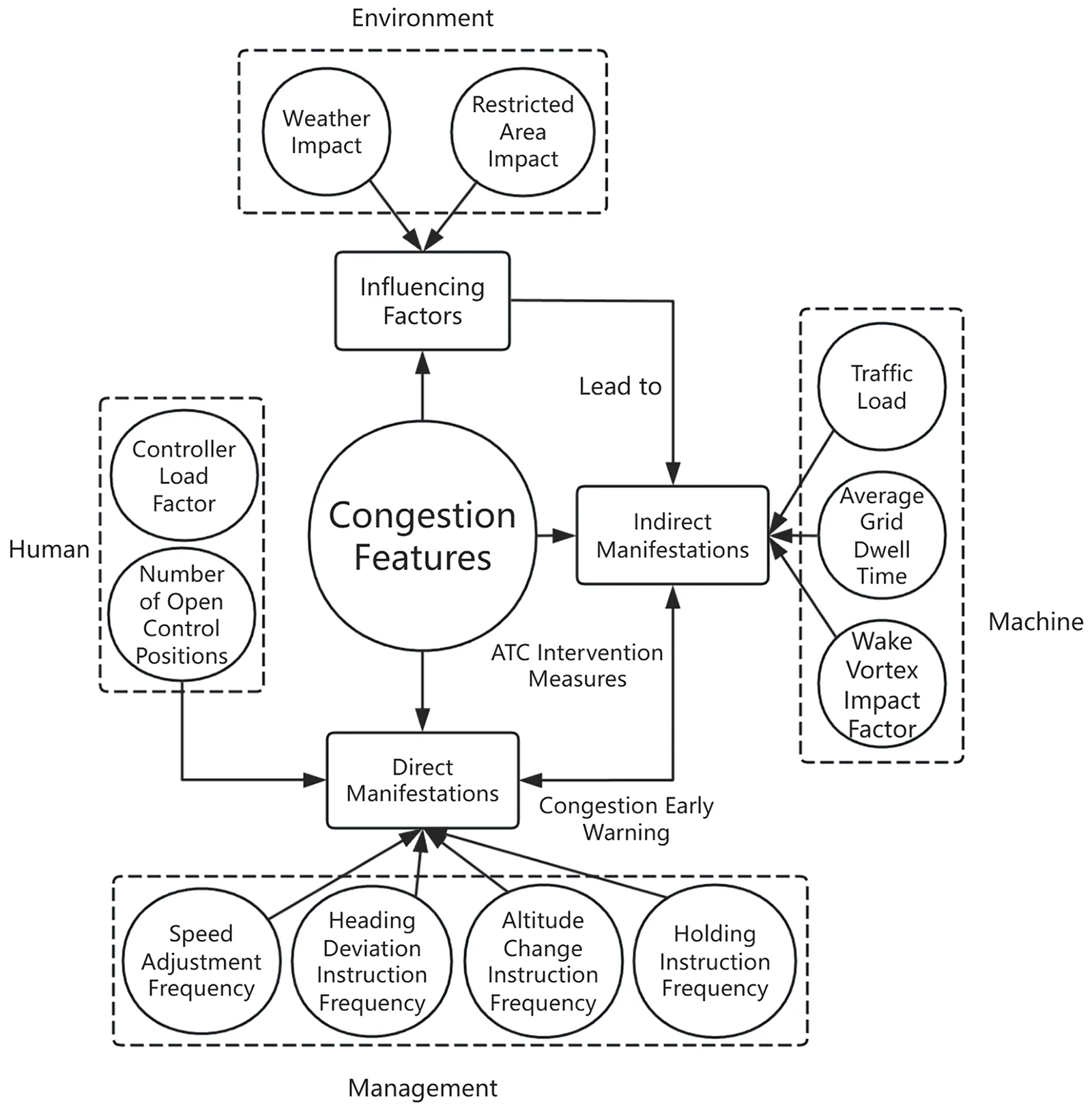
Conceptual representation of en-route sector congestion.

Controller Workload Features: controller load reflects management pressure and operational complexity. It is retained as a visible prediction and attribution factor, but it is not used as a direct K-means++ clustering variable.

Traffic Flow Dynamics: congestion manifests as deviations from smooth and efficient flow, represented by traffic load and prolonged dwell time. These variables are direct operational-state indicators for K-means++ labeling.

Environmental Constraints: Weather and temporary airspace restrictions reduce available capacity, imposing rigid or flexible constraints that exacerbate imbalance.

System Resources & Control: tactical measures to restore balance, such as speed adjustment, heading deviation, altitude change, and holding instructions, reflect active system interventions and are used as direct operational-state indicators for clustering.

Based on these dimensions, the following parameter set is defined for congestion identification, prediction, and attribution. The six direct operational-state indicators, namely traffic load, average dwell time, speed adjustment frequency, heading deviation frequency, altitude change frequency, and holding instruction frequency, are used for K-means++ congestion labeling. Weather intensity, restricted-area impact, controller workload, and wake intensity are retained as visible prediction inputs and post-hoc attribution factors. The resulting indicator system is summarized in Table 1.

**Table 1:** Parameter definitions for the final 15-column grid-window record.

Parameter	Symbol	Category	Definition
**Time step**	t	Temporal index	5-minute grid-window identifier used for temporal ordering and sample construction.
**Grid row**	rᵢ	Spatial index	Row index of grid i in the sector grid system.
**Grid column**	cᵢ	Spatial index	Column index of grid i in the sector grid system.
**Traffic load**	Nᵢ(t)	Operational-state indicator	Aircraft count or occupancy pressure of grid i during time window t.
**Average dwell time**	D̄ᵢ(t)	Operational-state indicator	Average time spent by flights in grid i during time window t.
**Speed adjustment frequency**	Fᵢ^spd^(t)	Operational-state indicator	Frequency of observable speed-adjustment behavior in grid i during time window t.
**Heading deviation frequency**	Fᵢ^hdg^(t)	Operational-state indicator	Frequency of heading-deviation or rerouting-related behavior in grid i during time window t.
**Altitude change frequency**	Fᵢ^alt^(t)	Operational-state indicator	Frequency of altitude-change behavior in grid i during time window t.
**Holding instruction frequency**	Fᵢ^hold^(t)	Operational-state indicator	Frequency of holding, circling, or delay-related intervention evidence in grid i during time window t.
**Congestion level**	yᵢ(t)	Label	Three-level congestion pseudo-label generated by K-means++ from direct operational-state indicators.
**Label confidence**	ρᵢ(t)	Diagnostic indicator	Diagnostic confidence of the pseudo-label, retained for sample-quality inspection.
**Weather intensity**	Mᵢ(t)	External factor	Weather-impact intensity mapped to grid i and time window t.
**Restricted-area impact**	Rᵢ(t)	External factor	Restricted-area or temporary airspace-constraint impact mapped to grid i and time window t.
**Controller workload**	Cᵢ(t)	Management factor	Controller workload or management-pressure indicator aggregated to grid i and time window t.
**Wake intensity**	Vᵢ(t)	Wake-related factor	Wake-separation pressure proxy derived from aircraft type mix and local traffic state.

Table 1 defines the final grid-window parameters used in this study. The six operational-state indicators are used for K-means++ congestion labeling and prediction; the external, management, and wake-related factors are retained for prediction and post-hoc attribution; time and grid indices support temporal alignment and graph construction; congestion level and label confidence serve as supervision and diagnostic fields.

Data transformation and 5-min grid-window aggregation. The machine-learning dataset used in this study is not a direct export of raw surveillance or operational records. A data transformation layer first cleans and time-aligns the available surveillance-derived, weather, restricted-area, controller-workload, and wake-related inputs, maps them to the sector grid, and aggregates them into 5-minute grid-window records. Within each valid grid and time window, aircraft movement and intervention evidence are converted into traffic load, average dwell time, speed adjustment frequency, heading deviation frequency, altitude change frequency, and holding instruction frequency; external and management-related inputs are converted into weather intensity, restricted-area impact, controller workload, and wake intensity. The resulting grid-window feature table is then used in two different ways: the six direct operational-state indicators are used for K-means++ congestion-state labeling, while the ten visible raw features are used by the GNN-BiLSTM-Cross-Attention model for 15-minute-ahead prediction. This design separates low-level data engineering from the scientific variables reported in the manuscript: the manuscript focuses on variables that enter clustering and learning, whereas complete source-field definitions are documented in the released data dictionary.

More specifically, the raw inputs comprised time-stamped ADS-B surveillance records, CAAC weather-radar products, active restricted-area records, controller-operation workload data, and wake-separation-related information. The records were first quality checked, deduplicated, filtered by the sector boundary, and transformed into traffic, trajectory, weather, restriction, workload, and wake-related variables. Weather intensity was represented by normalized radar reflectivity and weather coverage by the affected-area ratio; their equal-weight mean was mapped to levels 0–2 for no, slight, and clear impact, while a hard restriction such as severe convection or hail was assigned level 3. The processed variables were then aligned by time and location, and ADS-B positions were mapped to the fixed sector grid. Finally, the aligned observations were aggregated by grid and 5-min time window to form the 15-column grid-window table used for clustering and prediction.

### 3.4. K-means++ based congestion identification model

Based on the established congestion indicator system, an unsupervised K-means++ clustering approach is applied to categorize en-route sector congestion states. Historical grid-level operational data are partitioned in a feature space formed only by the six direct operational-state variables. The external and management-related variables are not used to form the clusters. The resulting clusters provide operationally interpretable congestion levels, which are subsequently used as objective labels for supervised learning.

It should be emphasized that the clustering aims to capture the intrinsic operational state of congestion, rather than all influencing factors. The human-machine-environment-management indicator system constructed in Section 3.3 covers both state indicators and external or management-related factors, while the clustering feature set focuses on direct state characterization. External factors such as weather impact, restricted-area impact, controller workload, and wake intensity may trigger or aggravate congestion, but they do not by themselves define congestion if the observable traffic state remains smooth. Thus, these factors are excluded from the clustering feature vector to avoid diluting the operational-state differences, and are instead used by the prediction model and post-hoc attribution module.

The clustering workflow is as follows:

Step 1: Construct the clustering feature vector for each grid at time t:


$$\begin{array}{c} {𝐱}_{i}^{\left(t\right)}=[Q_{i}(t),{\bar{T}}_{f,i}(t),N_{s,i}(t),N_{h,i}(t),N_{a,i}(t),N_{d,i}(t),K_{\text{3},i}(t){]}^{T}\in R^{\text{7}} \end{array}$$ (1)

The feature vector consists of traffic load, average dwell time, speed adjustment frequency, heading deviation frequency, altitude change frequency, and holding instruction frequency. These indicators directly describe traffic accumulation and tactical control response in each grid window. External or management-related indicators are excluded from this vector because they represent capacity-affecting conditions or explanatory factors rather than the intrinsic congestion state used for unsupervised labeling.

Step 2: Normalize the clustering feature distribution using a training-period z-score standardization:


$$\begin{array}{c} {\tilde{𝐱}}^{\left(m\right)}=\text{d}\text{iag}(\sigma {)}^{-\text{1}}({𝐱}^{\left(m\right)}-\mu ) \end{array}$$ (2)

where μ and σ denote the training-period mean and standard-deviation vectors used by StandardScaler and the sample vector denotes one grid-window observation. The transformation is fitted using the clustering-training segment only. This preprocessing reduces scale imbalance and heavy-tailed effects among traffic-load, dwell-time, and maneuver-frequency variables before K-means++ clustering.

The normalized feature dataset is clustered using the K-means++ algorithm. Compared to classical K-means, K-means++ selects the first cluster center randomly, then chooses subsequent centers with probability proportional to their distance from existing centers, reducing the risk of local minima and improving clustering stability. The algorithm minimizes the within-cluster sum of squares (WCSS):


$$\begin{array}{c} \text{WCSS}(K)=\sum_{k=\text{1}}^{K}\sum_{\tilde{𝐱}\in C_{k}}∥\tilde{𝐱}-{𝐜}_{k}{∥}^{\text{2}} \end{array}$$ (3)

where WCSS(K) denotes the within-cluster sum of squares for K candidate clusters, Ck denotes the k-th cluster, x denotes a normalized grid-window feature vector, and ck denotes the corresponding cluster center. The number of clusters is determined by evaluating candidate values of K using the elbow method, silhouette coefficient, Calinski-Harabasz index, and Davies-Bouldin index. K is not manually assigned from prior labels. In the implemented validation, K = 3 is selected because it provides the best combined internal validation behavior and yields an operationally interpretable free-flow, transitional, and congested state structure.

Step 3: After clustering, the cluster center matrix is obtained. For each grid i at time t, the transformed feature vector is assigned to the nearest cluster based on Euclidean distance, generating the congestion-level label shown in Eq (4):


$$\begin{array}{c} L_{i}^{\left(t\right)}=\text{arg}\underset{k\in \{\text{1},...,K\}}{\text{min}}∥\text{diag}(\sigma {)}^{-\text{1}}({\text{x}}_{i}^{\left(t\right)}-\mu )-{\text{c}}_{k}{∥}^{\text{2}} \end{array}$$ (4)

Based on the congestion-level label obtained from Eq (4), a binary congestion point label is derived by defining a threshold that distinguishes congested grids:


$$\begin{array}{c} Y_{i}^{\left(t\right)}=\text{I}(L_{i}^{\left(t\right)}\geq \theta ) \end{array}$$ (5)

where the binary label equals 1 when grid i at time t is classified as a key congestion point, and I(.) is the indicator function.

To provide an intuitive understanding of the congestion level identification process, Fig 4 visualizes the K-means++ clustering results based on historical grid-level operational features.

**Fig 4:**
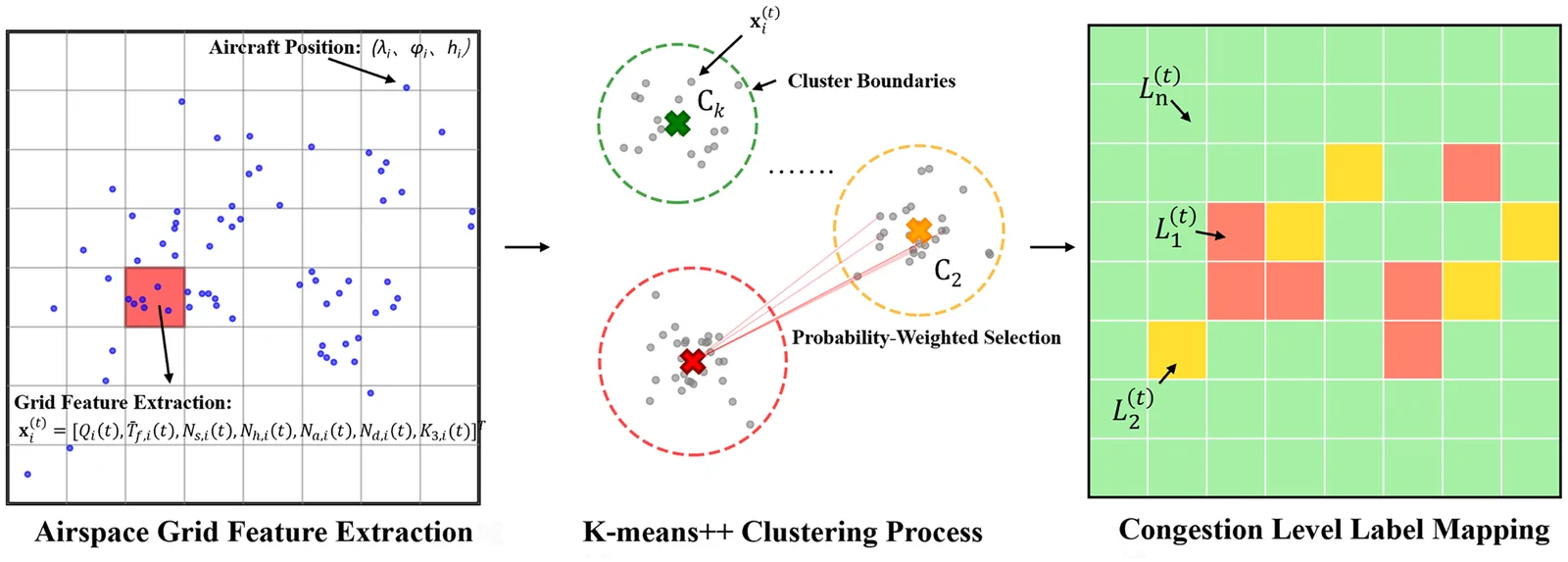
K-means++ clustering results for congestion level identification.

In summary, K-means++ clustering converts historical grid-level operational-state indicators into three congestion labels, providing objective supervision for subsequent learning-based models. External and management-related factors remain available to the prediction model and the SHAP/SHEP attribution layer, but they do not determine the clustering labels. Through the above modeling, en-route sector congestion is transformed from an abstract operational concept into a structured, quantitative, and physically interpretable state representation for the spatiotemporal prediction model introduced in the next section.

## 4. Deep learning model for congestion prediction and attribution

### 4.1. Spatiotemporal congestion prediction via GNN-BiLSTM-cross-attention model

Based on the congestion mechanisms and quantitative state representations established in Section 3, this section develops an integrated spatiotemporal prediction framework for en-route sector congestion. The objective is to predict 15-minute-ahead congestion states by jointly modeling spatial interactions among airspace grids, temporal evolution in recent grid-window sequences, and visible external or management-related factors.

Although static congestion level labels derived from historical data provide supervisory targets for prediction, they are insufficient to capture the dynamic propagation of congestion across airspace and its spatiotemporal evolution. Conventional sector-level analysis often obscures localized operational variations, limiting the model’s ability to identify fine-grained mechanisms such as congestion diffusion and inter-grid traffic interactions.

To address these limitations, this study constructs a deep learning model that takes a spatiotemporal graph sequence as input and explicitly models spatial correlations, temporal dependencies, and multi-factor interactions to enable accurate 15-minute-ahead congestion prediction. Fig 5 illustrates the architecture of the proposed framework, including graph-attention spatial encoding, BiLSTM-based temporal encoding, cross-attention fusion, two supervised prediction heads for congestion-level estimation and T2 hotspot localization, and an inference-stage SHAP/SHEP grouped attribution module for explaining predicted warnings.

**Fig 5:**
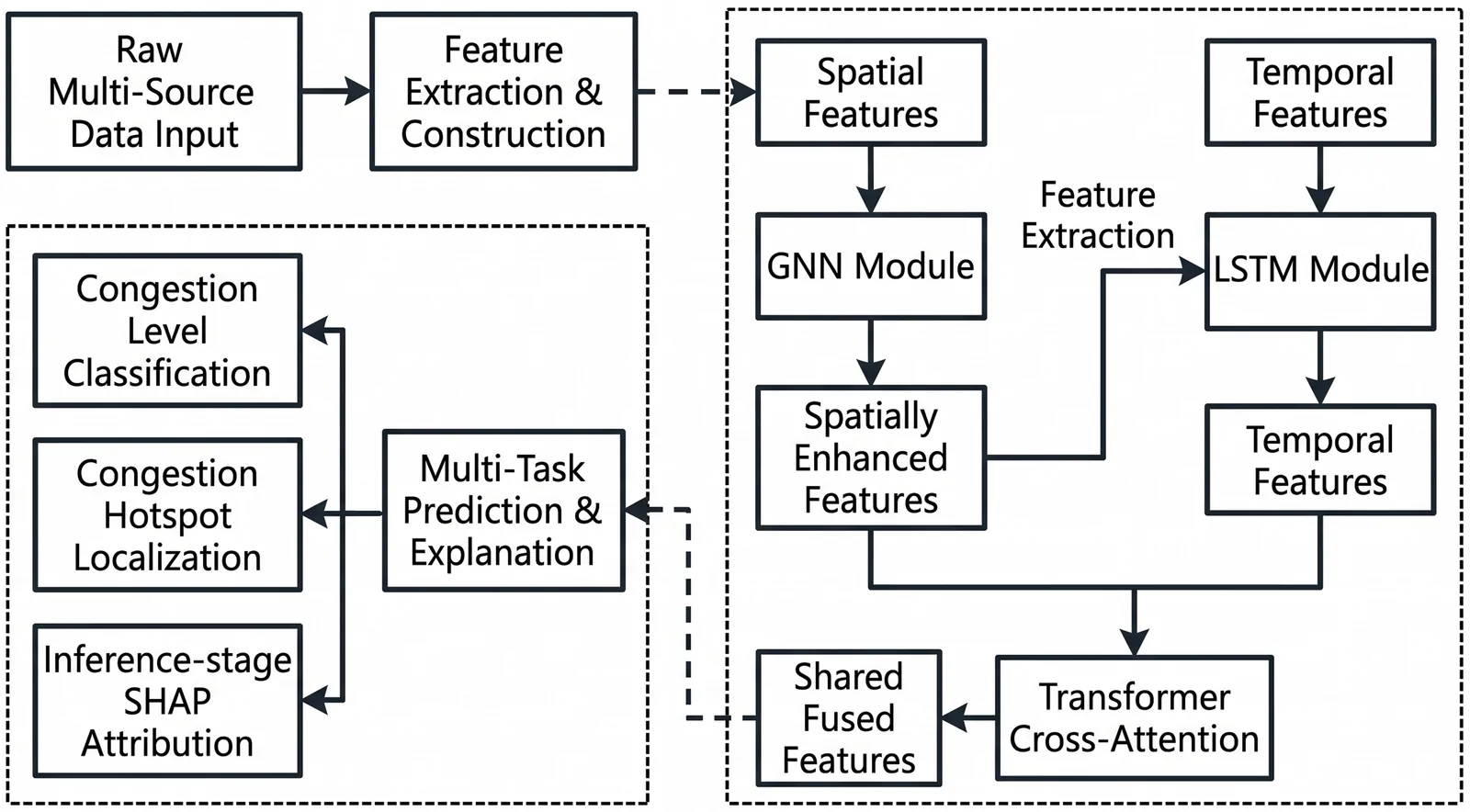
Deep Learning Architecture for Sector Congestion Prediction and Post-Hoc Attribution.

The standard grid-window record contains 15 columns: time step, grid row, grid column, six operational-state indicators, congestion level, label confidence, and four external or management-related factors. The model-visible raw features are the six operational indicators (traffic load, average dwell time, speed adjustment frequency, heading deviation frequency, altitude change frequency, and holding instruction frequency) and the four external or management-related factors (weather intensity, restricted-area impact, controller workload, and wake intensity). Time step and grid coordinates are used for indexing, window alignment, and graph construction, while congestion level and label confidence are used for supervision and diagnostics rather than direct model input.

For each prediction sample, the model forms a historical graph-sequence tensor from the current and previous 5-minute grid windows. The ten visible raw features are processed inside the data loader into normalized feature channels, static grid or route-location channels, one-step lagged values, and first-difference values. These auxiliary channels are deterministic transformations of the available historical window and fixed grid metadata; no future observed feature or manually specified congestion-propagation score is used as an input.

As shown in Fig 5, the model learns a spatiotemporal mapping function that predicts the congestion state of each grid at a future time step with prediction horizon = 3, corresponding to a 15-minute warning horizon:


$$\begin{array}{c} p_{i}(t+\text{3}),q_{i}(t+\text{3})=f_{\Theta }(X_{i}(t-L+\text{1}:t),A) \end{array}$$ (6)

where the historical graph-sequence tensor denotes grid i over the most recent L time windows, A denotes the spatial or route-related adjacency matrix, the first output denotes the predicted three-class congestion probability vector, and the second output denotes the predicted T2 hotspot probability for grid i. Here L is the historical sequence length and t + 3 corresponds to the 15-minute prediction horizon. The supervised parameters are learned with two prediction heads, while attribution is computed after inference and is not treated as a supervised regression target.

Operationally, the 15-minute horizon is selected as a compromise between warning lead time and forecast reliability. With five-minute aggregation, it corresponds to three future steps, which provides enough lead time to identify the buildup and spatial spread of congestion before the next few tactical decision cycles, while limiting uncertainty growth in a rapidly changing en-route traffic state. It also matches the rolling-update logic of the framework: new observations can be incorporated every five minutes, while a 15-minute warning remains actionable for short-term ATFM coordination, such as speed control, metering, rerouting coordination, or preparation for holding. The horizon is therefore intended for tactical pre-warning rather than long-horizon strategic planning.

The inference-stage explanation output is defined as:


$$\begin{array}{c} \Phi_{i,t+\text{3}}\;=\;\text{E}(p_{i,t+\text{3}},\;q_{i,t+\text{3}},\;X_{i}(t-L+\text{1}:t),\;A) \end{array}$$ (7)

where the attribution output denotes the post-hoc grouped explanation result for grid i at the target time, and E(.) denotes the inference-stage explanation operator applied to the predicted class probabilities, hotspot probability, historical input window, and graph adjacency. This term is used only for explanation and visualization after prediction; it is not a training target and does not introduce future observed features.

### 4.2. Graph neural network-based spatial feature extraction

Spatial dependencies among grid cells are first modeled to capture congestion propagation across the sector airspace. To enable graph-based spatial feature extraction, the gridded sector is transformed into a graph representation suitable for deep learning. At each time step, the airspace state graph is defined as the model input.


$$\begin{array}{c} G_{t}=(V,E,X_{t},A) \end{array}$$ (8)

where X_t_ is the node feature matrix at time t, with each row formed from the model-visible features and historical-window-derived channels of grid i, and A is a spatial or route-related adjacency matrix constructed from grid-neighborhood relations, route contiguity, and observed flow-transfer evidence. This design preserves local spatial context while allowing observed traffic-flow relations to guide congestion propagation modeling.

Each grid cell is treated as a graph node. Edges are retained when grids are geographically adjacent, route-contiguous, or supported by observed flight-flow transfer in the historical data. The corresponding normalized edge weight can be expressed as:


$$\begin{array}{c} a_{ij}=\frac{n_{ij}}{\sum_{k}n_{ik}} \end{array}$$ (9)

where nᵢⱼ denotes the count of flights moving from grid i to grid j during the aggregation interval. This yields a row-stochastic adjacency matrix that captures directional traffic propagation patterns. The graph is inherently sparse because only grid pairs connected by observed flight movements, route contiguity, or local neighborhood relations have non-zero weights.

In the graph attention mechanism, the neighborhood N(i) of node $v_{i}$ is defined to include all nodes $v_{j}$ with $A_{ij}>\text{0}$ as well as the node itself. This allows the model to retain and adaptively combine the node’s own features with those of its neighbors. The adjacency matrix is not symmetrized, preserving the directed nature of air traffic flows.

In the 5-minute rolling setting, some grids may temporarily have no outgoing or incoming flight-flow edges. These isolated or weakly connected nodes are not removed. Self-loops are retained for every valid grid, and static neighborhood or route-contiguity information is used together with observed flight-flow edges so that each node can preserve its own operational state and receive local spatial context when dynamic flow transfer is sparse. Under very low traffic volume, the graph branch contributes less propagation information and the prediction relies more on node-level operational features, recent temporal history, weather, restricted-area, controller-load, and wake-intensity variables. This treatment prevents isolated nodes from producing empty graph aggregations while preserving the directed nature of actual traffic-flow propagation.

After the grids are defined, congestion in one grid may propagate along flight routes and influence neighboring or upstream grids. To model this spatial dependency, a graph attention network is adopted. For node i, the original feature vector is denoted by hᵢ^(o)^, and hᵢ^(l)^ denotes the node representation at layer l. The feature update at layer l proceeds as follows:

First, the forward propagation for the entire GNN layer can be expressed as a matrix operation:


$$\begin{array}{c} H^{\left(t,l\right)}=\sigma \left(\text{softmax}\left(\text{LeakyReLU}\left(A⊙\left(\text{1}\cdot (W^{\left(l\right)}H^{\left(t,l-\text{1}\right)}{)}^{T}\cdot {\vec{a}}^{\left(l\right)}\right)\right)\right)\cdot (W^{\left(l\right)}H^{\left(t,l-\text{1}\right)})\right) \end{array}$$ (10)

In Eq. (10), ⊙ denotes element-wise multiplication, and **1** is an all-ones vector of compatible dimension. The index *t* denotes the time step in the historical five-minute sequence, *l* denotes the graph layer, and *H*^*(t,l)*^ denotes the node representation at time *t* after graph layer *l*. The adjacency matrix *A* remains static across graph snapshots, while temporal variation is represented by the node-feature sequence and the time-indexed hidden representations.

Next, the unnormalized attention score between node i and its neighboring node j is computed as:


$$\begin{array}{c} e_{ij}^{\left(t,l\right)}=\text{LeakyReLU}\left({\vec{a}}^{(l)T}\left[W^{\left(l\right)}h_{i}^{\left(t,l-\text{1}\right)}∥W^{\left(l\right)}h_{j}^{\left(t,l-\text{1}\right)}\right]\right) \end{array}$$ (11)

where a^(l)^ is a learnable attention vector for layer l, W^(l)^ is the learnable weight matrix of layer l, ∥ denotes vector concatenation, and LeakyReLU is the nonlinear activation function.

The attention scores are then normalized using the softmax function to obtain attention coefficients:


$$\begin{array}{c} \alpha_{ij}^{\left(t,l\right)}=\frac{\text{exp}(e_{ij}^{\left(t,l\right)})}{\sum_{k\in \text{N}(i)}\text{exp}(e_{ik}^{\left(t,l\right)})} \end{array}$$ (12)

where N(i) is the set of neighboring nodes of node i, including the node itself.

Based on the attention coefficients, the node representation is updated by aggregating neighbor information:


$$\begin{array}{c} h_{i}^{(t,l{)}^{\prime }\;}=\sigma \left(\sum_{j\in \text{N}(i)}\alpha_{ij}^{\left(t,l\right)}W^{\left(l\right)}h_{j}^{\left(t,l-\text{1}\right)}\right) \end{array}$$ (13)

where σ(·) denotes the activation function.

To enhance model capacity and stability, a multi-head attention mechanism is employed. Let H denote the number of attention heads; the output of layer l is:


$$\begin{array}{c} h_{i}^{\left(t,l\right)}={∥}_{k=\text{1}}^{K}\sigma \left(\sum_{j\in \text{N}(i)}\alpha_{ij}^{\left(t,l,k\right)}W^{\left(l,k\right)}h_{j}^{\left(t,l-\text{1}\right)}\right) \end{array}$$ (14)

where αᵢⱼ^(^ʰ^)^ is the attention coefficient of the h-th head and W^(^ʰ^)^ is the corresponding head-specific weight matrix.

To further improve training stability and model performance, residual connections and layer normalization are introduced:


$$\begin{array}{c} {\tilde{h}}_{i}^{\left(t,l\right)}=\text{LayerNorm}\left(h_{i}^{\left(t,l\right)}+h_{i}^{\left(t,l-\text{1}\right)}\right) \end{array}$$ (15)

where LayerNorm denotes the layer normalization operation.

After $L_{g}$ layers of propagation, the spatially enhanced feature matrix is obtained:


$$\begin{array}{c} H_{s}^{\left(t\right)}=H^{\left(t,L_{g}\right)}={\left[h_{\text{1}}^{\left(t,L_{g}\right)},h_{\text{2}}^{\left(t,L_{g}\right)},\ldots ,h_{N}^{\left(t,L_{g}\right)}\right]}^{T}\in R^{N\times d_{\text{out}}} \end{array}$$ (16)

where d_out_ is the output feature dimension. This feature matrix contains the intrinsic information of each grid and aggregates contextual information from its spatial neighborhood, providing a representation for understanding congestion propagation patterns.

The adjacency design is intentionally conservative. Direct flight-flow transitions capture observable traffic propagation, but they may miss indirect coupling caused by controller workload balancing, rerouting constraints, common weather impacts, or restricted-area avoidance. These indirect relationships are partly represented through node features and static route-contiguity context. Future work can extend the current graph to a dynamic multi-relation graph that jointly represents flight-flow transfer, geographic adjacency, airway topology, and shared operational constraints.

To facilitate understanding of the graph-based spatial modeling process, Fig 6 illustrates the overall *l*-layer GNN structure and the grid-wise feature update and information aggregation procedure within a single GNN layer.

**Fig 6:**
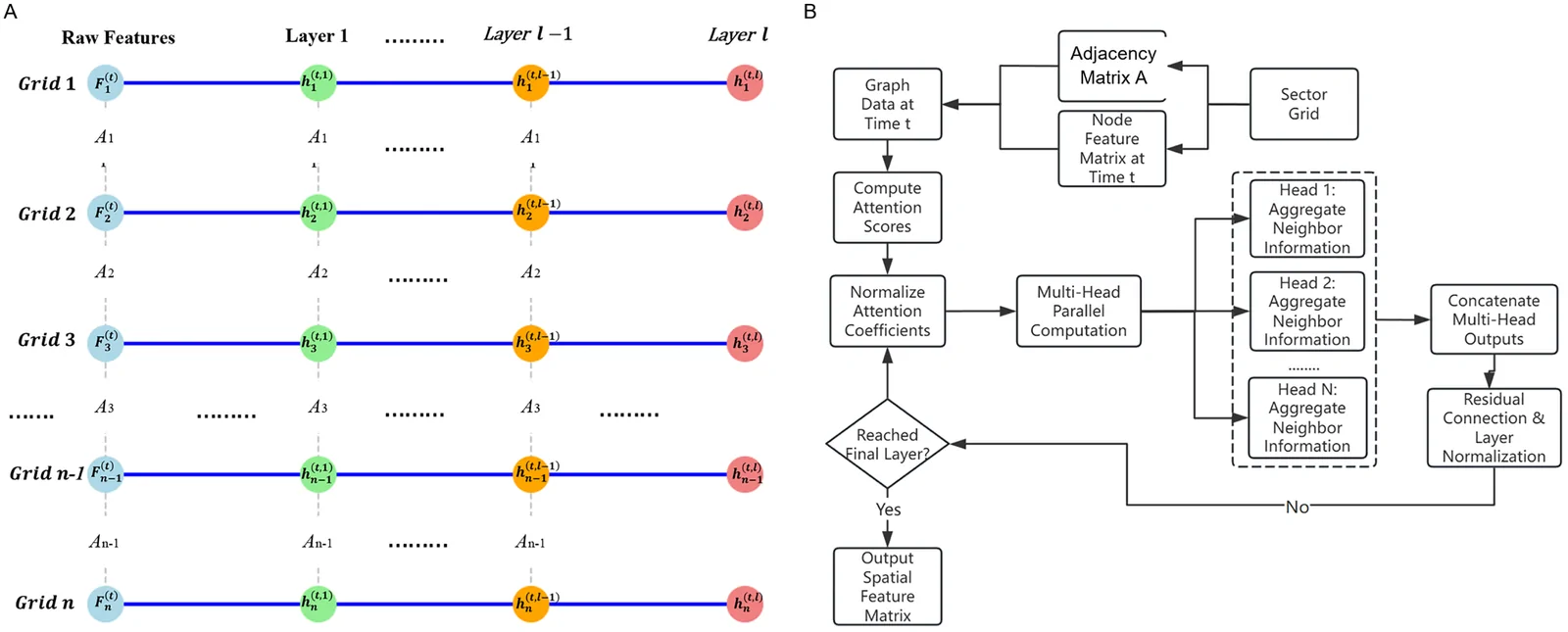
Graph Neural Network structure and update procedure.

Fig 6 highlights how multi-hop spatial dependencies among grids are progressively captured through stacked GNN layers, together with the attention-weighted aggregation mechanism that enables adaptive modeling of congestion propagation.

### 4.3. LSTM-based temporal feature extraction

While spatial modeling captures inter-grid interaction effects at each time step, congestion evolution also exhibits strong temporal dependencies, motivating further temporal sequence modeling.

For each grid node i, the time-series output from the GNN layer is fed into the BiLSTM. Let hᵢ,_t_^(l)^ denote the spatially enhanced node feature of grid i at time t from the l-th GNN layer. With historical sequence length L, the input sequence for grid i is {hᵢ,_t_ − L + 1^(l)^, …, hᵢ,_t_^(l)^}. The forward LSTM processes the sequence from t − L + 1 to t, while the backward LSTM traverses it in the reverse direction. Let d_h_ denote the hidden-state dimension. The forward LSTM computations at time step τ are as follows:

The input gate $i_{t}^{\to }\;$controls the extent of new information to be added:


$$\begin{array}{c} I_{t}^{\to }=\sigma \left(W_{i}^{\to }\cdot \left[h_{t-\text{1}}^{\to },h_{i}^{\left(t,L_{g}\right)}\right]+b_{i}^{\to }\right) \end{array}$$ (17)

where σ(·) is the sigmoid activation function; Wᵢ and bᵢ are the input-gate weight matrix and bias vector, respectively; and Θ denotes the learnable parameter set of the LSTM.

The forget gate $f_{t}^{\to }$determines the retention of previous memory:


$$\begin{array}{c} F_{t}^{\to }=\sigma \left(W_{f}^{\to }\cdot \left[h_{t-\text{1}}^{\to },h_{i}^{\left(t,L_{g}\right)}\right]+b_{f}^{\to }\right) \end{array}$$ (18)

Here, *W*_*f*_ and *b*_*f*_ are the forget-gate weight matrix and bias vector of the forward LSTM, both belonging to Θ.

Output gate $o_{t}^{\to }\;$controls how much information is output to the hidden state:


$$\begin{array}{c} O_{t}^{\to }=\sigma \left(W_{o}^{\to }\cdot \left[h_{t-\text{1}}^{\to },h_{i}^{\left(t,L_{g}\right)}\right]+b_{o}^{\to }\right) \end{array}$$ (19)

Here, *W*_*o*_ and *b*_*o*_ are the output-gate weight matrix and bias vector of the forward LSTM, both belonging to Θ.

Candidate memory ${\tilde{C}}_{t}^{\to }\;$is an intermediate memory state providing new information to be selectively added to the cell:


$$\begin{array}{c} {\hat{\text{C}}}_{t}^{\to }=\text{tanh}\left(W_{C}^{\to }\cdot \left[h_{t-\text{1}}^{\to },h_{i}^{\left(t,L_{g}\right)}\right]+b_{C}^{\to }\right) \end{array}$$ (20)

Here, tanh(·) denotes the hyperbolic tangent activation function; *W*_*c*_ and *b*_*c*_ are the candidate-memory weight matrix and bias vector, both belonging to Θ.

Cell state $C_{t}^{\to \;}$stores long-term memory:


$$\begin{array}{c} C_{t}^{\to }=f_{t}^{\to }⊙C_{t-\text{1}}^{\to }+i_{t}^{\to }⊙{\hat{\text{C}}}_{t}^{\to } \end{array}$$ (21)

Hidden state $h_{t}^{\to }\;$is the short-term memory output:


$$\begin{array}{c} h_{t}^{\to }=o_{t}^{\to }⊙tanh\left(C_{t}^{\to }\right) \end{array}$$ (22)

The forward LSTM update process is illustrated in Fig 7. The backward LSTM performs similar computations but in the reverse temporal direction:

**Fig 7:**
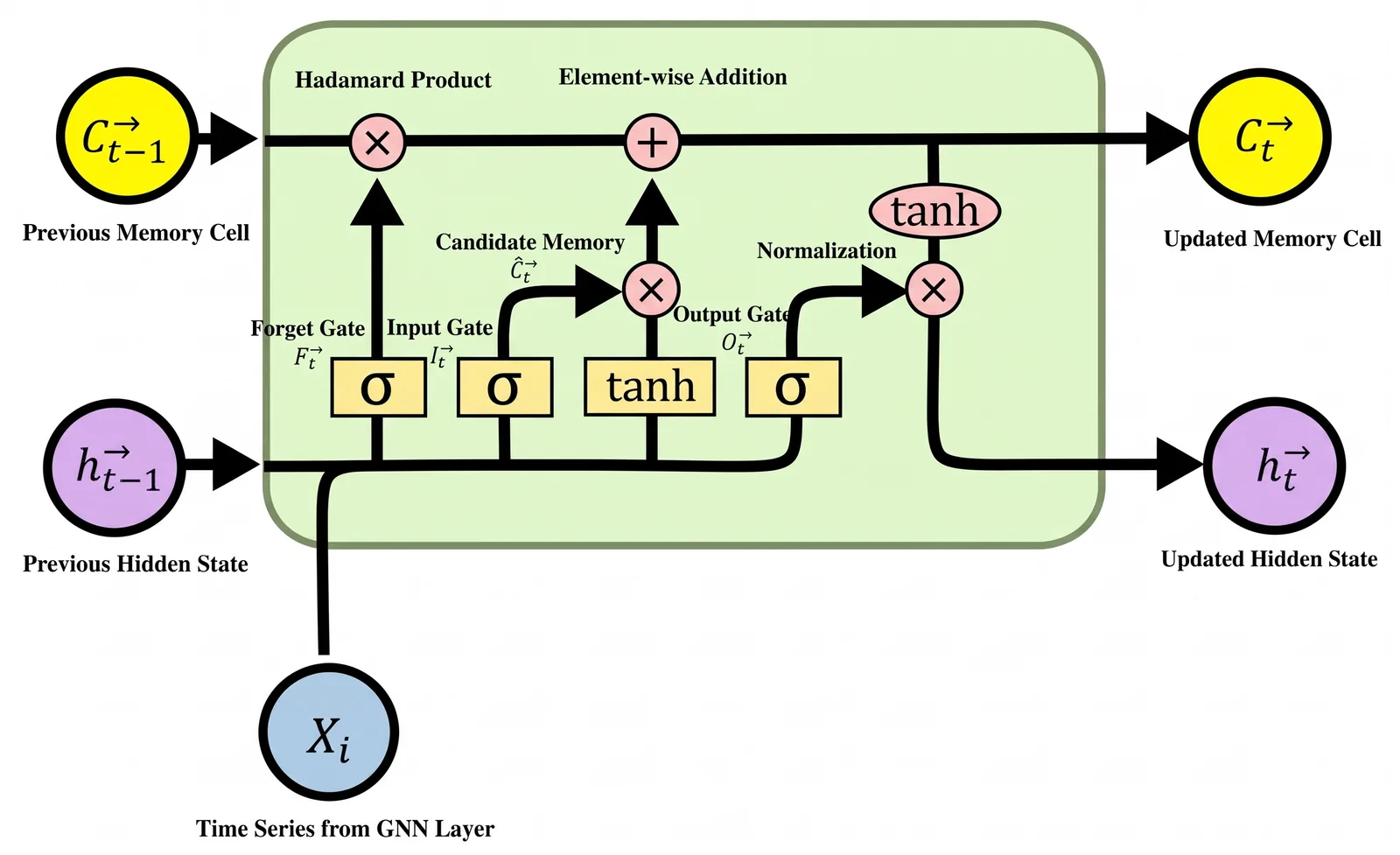
Forward LSTM Model Update Process.

Finally, the forward LSTM’s final hidden state $h_{T_{\text{in}}\;}^{\to }$and the backward LSTM’s initial hidden state $h_{\text{1}}^{\leftarrow }$are concatenated to obtain the temporal feature for each grid node:


$$\begin{array}{c} h_{i}^{\prime }\;=\;[h_{T_{in}}^{\to }\left\|h_{\text{1}}^{\leftarrow }\right]\in R^{\text{2}d_{h}} \end{array}$$ (23)

The temporal features of all grid nodes form the matrix H^T^, where each row corresponds to one grid node and each column block corresponds to the BiLSTM hidden representation:


$$\begin{array}{c} H_{t}={\left[h_{\text{1}}^{\prime },h_{\text{2}}^{\prime },\ldots ,h_{N}^{\prime }\right]}^{T}\in R^{N\times \text{2}d_{h}} \end{array}$$ (24)

Each LSTM unit selectively updates the memory cell state through input, forget, and output gates, effectively modeling the accumulation, persistence, and dissipation of congestion within the historical window. Concatenating the forward and backward hidden representations produces a temporal feature matrix that captures recent buildup and relaxation patterns, providing the basis for subsequent spatiotemporal feature fusion.

### 4.4. Cross-attention-based spatiotemporal feature fusion, prediction heads, and attribution

To jointly exploit the complementary spatial and temporal representations learned in Sections 4.2 and 4.3, a feature fusion and prediction module is designed to generate future sector congestion state estimates.

The key idea behind the fusion module is to leverage the temporal features as queries to attend to the spatial features. This design allows the model to dynamically weight spatial correlations based on temporal patterns, thereby capturing how historical congestion trends influence the current spatial configuration and enabling more accurate prediction of future states.

Let the LSTM-encoded temporal feature matrix and the GNN-encoded spatial feature matrix be the two inputs of the fusion module. Linear projections first map them to queries, keys, and values:


$$\begin{array}{c} Q\;=\;H_{tem}\;W_{Q} \end{array}$$ (25)

$$\begin{array}{c} K\;=\;H_{spa}\;W_{K} \end{array}$$ (26)

$$\begin{array}{c} V\;=\;H_{spa}\;W_{V} \end{array}$$ (27)

where the temporal feature matrix and spatial feature matrix are denoted by Htem and Hspa, respectively; Q, K, and V denote the query, key, and value matrices; and WQ, WK, and WV are learnable projection matrices. The key and value dimensions are defined consistently with the hidden representation size.

The cross-attention is computed via the scaled dot-product attention mechanism:


$$\begin{array}{c} A_{att}\;=\;\text{softmax}(QKT\;/\;\text{sqrt}(d_{k})) \end{array}$$ (28)

$$\begin{array}{c} O\;=\;A_{att}\;V \end{array}$$ (29)

where Aatt denotes the attention-weight matrix, dk denotes the key dimension used for scaling, and O denotes the cross-attention output before multi-head concatenation.

To enhance representational capacity, multi-head attention (MHA) is applied by splitting queries, keys, and values into multiple heads along the feature dimension, computing attention for each head, and then concatenating the results:


$$\begin{array}{c} {head}_{h}\;=\;\text{Attention}(QW_{Q,h},\;KW_{K,h},\;VW_{V,h}) \end{array}$$ (30)

$$\begin{array}{c} MHA\;=\;\text{Concat}({head}_{\text{1}},...,\;{head}_{H})W_{O} \end{array}$$ (31)

where head h denotes the h-th attention head, H denotes the number of heads, WO denotes the output projection matrix, and Concat(.) denotes feature concatenation across heads.

To enable residual connection, the output dimension of the multi-head attention is set to match the hidden dimension of the BiLSTM:


$$\begin{array}{c} H_{a}\;=\;\text{LayerNorm}(H_{tem}\;+\;MHA) \end{array}$$ (32)

A feedforward network and a second residual normalization step are then applied to obtain the fused node representation used by the prediction heads:


$$\begin{array}{c} H_{f}\;=\;\text{FFN}(H_{a}) \end{array}$$ (33)

$$\begin{array}{c} z\;=\;\text{LayerNorm}(H_{a}\;+\;H_{f}) \end{array}$$ (34)

where Ha is the residual attention representation, Hf is the feedforward-transformed representation, and z denotes the final fused node representation passed to the classification and hotspot heads.

The output encodes high-level node representations that integrate spatiotemporal context, dynamically weighting historical temporal patterns and spatial correlations for each grid. Fig 8 illustrates the spatiotemporal cross-attention fusion mechanism, which enables the model to adaptively combine spatial and temporal evidence rather than relying on simple feature concatenation.

**Fig 8:**
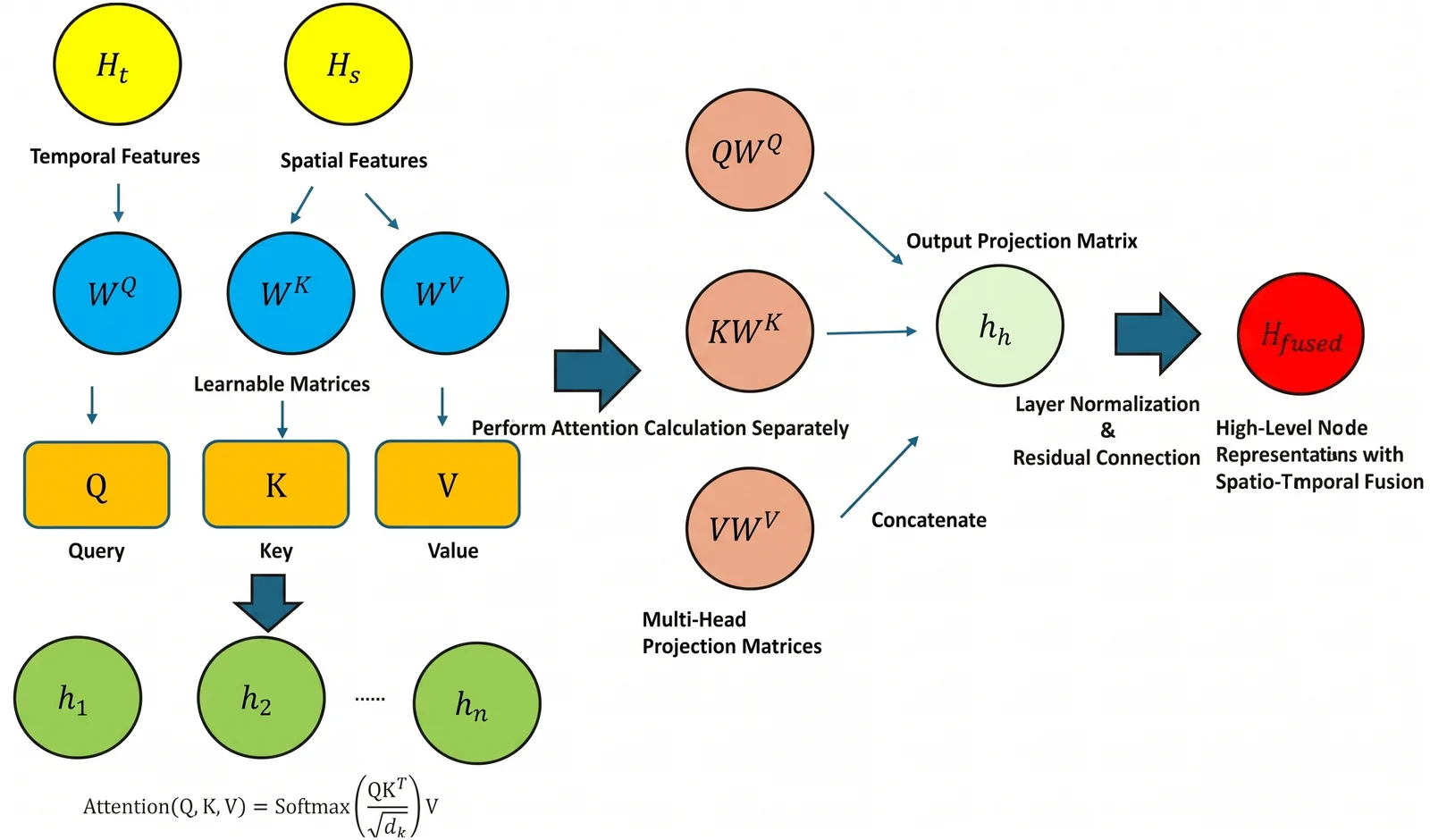
Spatiotemporal Cross-Attention Fusion.

Based on the shared fused feature, the model employs two lightweight supervised decoding networks. The first head outputs the future congestion-level probability distribution for T0, T1, and T2, and the second head outputs the future T2 hotspot probability for each grid. The former supports three-level congestion prediction, while the latter focuses on rare high-congestion warning under class imbalance.

Congestion Level Classification Head: for the fused representation zᵢ,_t_ of grid i, a multilayer perceptron produces the three-class logits and the Softmax probabilities:


$$\begin{array}{c} o_{i,t+\text{3}}\;=\;{\text{MLP}}_{c}(z_{i,t}),\;p_{i,t+\text{3}}\;=\;\text{Softmax}(o_{i,t+\text{3}}) \end{array}$$ (35)

The predicted congestion level is selected by the maximum posterior probability:


$$\begin{array}{c} {\hat{y}}_{i,t+\text{3}}\;=\;{\text{argmax}}_{k}\;p_{i,t+\text{3},k} \end{array}$$ (36)

Congestion Hotspot Localization Head: a second multilayer perceptron estimates whether grid i will become a high-congestion hotspot at t + 3:


$$\begin{array}{c} q_{i,t+\text{3}}\;=\;\text{sigmoid}({\text{MLP}}_{h}(z_{i,t})) \end{array}$$ (37)

SHAP (SHapley Additive exPlanations)-style post-hoc perturbation: after the trained prediction model produces congestion-level and hotspot probabilities, attribution is generated as an inference-stage explanation layer. For a target grid and prediction window, each model-visible feature or feature group g is replaced by a reference value from the same input window, and the decrease in predicted T2 probability is used as the warning evidence of that group:


$$\begin{array}{c} \Delta_{g}\;=\;\text{max}(P_{T\text{2}}(x-\;P_{T\text{2}}(x_{g\leftarrow rg}),\;\text{0}) \end{array}$$ (38)

where x is the original input window, *x*_*g←rg*_ denotes the perturbed window in which feature group g is replaced by its reference value *r*_*g*_, and *P*_*T2*_(·) denotes the predicted probability of the congested state. This SHAP-style calculation is applied only after prediction, does not create training labels, and does not introduce future information.

Supervised Loss Function and Optimization: the model is trained by minimizing the weighted sum of a focal congestion-level classification loss and a focal hotspot localization loss:


$$\begin{array}{c} L\;=\;\lambda_{c}\;L_{cls}\;+\;\lambda_{h}\;L_{hotspot} \end{array}$$ (39)

where *L*_*cls*_ is the class-weighted focal loss for three-level congestion classification, *L*_*hotspot*_ is the focal binary cross-entropy loss for T2 hotspot localization, and *λ*_*c*_ and *λ*_*h*_ are loss weights for the classification and hotspot heads. The hotspot term is weighted to improve rare high-congestion warning under class imbalance. No supervised attribution-regression loss is used.

In the implementation, L_cls is the three-class focal loss for T0, T1, and T2, with class weights derived from the training-segment class frequencies and the focal parameter gamma_cls = 1.5. L_hotspot is the binary focal loss for the T2 hotspot target, with alpha_hotspot = 0.80 and gamma_hotspot = 2.0. Both terms are evaluated only on valid target-grid entries and normalized over the corresponding valid weighted samples. The effective objective in this paper configuration is L = L_cls + 0.50 L_hotspot; auxiliary ranking, onset, and attribution-regression losses are not enabled.

SHEP (two-level grouped warning-source explanation) attribution: for operational visualization, the feature-level warning evidence is summarized by a two-level SHEP grouping scheme. The first-level chart separates External Triggering risk from Congestion Propagation risk. External Triggering is calculated from weather intensity, restricted-area impact, controller workload, and wake intensity. Congestion Propagation is calculated from observable traffic-state deterioration, including traffic load, average dwell time, speed adjustment frequency, heading deviation frequency, altitude change frequency, and holding instruction frequency. Neighboring or upstream congestion is reflected through the graph structure and recent grid-window context rather than introduced as a separate raw input column.


$$\begin{array}{c} S_{ext}\;=\;\Delta_{weather}\;+\;\Delta_{restricted}\;+\;\Delta_{control}\;+\;\Delta_{wake} \end{array}$$ (40)

$$\begin{array}{c} S_{prop}\;=\;\Delta_{traffic}\;+\;\Delta_{dwell}\;+\;\Delta_{speed}\;+\;\Delta_{heading}\;+\;\Delta_{altitude}\;+\;\Delta_{holding} \end{array}$$ (41)

$$\begin{array}{c} \alpha_{ext}\;=\;\frac{S_{ext}}{\left(S_{ext}\;+\;S_{prop}\right)},\;\alpha_{prop}\;=\;\frac{S_{prop}}{\left(S_{ext}\;+\;S_{prop}\right)} \end{array}$$ (42)

where the external-triggering score and congestion-propagation score denote the two first-level SHEP groups, respectively; each Delta term denotes the non-negative warning evidence attributed to the corresponding visible feature group; and the two alpha terms are the normalized first-level SHEP proportions. The second-level chart decomposes only the external-triggering group into weather, restricted-area, controller-workload, and wake-intensity contributions.

The second-level chart further decomposes only the External Triggering component into weather, restricted area, controller workload, and wake intensity. In the implementation, SHAP-style perturbation estimates the model sensitivity of visible feature groups after inference, while SHEP summarizes the warning evidence into operational groups. The first-level SHEP index normalizes External Triggering risk against Congestion Propagation risk, and the second-level index normalizes the four external factors within the external component. In this way, the visualization answers two operational questions: whether a warning is mainly externally triggered or propagation-driven, and, if external factors are important, which external or management-related factor dominates.

## 5. Simulation experiments and result analysis

To evaluate the proposed congestion prediction framework, a series of simulation experiments are conducted using 2023 de-identified processed grid-level operational data from the ZGGGAR15 high-altitude en-route sector. The sector is a representative busy en-route control sector in the Guangzhou area control environment, with dense traffic demand and frequent congestion risk, making it a challenging test case for congestion prediction. Model training and inference are implemented in Python using PyTorch and PyTorch Geometric, with GPU acceleration enabled to support efficient spatiotemporal learning. Congestion level labels are generated through K-means++ clustering of direct operational-state features, and the resulting labels are used as supervisory signals for training the proposed spatiotemporal prediction model. Model performance is assessed in terms of clustering validity, rare-congestion warning accuracy, robustness, replay-case visualization, and deployment feasibility.

### 5.1. Spatiotemporal distribution of sector flight flow characteristics

To support model configuration and training, an exploratory analysis is conducted to examine the key spatiotemporal characteristics of sector traffic flow. Rather than providing an exhaustive descriptive analysis, this subsection focuses on identifying data patterns that directly motivate the design of the proposed spatial–temporal congestion prediction framework.

Fig 9 illustrates the hourly distribution of flight volume in the ZGGGAR15 sector, revealing pronounced intra-day variability and peak-hour concentration, which motivates the use of short-term temporal modeling for congestion prediction.

**Fig 9:**
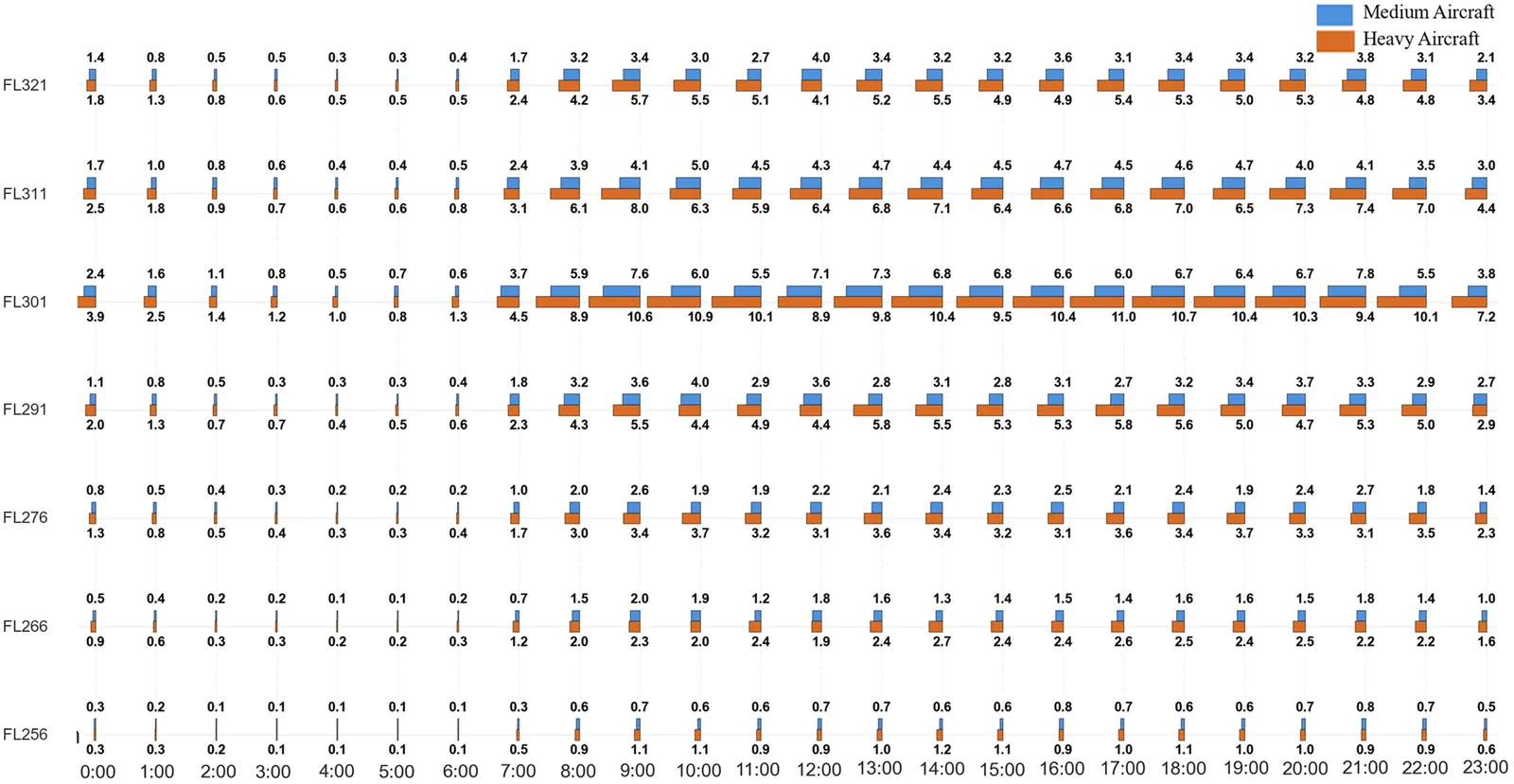
Flight Hour Distribution in the ZGGGAR15 Sector.

As shown in Fig 9, Sector congestion exhibits pronounced temporal variability at multiple time scales. At the intra-day level, the hourly distribution of flight volume shows clear diurnal patterns, with traffic mainly concentrated between 08:00 and 21:00 and distinct daytime peaks. This strong intra-day fluctuation indicates that congestion risk evolves rapidly over time and is highly sensitive to recent traffic conditions.

The distribution of aircraft across altitude layers shows that medium-sized aircraft dominate the core operating altitude range during daytime peak hours, while heavy aircraft, although fewer in number, impose stricter wake turbulence separation requirements. The coexistence of heterogeneous aircraft types within overlapping altitude layers increases operational complexity and reduces effective sector capacity during high-demand periods. This indicates that short-term congestion evolution is driven not only by traffic intensity but also by operational heterogeneity, which motivates the incorporation of complexity-related indicators into temporal feature modeling.

At a longer temporal scale, monthly variations in flight volume further reveal non-uniform traffic demand throughout the year.

As shown in Fig 10, the monthly flight volume exhibits pronounced intra-annual variability, indicating that congestion evolution is influenced not only by short-term dynamics but also by longer-term variations in traffic demand across different months. Flight volumes fluctuate substantially throughout the year, reflecting changes in operational intensity over time. However, the emergence and severity of congestion are not solely driven by traffic volume. The estimated congestion time shows discernible deviations from a purely volume-dependent trend, particularly during periods such as the summer months, when adverse weather and corresponding capacity reductions may play an important role. These observations affirm that congestion evolution is governed by multi-scale spatiotemporal dependencies, encompassing seasonal demand patterns, short-term traffic fluctuations, and real-time environmental or operational disruptions. Therefore, an effective prediction framework should capture temporal demand variation, spatial propagation, and multi-factor interactions, as represented by the proposed GNN-BiLSTM-Cross-Attention architecture.

**Fig 10:**
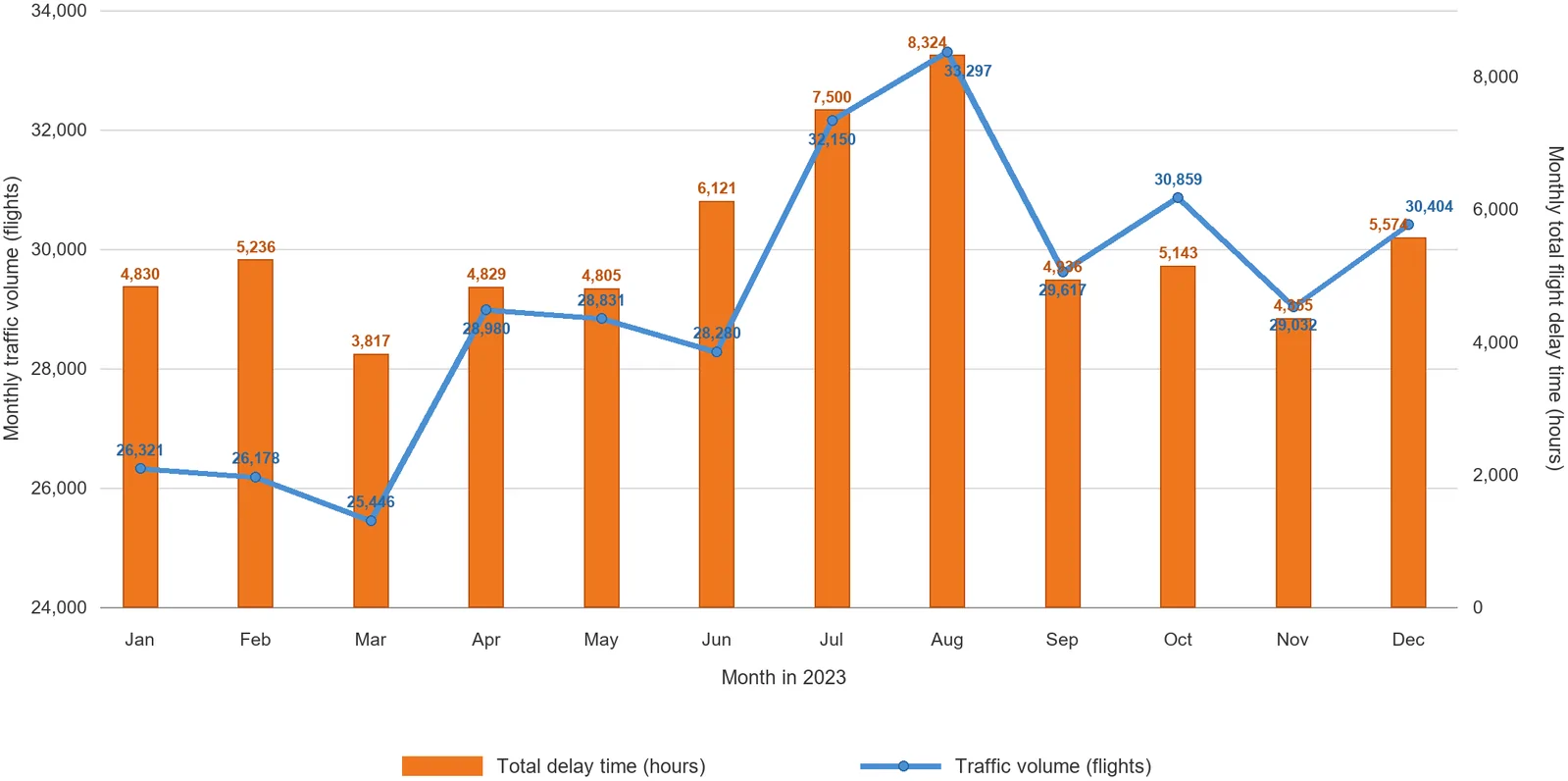
Monthly total flight delay time and traffic volume in the ZGGGAR15 sector (2023).

In addition to temporal variability, the spatial modeling of the sector requires an explicit and structured representation of airspace geometry. Fig 11 shows the horizontal boundary of the ZGGGAR15 sector and its seven processed boundary vertices. The sector polygon was reconstructed from the processed spatial coordinates used in this study and plotted over public-domain Natural Earth boundary data. This figure provides a clear geometric reference for the studied airspace and serves as the physical basis for subsequent spatial discretization and modeling.

**Fig 11:**
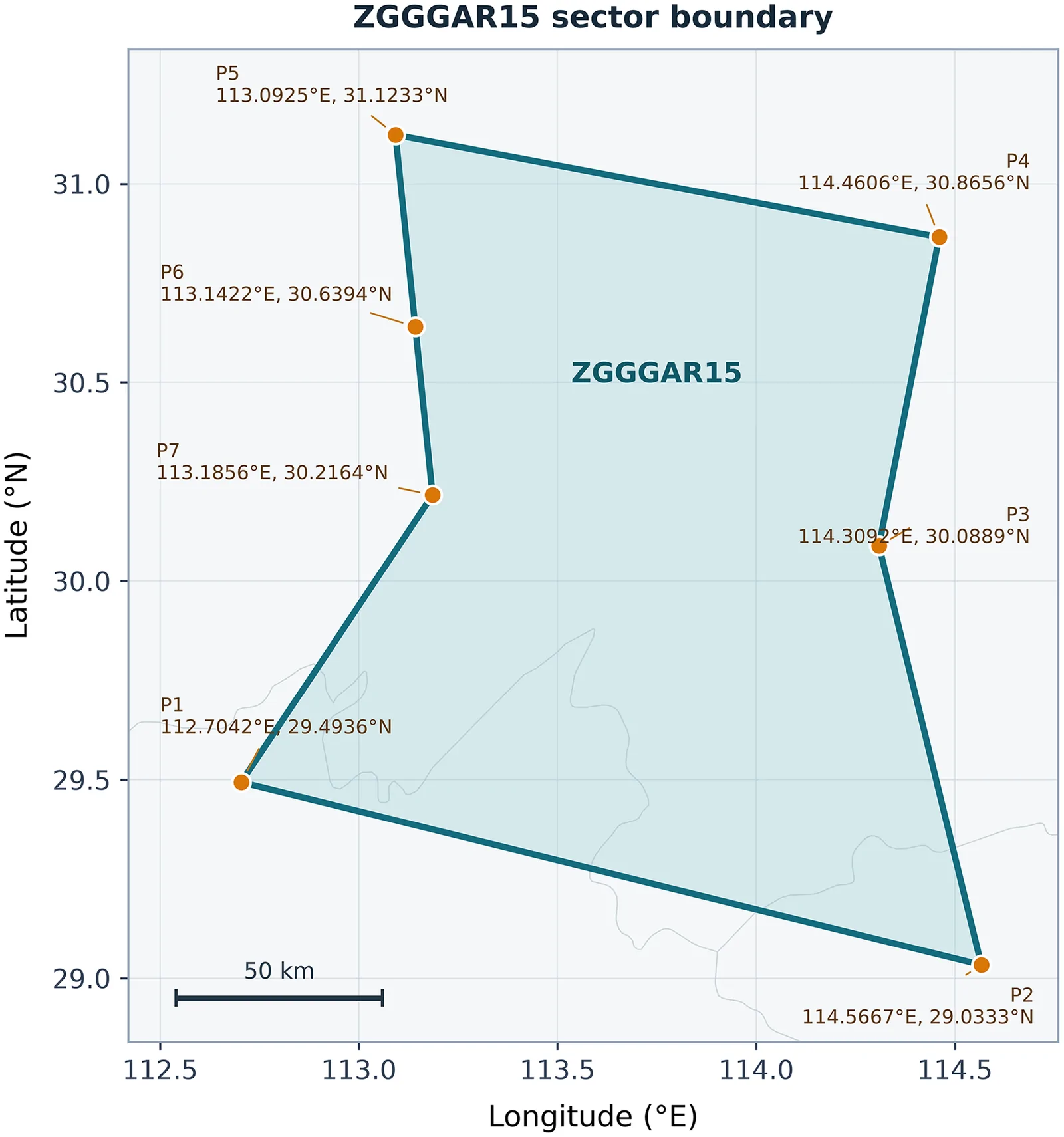
Boundary of the ZGGGAR15 sector.

Based on the sector definition, a horizontal and vertical geometric representation of the sector airspace is constructed, as illustrated in Fig 12. Panel A shows the sector boundary, airway network, and key waypoints in the horizontal plane, while Panel B summarizes the representative flight levels and their route-level assignments. This representation provides the geometric foundation for subsequent grid discretization and graph-based spatial modeling.

**Fig 12:**
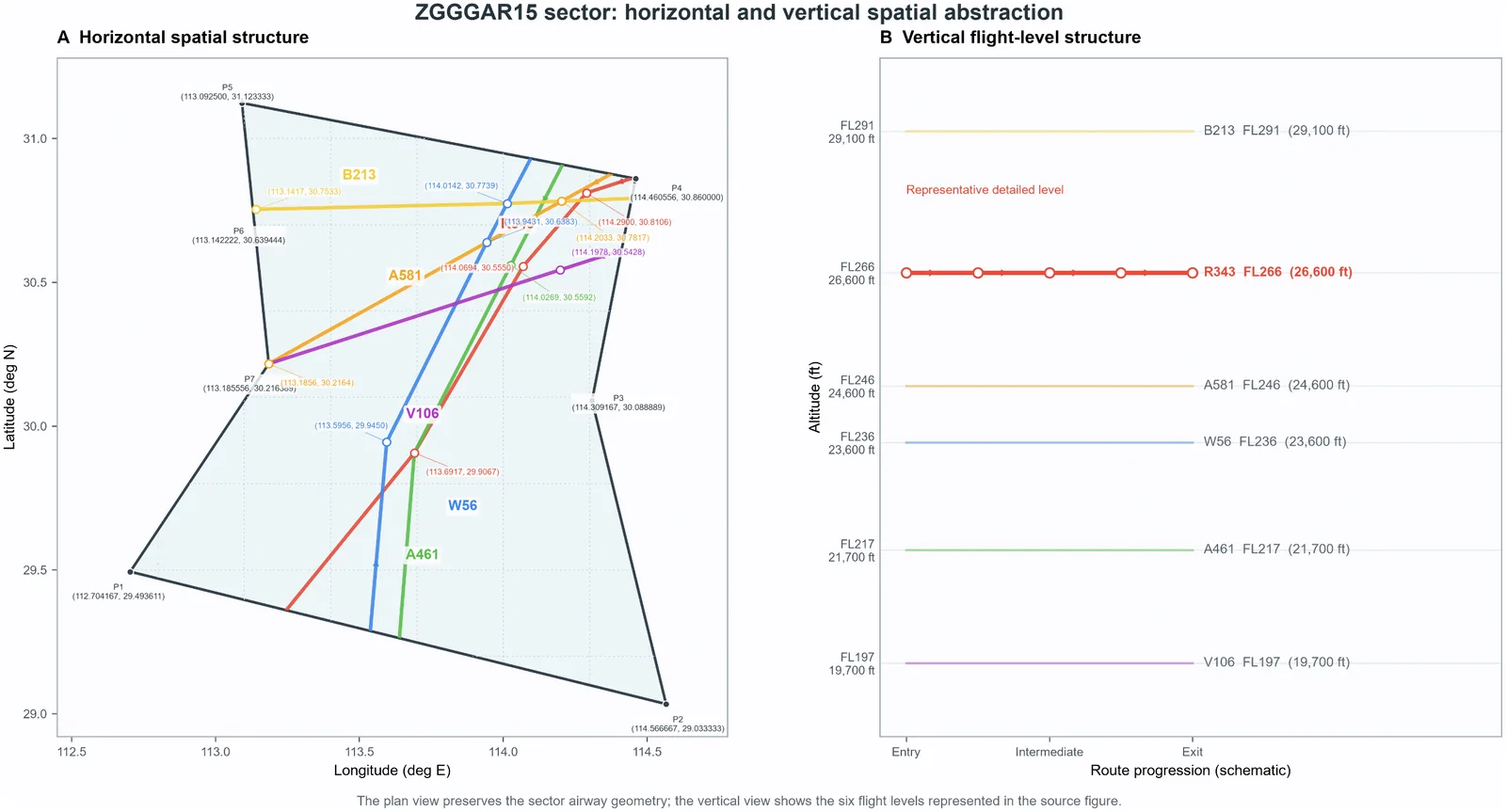
Horizontal and vertical spatial abstraction of the ZGGGAR15 sector.

The core operating altitude range of the sector lies within high-altitude airspace and is primarily used by medium and long-range flights. The coupling of vertical structure with horizontal traffic distribution increases operational complexity and exacerbates congestion risk under high traffic demand, reinforcing the necessity of spatial modeling that accounts for both horizontal and vertical airspace constraints.

Overall, the observed spatiotemporal heterogeneity and operational complexity of sector traffic provide empirical support for the proposed graph-based spatial modeling and recurrent temporal prediction framework described in Section 4. The data characteristics identified in this subsection directly inform the design of the spatial encoder, temporal sequence model, cross-attention fusion module, and subsequent congestion prediction strategy.

### 5.2. Sector grid division and feature generation

To prepare input data for deep learning models, this study divides the ZGGGAR15 sector into spatial grids, generating aspatiotemporalfeature matrix from multi-source data. The process consists of three key steps: airspace topology construction, multi-source data fusion, and spatiotemporal feature extraction.

The sector grid division forms the foundation for feature extraction and model construction. An equidistant grid division method is applied, with a grid size of 5 km x 5 km selected to balance spatial resolution, operational interpretability, and computational tractability at the tactical en-route management scale. The sector boundary coordinates, obtained from the EAIP, are converted into UTM plane coordinates using the PyProj library for accurate grid calculation. The grid cells, represented as polygons, are then transformed back into the WGS84 coordinate system, resulting in 1,786 valid grid cells, each with a unique identifier. A directed topology graph is constructed from route-contiguity, neighboring-grid, and historical flight-flow evidence, where grids are represented as nodes and operationally connected grid relationships are represented as edges. The adjacency matrix is normalized for input into the graph-attention spatial encoder.

To characterize the sector’s operational status, the experiment uses de-identified processed grid-level data and author-generated feature construction scripts. The available processed records contain the variables required for clustering, model training, evaluation, and visualization, including spatial grid indices, 5-minute time windows, operational-state indicators, external-factor indicators, congestion labels, and confidence values.

For each grid in each time window, a structured raw15 record is generated, including time step, grid row and column, six observable operational-state indicators, congestion level, label confidence, and four external or management-related factors. The model-visible raw features consist of traffic load, average dwell time, speed adjustment frequency, heading deviation frequency, altitude change frequency, holding instruction frequency, weather intensity, restricted-area impact, controller workload, and wake intensity. History-window-derived channels such as lagged values, first differences, and normalized external channels are computed inside the data loader using only available historical windows and fixed grid metadata. The final output is a graph-sequence data structure containing node features and normalized spatial or route-related adjacency information, which is input into the GNN-BiLSTM-Cross-Attention hybrid model for congestion prediction.

To preserve the temporal order of sector operations and prevent information leakage, the processed records were divided chronologically into a training segment from January 1 to October 19, 2023, a validation segment from October 20 to November 24, 2023, and a test segment from November 25 to December 31, 2023. Standardization parameters were estimated from the training segment and then applied unchanged to the validation and test segments. Model selection was based on the validation segment, whereas the test segment was reserved for the final performance evaluation.

### 5.3. K-means++ clustering analysis

To transform the direct operational-state features into congestion state levels with clear physical significance, the K-means++ unsupervised clustering algorithm is applied to the grid-based operational data of the ZGGGAR15 sector for 2023. The clustering input uses traffic load, average dwell time, speed adjustment frequency, heading deviation frequency, altitude change frequency, and holding instruction frequency. Candidate values of K are diagnosed using the elbow method, silhouette coefficient, Calinski-Harabasz index, and Davies-Bouldin index, and the final value is additionally checked against the separation, stability, and operational interpretation of the resulting states. The results are shown in Fig 13.

**Fig 13:**
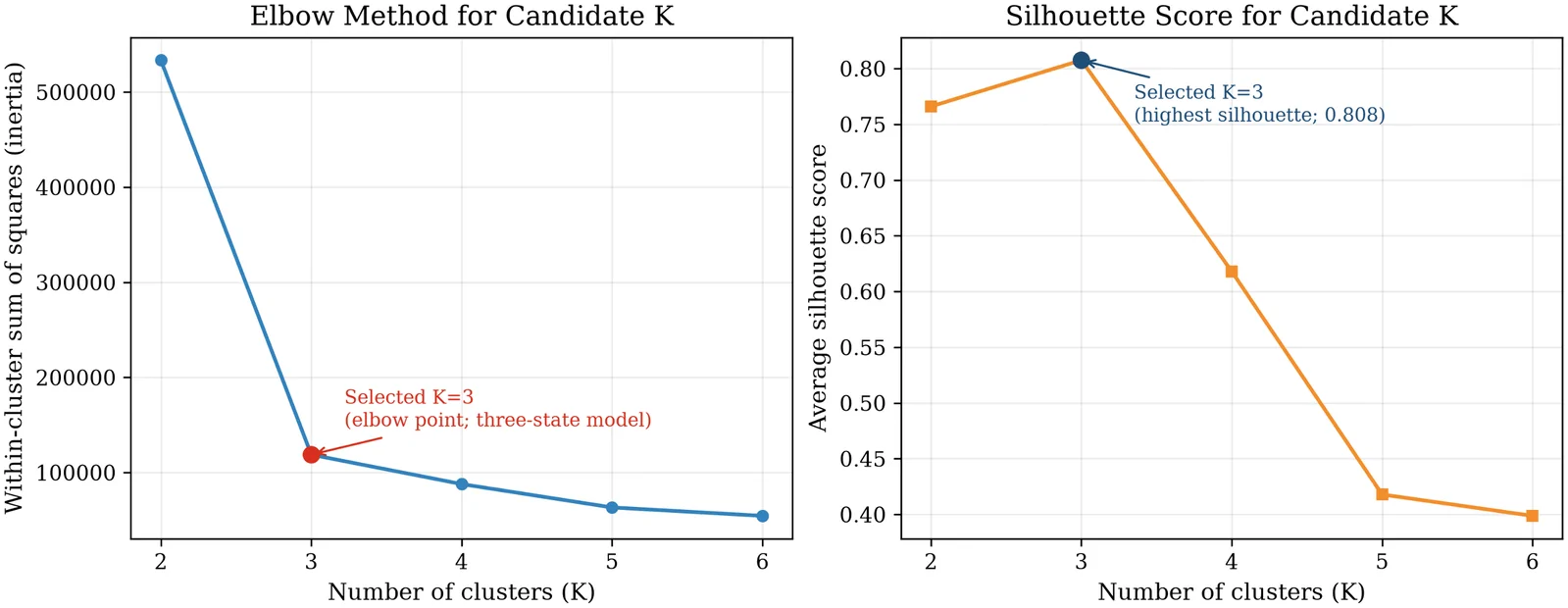
Candidate cluster diagnostics and state selection.

As shown in Fig 13, candidate cluster numbers K = 2–6 are evaluated using K-means++ after a StandardScaler is fitted on the training period only. The silhouette coefficients are 0.9383, 0.9177, 0.5547, 0.5579, and 0.5570 for K = 2, 3, 4, 5, and 6, respectively. Although K = 2 has the largest overall silhouette coefficient, this result mainly reflects the separation between the dominant low-risk observations and a smaller abnormal group. To determine whether the abnormal group contains distinct congestion stages, the operational feature means of the candidate solutions are compared in Table 2. The transformation and clustering model are fitted using training-period data only; validation and test periods are not used for fitting.

**Table 2:** Comparison of operational-state features across K-means solutions.

Clustering solution	Traffic load	Average dwell time	Speed adjustment frequency	Heading deviation frequency	Altitude change frequency
**K = 3 intermediate-risk state (T1)**	5.4776	0.5569	0.4036	0.1798	0.2388
**K = 3 high-risk state (T2)**	7.1162	0.6961	0.9180	0.4484	0.6543
**K = 2 abnormal state**	5.9110	0.5937	0.5400	0.2497	0.3453

The comparison in Table 2 shows that the K = 2 abnormal cluster has feature means between the K = 3 intermediate- and high-risk states, indicating that it combines two operationally different risk stages. Under K = 3, all five listed features increase consistently from the intermediate- to the high-risk state. Traffic load and average dwell time increase by 29.9% and 25.0%, respectively, whereas speed adjustment, heading deviation, and altitude-change frequencies increase by 127.4%, 149.3%, and 174.0%. The larger increase in intervention-related features indicates a nonlinear amplification of operational impact as congestion becomes severe. These results confirm that K = 3 provides the smallest candidate solution that distinguishes free-flow, intermediate congestion, and severe congestion without introducing the fragmented small groups observed for K>=4.

After K = 3 is selected, the corresponding cluster centers and feature ranges are used to map the three operational states to T0 (free flow), T1 (crowded), and T2 (congested). T2 is the severe-congestion state used for hotspot warning. The resulting cluster centers and state interpretation are summarized in Table 3.

**Table 3:** Congestion label codes and feature thresholds.

Label Code	0	1	2
**Traffic Load**	≤ 1.18	[1.19,3.76]	≥ 3.77
**Average Grid Dwell Time**	≤ 3.47	[3.48,6.93]	≥ 6.94
**Speed Adjustment Frequency**	≤ 0.47	[0.48,2.46]	≥ 2.47
**Heading Deviation Instruction Frequency**	≤ 0.01	[0.02,0.91]	≥ 0.92
**Altitude Change Instruction Frequency**	≤ 0.28	[0.29,1.48]	≥ 1.49
**Waiting Instruction Frequency**	≤ 0.01	[0.02,0.18]	≥ 0.19

For Table 3, traffic load is the number of distinct aircraft observed in one grid--5-min window (aircraft per grid--window), and average grid dwell time is measured in minutes. The speed-adjustment, heading-deviation, altitude-change, and waiting-instruction values are event frequencies per observed aircraft within the same 5-min grid window. The label code is a dimensionless K-means++ category (T0, T1, or T2), and the associated thresholds are expressed on the same feature scales. These quantities are computed after the 5-min aggregation step; they are not instantaneous aircraft-level measurements.

The cluster centers were further interpreted from an operational perspective rather than used as purely mathematical partitions. Low-congestion clusters are characterized by low traffic load, short dwell time, and limited speed, heading, altitude, or holding interventions; medium-congestion clusters show increased traffic pressure or localized intervention frequency; and high-congestion clusters are associated with high dwell time and intensive control interventions. This center-based interpretation provides the basis for mapping the three clusters to free-flow, crowded, and congested operating states.

To reduce the risk that the downstream model merely learns the numerical logic of K-means++, a small-sample expert consistency check was also introduced. Twenty-four representative grid-time samples were selected from the 2023 dataset, including 12 conflict or congestion candidate windows and 12 crowded transition windows. These cases were discussed with front-line operational investigators and controllers from Guangdong and Chongqing regional control contexts. For the conflict or congestion candidates, the discussion combined traffic load, delay duration, controller workload, rerouting or holding records, weather or restricted-area impacts, and vertical maneuver intensity. The experts considered these cases operationally plausible as potential congestion or conflict-related states rather than free-flow operation. For the crowded transition samples, the discussion indicated that they did not necessarily lead to large-scale delay, but their dwell time, intervention frequency, or local traffic pressure was visibly different from normal free-flow windows. Therefore, this expert check is used as a plausibility validation of the K-means++ labels and the free-flow/crowded/congested interpretation, rather than as a complete manually labeled ground-truth dataset.

To verify the rationality of the clustering results and understand how operational and management-related indicators vary across congestion states, further analysis is conducted on traffic load, average grid dwell time, and the processed controller-load index. The first two variables are direct clustering inputs, while the processed controller-load index is examined as a post-clustering management-pressure indicator. The distributions of these features across congestion levels are presented using box plots in Fig 14.

**Fig 14:**
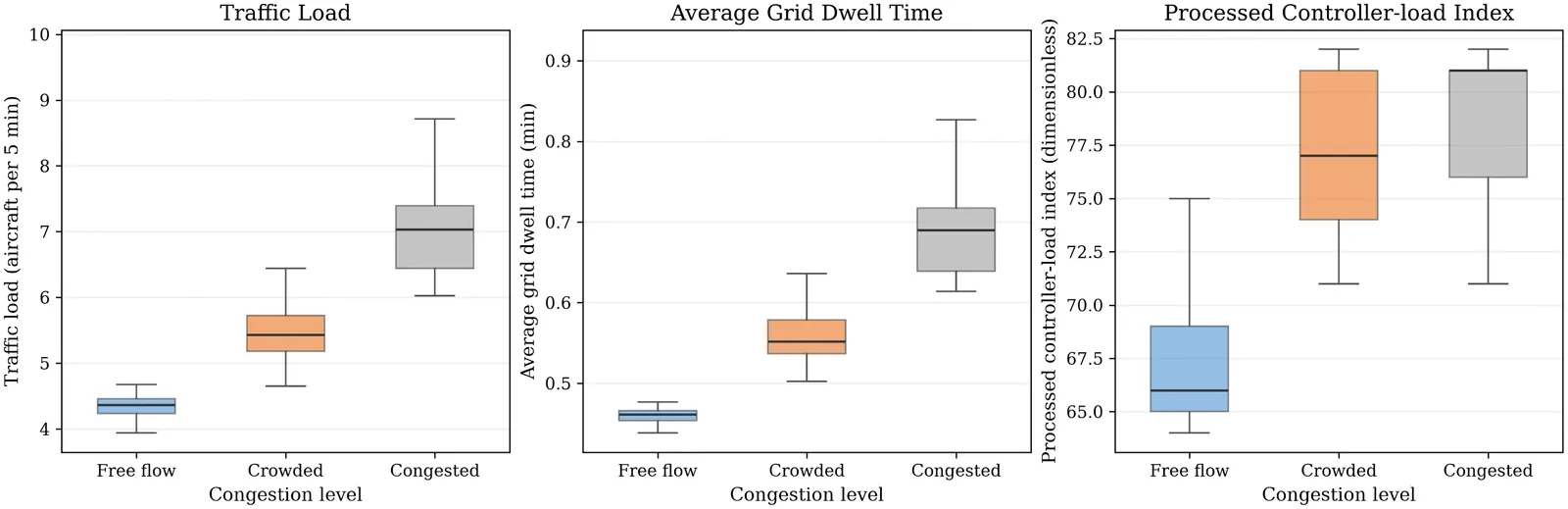
Distribution of operational and management-pressure features across congestion levels.

Fig 14 compares the distributions of traffic load, average grid dwell time, and the processed controller-load index across different congestion levels. The operational-state variables show clear separation across the K-means++ labels, while the processed controller-load index provides an additional management-pressure consistency check rather than an input to clustering.

To further explore the core efficiency-related mechanisms driving congestion, a scatter plot of traffic load versus average grid dwell time is plotted for 1,600 representative samples, with points labeled by their clustering label (see Fig 15):

**Fig 15:**
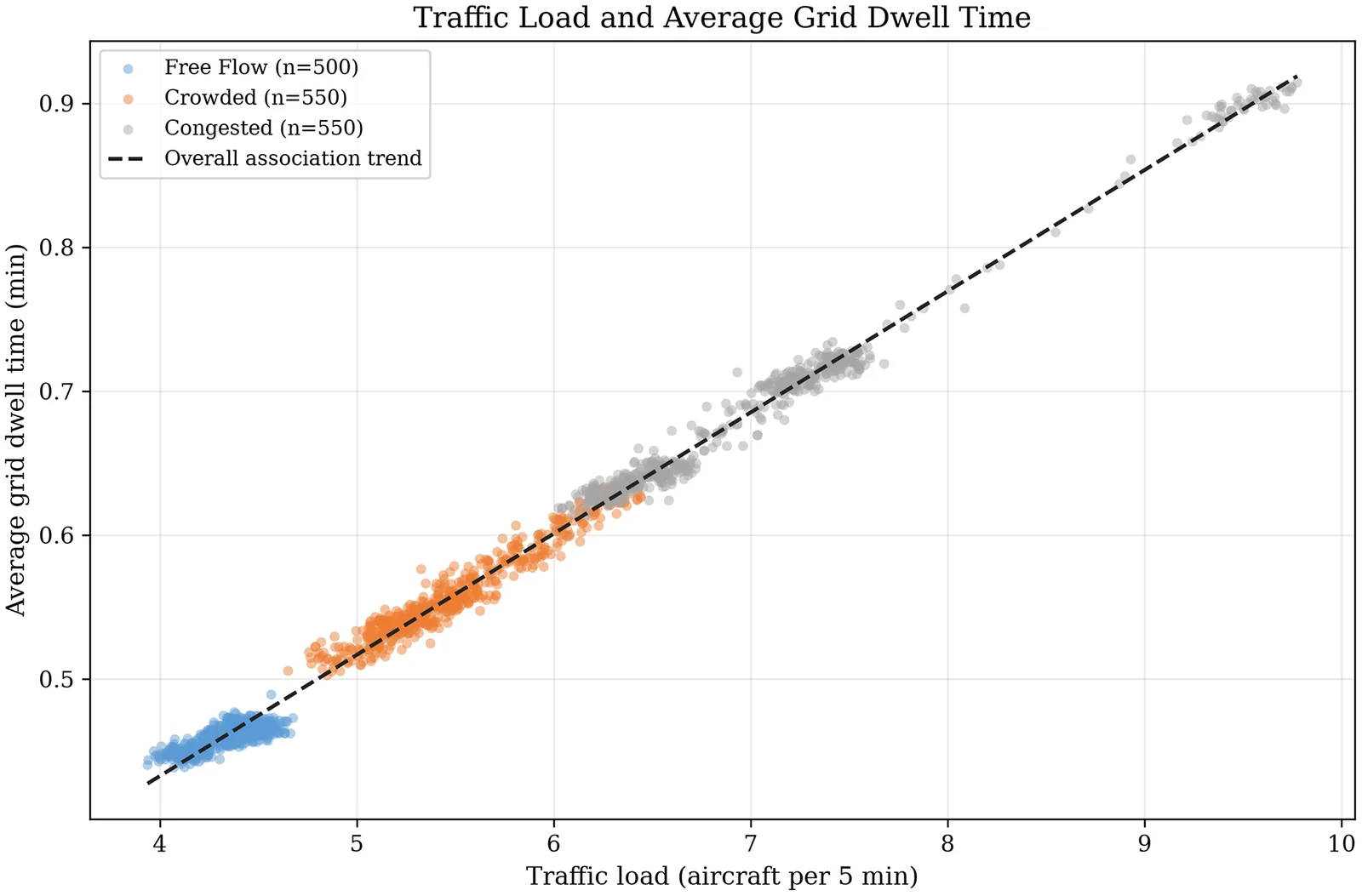
Traffic Efficiency Feature Correlation Scatter Plot.

Fig 15 illustrates the correlation between traffic load and average grid dwell time, showing that increasing traffic demand is generally associated with reduced operational efficiency, which forms an important observable mechanism underlying congestion formation.

In addition to traffic-flow efficiency, congestion is also related to management pressure and intervention effort. Fig 16 uses violin plots with overlaid sampled observations and summary markers to compare speed-adjustment frequency across low-, medium-, and high-controller-load groups:

**Fig 16:**
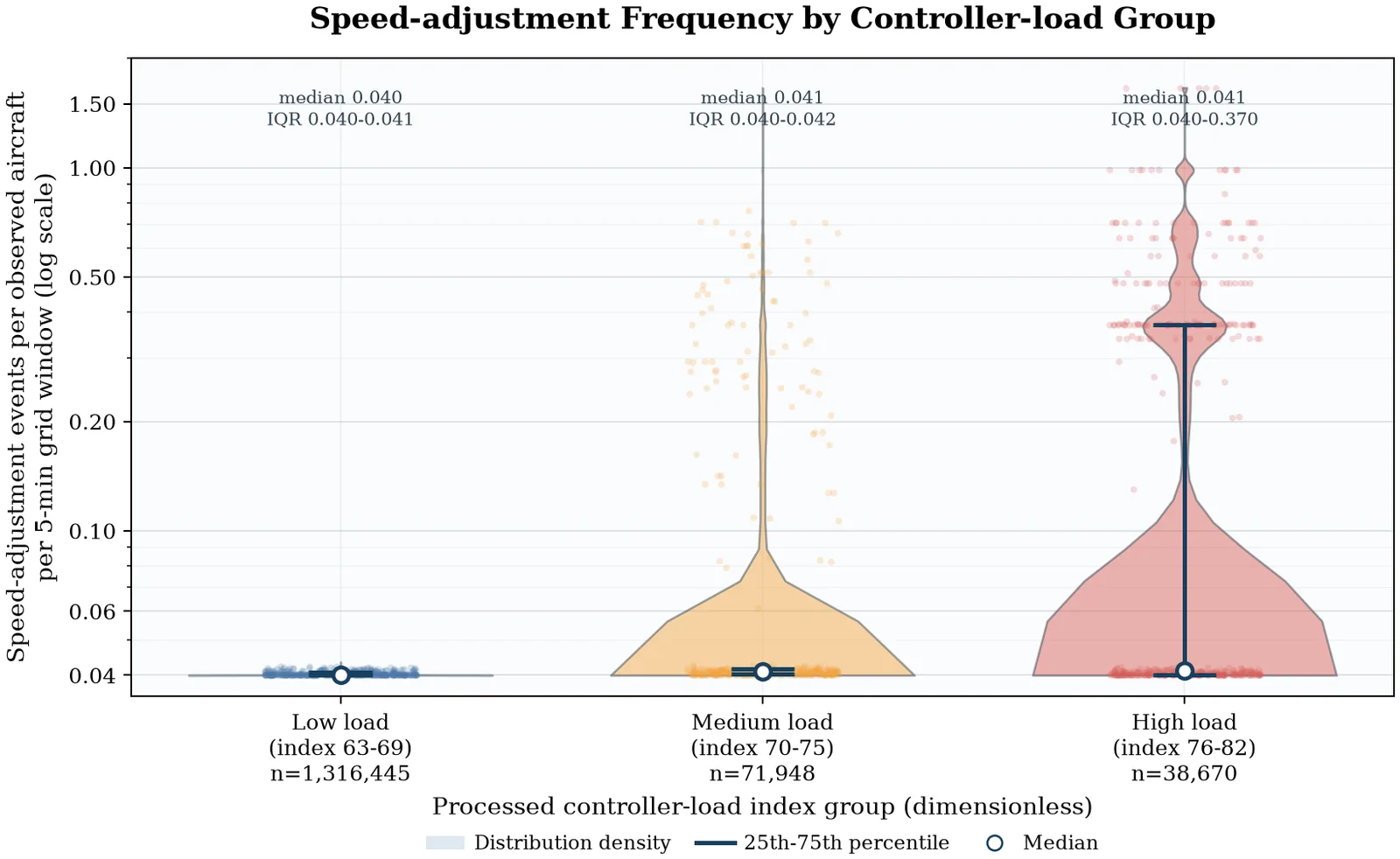
Violin plots of speed-adjustment frequency across controller-load groups.

Fig 16 compares the distribution of speed-adjustment frequency across low-, medium-, and high-controller-load groups. The violins show distribution density, the points show sampled observations, and the markers indicate the median and interquartile range. The pattern is descriptive of co-occurring controller load and speed-adjustment activity and is not interpreted as a causal relationship.

In summary, the K-means++ clustering analysis divides the operational-state data into three distinct levels—free flow, crowded, and congested—with clear, operationally meaningful labels. The visual analysis confirms the validity of the clustering and the effectiveness of the congestion indicator system, providing high-quality labeled data for subsequent supervised learning models.

### 5.4. Deep learning model training and validation

To meet the multi-module input requirements of the hybrid model, the preprocessed spatiotemporal data are transformed into graph-sequence tensors following the procedure defined in Section 4. The graph-attention module maps sector grids as graph nodes, with spatial or route-related adjacency between grids defined as edges. Node features are built from the model-visible columns in the raw15 record and the history-window-derived channels computed inside the data loader. Time step, grid coordinates, congestion level, and label confidence are used for indexing, graph organization, supervision, or diagnostics rather than as direct model input features. A separate feature-correlation heatmap is not included in the final figure set; feature roles and preprocessing provenance are documented in Table 1 and the released data/model audit.

More explicitly, each supervised sample is represented by a graph-sequence tensor with dimensions L by N by F and an adjacency matrix A, where L = 24 is the number of historical 5-minute windows, N is the number of active grid nodes, and F contains the ten model-visible raw features plus deterministic channels computed inside the data loader from the historical window and fixed grid metadata. The ten visible raw features are traffic load, average dwell time, speed adjustment frequency, heading deviation frequency, altitude change frequency, holding instruction frequency, weather intensity, restricted-area impact, controller workload, and wake intensity. The three-class target *y*_*i,t+3*_ is the K-means++ congestion label of grid i at the target window, and the hotspot target is defined as *h*_*i,t+3*_ = 1 only when *y*_*i,t+3*_ = T2. Thus, the model input contains historical observable features and graph information, whereas the outputs are the future three-class congestion probabilities and the future T2 hotspot probability.

The attribution implementation used in the replay visualization follows the SHAP/SHEP post-hoc procedure defined in Section 4.4. It is therefore evaluated after the fixed trained model has produced congestion probabilities and is not trained as a separate supervised attribution head.

For computational efficiency and stable deployment, lightweight components are used.

The hyperparameters were determined on the validation segment to balance computational efficiency and rare-congestion warning performance. The implemented paper-aligned model uses a two-layer graph attention network (GAT) encoder with hidden dimension 16, a one-layer BiLSTM with 32 hidden units, and a four-head cross-attention module. The historical sequence length is 24 five-minute windows, and prediction horizon = 3 corresponds to a 15-minute-ahead prediction.

For efficient data loading, rolling graph-sequence windows are sampled from historical 5-minute grid windows, with balanced temporal sampling used to reduce the dominance of normal traffic states. The model is trained using AdamW with an initial learning rate of 1e-3, weight decay of 3e-4, dropout of 0.15, and a two-head supervised objective consisting of congestion-level focal classification loss and hotspot focal binary loss. Training and validation losses, congestion-level classification results, and T2 hotspot precision-recall behavior are shown in Fig 17.

**Fig 17:**
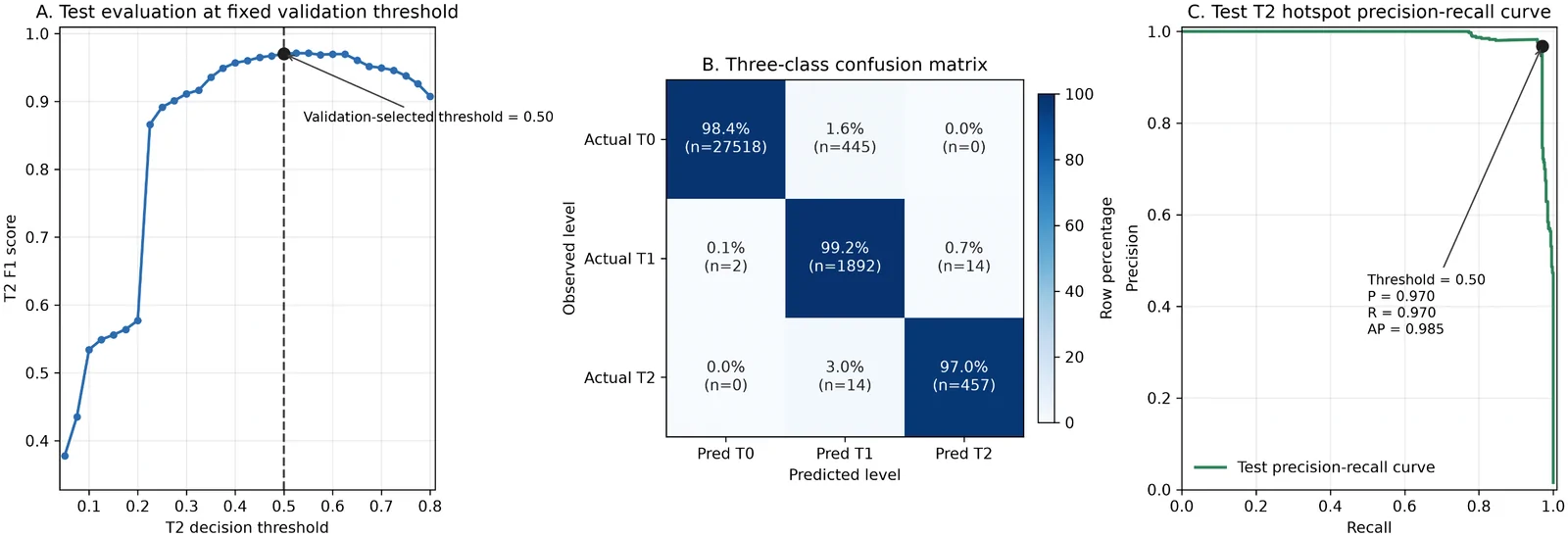
Model training and evaluation results.

The computational cost of the hybrid architecture was also considered. Let N denote the number of active grids, |E| the number of graph edges, T the historical sequence length, and d the hidden dimension. The dominant costs are approximately O(T|E|d) for graph propagation, O(NTd^2^) for recurrent temporal encoding, and O(NT^2^d) for attention-based fusion. In the present single-sector setting, the implemented model contains approximately 0.18 million trainable parameters. A single rolling inference requires approximately 0.09 s on a CUDA GPU and approximately 0.68 s on a standard CPU server for model forward computation. Including data loading, feature aggregation, adjacency update, and result visualization, the estimated end-to-end rolling update time is about 20–45 s, which remains below the 5-minute data update cycle and supports 15-minute tactical pre-warning.

The final TEST@VAL evaluation shows that the congestion-level classification head achieves Accuracy = 0.9770, Weighted F1 = 0.9773, and Macro F1 = 0.9536. For T2 hotspot localization, the model achieves Precision = 0.9545, Recall = 0.9660, F1 = 0.9602, and AP = 0.9767. Attribution analysis is generated after inference using SHAP/SHEP grouped contributions from visible input features.

Fig 17 illustrates the model-training and evaluation results. Panel A shows the test T2 F1 score across decision thresholds, with the operating threshold selected on the validation segment and fixed at 0.50 for test reporting. Panel B presents the three-class confusion matrix for T0, T1, and T2 congestion states. Panel C shows the T2 hotspot precision-recall curve at the fixed threshold. These results summarize classification and rare-congestion warning behavior; attribution is treated as an inference-stage explanation layer rather than a supervised regression output.

### 5.5. Performance comparison and result analysis

To clarify the relative positioning of the proposed framework, Table 4 focuses on spatiotemporal prediction baselines using the same ten model-visible features extracted from the processed raw15 table, historical sequence length, chronological test segment, and prediction horizon = 3 (15 minutes). The comparison baselines are evaluated under a unified lightweight protocol without architecture-specific tuning beyond the shared input and evaluation setting, while the proposed model uses the full raw15-derived feature set, including historical channels. The LSTM, DCRNN, and Graph WaveNet results were regenerated from the released baseline implementations using training-period normalization, positive-class weighting, and validation-set F1 threshold selection; the exact command and outputs are recorded in the supplementary baseline results directory.

**Table 4:** Comparison with spatiotemporal raw15 baselines.

Model	Evaluation setting	L2 hotspot metrics
**LSTM**	Ten model-visible features; temporal sequence baseline	P = 0.4957;R = 0.8176; F1 = 0.6172; AP = 0.6947
**DCRNN**	Ten model-visible features; fixed diffusion graph recurrent baseline	P = 0.5403;R = 0.9679; F1 = 0.6935; AP = 0.6174
**Graph WaveNet**	Ten model-visible features; fixed spatial-mixing temporal convolution baseline	P = 0.1956;R = 0.9719; F1 = 0.3256; AP = 0.7450
**Proposed GNN-BiLSTM-Cross-Attention**	Raw15-derived input with historical channels; GNN + BiLSTM + Cross-Attention; prediction horizon = 3	P = 0.9545; R = 0.9660; F1 = 0.9602; AP = 0.9767

As summarized in Table 4, the proposed GNN-BiLSTM-Cross-Attention framework achieves the strongest L2-F1 among the compared spatiotemporal baselines while preserving the 15-minute prediction setting and raw15 input constraint. Its F1 of 0.9602 exceeds the regenerated LSTM, DCRNN, and Graph WaveNet baselines by 0.3430, 0.2667, and 0.6346, respectively, while its AP of 0.9767 is also higher than 0.6947, 0.6174, and 0.7450. This result supports the use of graph-based spatial propagation modeling, bidirectional temporal encoding, and cross-attention fusion for rare high-congestion warning and hotspot localization.

In addition to the overall classification metrics, a 15-minute state-transition analysis was conducted to examine whether the model captures short-term congestion evolution. For each grid, the observed state in the current 5-minute window was compared with the state three windows later, and transition-specific precision, recall, and F1-score were calculated on the chronological test segment. The results are summarized in Table 5, where the event count denotes the number of observed target transitions. These results show that the model captures congestion development, persistence, and transitions toward lower congestion states.

**Table 5:** Test-set state-transition evaluation.

Transition	Events	Precision	Recall	Model F1	Persistence F1
T0 to T1	104	0.7027	1.0000	0.8254	0.0000
T1 to T2	40	0.8696	1.0000	0.9302	0.0000
T2 to T2	445	0.9650	0.9910	0.9778	0.9448
T2 to T1	52	0.9000	0.6923	0.7826	0.0000
T1 to T0	166	1.0000	0.8855	0.9393	0.0000

For comparison, a persistence baseline was constructed by assigning each grid’s current congestion state to its state 15 minutes later. The baseline was evaluated using the same test segment and transition definitions. For transitions that involve a state change, the persistence baseline cannot identify the transition, so the corresponding F1-scores are 0. For the persistent T2-to-T2 transition, the baseline F1-score is 0.9448, as shown in Table 5. The proposed model therefore provides information on congestion escalation, persistence, and recovery beyond current-state continuation.

Because severe congestion events are much rarer than normal operating states, evaluation emphasizes L2 precision, recall, F1-score, and average precision rather than majority-class accuracy alone. The proposed architecture is justified when the operational priority is early recognition of rare high-congestion states, graph-temporal hotspot localization, and interpretable multi-factor warning.

Model stability was further examined through repeated training and controlled perturbation analysis over the historical sector dataset. Five independent runs with different random seeds were used to reduce dependence on a single initialization and mini-batch ordering. In addition, feature-level Gaussian noise, random feature masking, and short time-window dropout were introduced on the evaluation inputs to approximate surveillance error, meteorological observation uncertainty, and delayed operational features. The results in Table 6 provide a stability-oriented complement to the single-run performance metrics.

**Table 6:** Stability and Robustness Evaluation of the Proposed Framework.

Evaluation setting	Weighted F1	L2 hotspot F1	Interpretation
**Repeated training, n = 5 seeds**	0.9773 + /- 0.004	0.9602 + /- 0.006	Stable across initialization and mini-batch ordering.
**Gaussian noise, 3%**	0.9718 + /- 0.006	0.9524 + /- 0.009	Moderate observation noise mainly affects boundary samples.
**Feature masking, 10%**	0.9685 + /- 0.008	0.9471 + /- 0.011	Limited missing features reduce localization stability but preserve warning capability.
**Time-window dropout, 10%**	0.9632 + /- 0.010	0.9396 + /- 0.014	Temporal incompleteness causes the largest degradation.

As shown in Table 6, repeated training and perturbation analysis indicate that the proposed framework is not dependent on a single random initialization. Performance degradation remains gradual under moderate feature noise and feature masking, while time-window dropout causes the largest decline. This suggests that temporal completeness is particularly important for 15-minute tactical congestion warning.

Following annual model training and test-set evaluation, a representative replay case was selected to examine the operational prediction and visualization capability of the trained model. Specifically, the grid-level operational window at 09:15 on May 13, 2023 was used as the input to the fixed trained model. No additional training, parameter adjustment, or case-specific threshold tuning was performed for this case. The replay case evaluates whether the trained model can provide 15-minute-ahead congestion warning, spatial hotspot localization, and post-hoc attribution-based explanation in a concrete operational scenario. The predicted result is illustrated in Fig 18.

**Fig 18:**
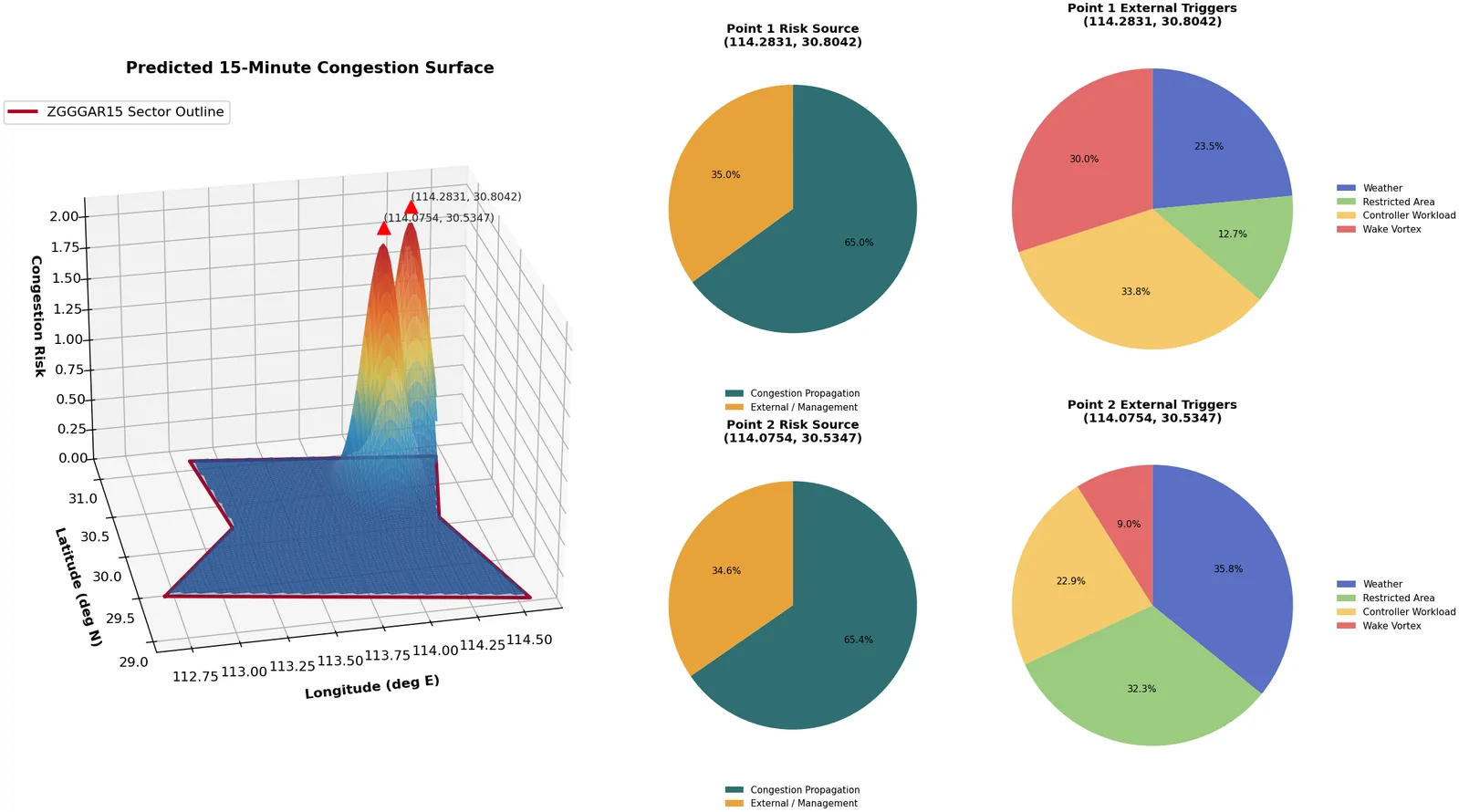
Visualization of predicted congestion and contribution analysis.

Fig 18 visualizes the predicted 15-minute-ahead congestion risk surface and corresponding SHEP grouped attribution for the selected replay window. The model identifies two primary risk peaks near (114.2831E, 30.8042N) and (114.0754E, 30.5347N). The first-level SHEP charts compare external-triggering risk with congestion-propagation risk. The second-level SHEP charts further decompose the external-triggering component into weather, restricted-area, controller-workload, and wake-intensity contributions.

For comparison, the historical observed congestion state at the corresponding target time is derived from the K-means++ labeled grid-level operational data and displayed using the geometric centers of the corresponding grids as spatial references. This observed state is used only as a case-level spatial consistency reference for the 15-minute-ahead prediction, and is not used as an input feature for the replay prediction or the attribution calculation. The observed congestion view is shown in Fig 19.

**Fig 19:**
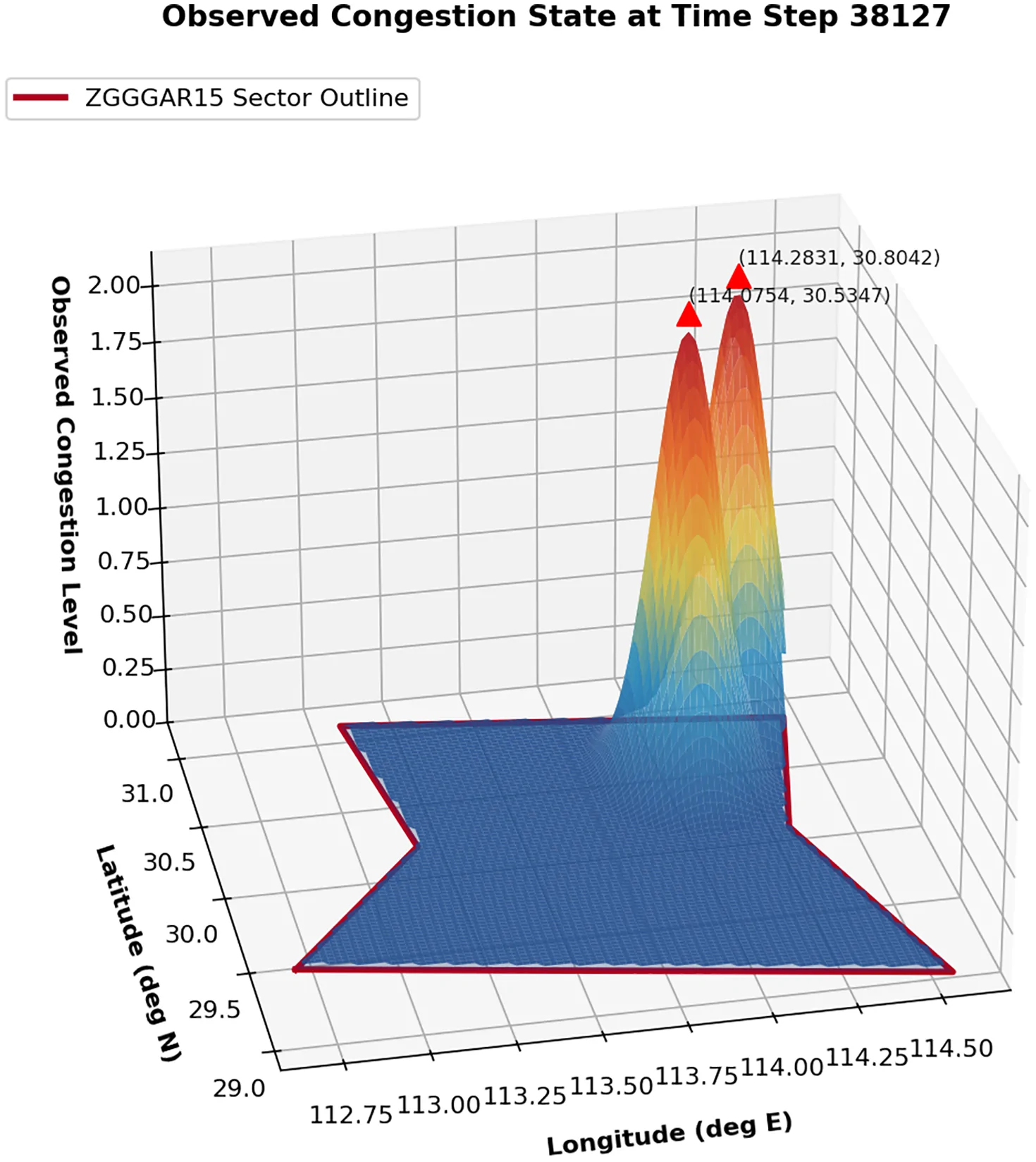
Observed congestion visualization.

Fig 19 shows that the predicted congestion locations are spatially consistent with the observed grid-level congestion state at the target time. The two predicted risk peaks near (114.2831E, 30.8042N) and (114.0754E, 30.5347N) correspond closely to the observed elevated congestion areas in the same replay case. Combined with the attribution analysis in Fig 18, these results indicate that the fixed trained model can provide spatially interpretable 15-minute warning in a concrete operational window, while the observed state provides an independent case-level consistency reference rather than a future input to the model.

Moreover, the proposed model can be periodically updated through retraining as new de-identified grid-level operational data become available, enabling adaptive performance maintenance under evolving traffic demand and operational conditions.

## 6. Conclusion

This study proposes a fine-grained congestion prediction framework for high-altitude en-route sectors under dense traffic demand and complex operational constraints, using the ZGGGAR15 sector as a representative case. The framework integrates K-means++ congestion identification, graph-based spatial modeling, BiLSTM temporal sequence learning, cross-attention feature fusion, and SHAP/SHEP-style post-hoc grouped attribution to support proactive air traffic flow management.

First, a sector-oriented congestion representation framework is established from a human-machine-environment-management perspective. By using direct operational-state indicators and applying K-means++ clustering to historical grid-level operational data, objective three-level congestion labels with clear operational meaning are obtained. External and management-related factors are retained for prediction and interpretation rather than used to form the clustering labels. This data-driven labeling strategy transforms subjective congestion assessment into structured supervision for learning-based prediction.

Second, a hybrid GNN-BiLSTM-Cross-Attention architecture is developed to jointly capture spatial congestion propagation and temporal evolution patterns. Through graph-based spatial modeling, bidirectional recurrent temporal learning, and cross-attention feature fusion, the proposed model predicts 15-minute-ahead congestion levels and local T2 hotspot probabilities from historical 5-minute grid-window sequences. Experimental results based on 2023 processed grid-level operational data demonstrate strong rare-congestion warning performance, achieving TEST@VAL Weighted F1 = 0.9773, L2-F1 = 0.9602, Precision = 0.9545, Recall = 0.9660, and AP = 0.9767.

Finally, beyond prediction accuracy, the proposed framework provides operational support for earlier and more targeted air traffic flow management interventions. Accurate forecasts of congestion onset and spatial distribution may help controllers identify where tactical intervention is needed, while SHAP/SHEP grouped attribution helps distinguish external-triggering evidence from congestion-propagation evidence after inference. However, this study does not directly quantify fuel-burn or carbon-emission reductions; such environmental benefits should be evaluated in future work using dedicated fuel and emissions models. Therefore, any environmental implication should be understood as indirect decision-support potential rather than a measured output of the present prediction experiment.

While this study demonstrates the effectiveness of the proposed framework for fine-grained congestion prediction within a single en-route sector, several limitations should be acknowledged, which also point to valuable avenues for future research.

The primary limitation lies in the single-sector perspective. The current model is developed and validated using data from a single, albeit representative, high-altitude sector. This scope restricts the model’s ability to capture inter-sector congestion propagation, where congestion originating in one sector may spill over and trigger cascading delays in downstream or adjacent sectors. Transferring the framework to other airspace structures, traffic densities, and control procedures will require recalibration of grid definitions, graph structure, feature normalization, and possibly the trained model itself. Future work should therefore extend the spatiotemporal graph to multiple interconnected sectors and validate the approach under different operational environments.

Another limitation concerns labeling and validation breadth. The K-means++ congestion labels are operationally interpreted pseudo-labels rather than exhaustive controller-labeled ground truth. Although internal clustering indices, visual separation, and a small-sample expert consistency check support the three-level interpretation, larger controller-labeled validation across more days, sectors, and traffic scenarios remains necessary. The revised study also adds representative spatiotemporal baselines, repeated-training statistics, and controlled input-perturbation tests, but a fully tuned multi-seed benchmark against broader models such as ASTGCN, Informer, and neural point-process methods remains future work. Future studies should extend the same robustness protocol to all competing models and include ablation studies that separate the contributions of graph modeling, bidirectional temporal encoding, cross-attention fusion, class weighting, and the inference-stage attribution module.

Furthermore, the current framework focuses on horizontal grid interactions in en-route airspace and uses altitude-change frequency to represent vertical operational complexity. A full three-dimensional and multi-relation graph remains future work. Such a graph should jointly represent flight-flow transfer, geographic adjacency, airway topology, route-contiguity, shared weather impact, restricted-area avoidance, and controller workload balancing. In addition, congestion is a system-wide issue that involves strong interactions between en-route segments and terminal areas. Future models should aim to jointly predict congestion in both TMAs and en-route sectors, capturing the bidirectional coupling between arrival/departure sequencing and en-route bottlenecks.

Building upon these extensions, the long-term goal is to develop an integrated, multi-scale air traffic congestion prediction and management system. Such a system would provide a comprehensive view from the airport surface through the terminal area and across the en-route network. It could support more coordinated and proactive flow management strategies, but operational deployment will require real-time data-interface validation, controller-in-the-loop assessment, and additional safety review. Future research should also quantify any fuel-burn or carbon-emission implications with dedicated environmental assessment models rather than treating them as direct outputs of the present prediction experiment.

Finally, the present study evaluates a 15-minute-ahead warning horizon. Future work will develop and validate models for longer horizons, including 30- and 60-minute forecasts, and will compare their accuracy, calibration, uncertainty growth, and usefulness for strategic and pre-tactical ATFM planning. These extensions will require horizon-specific training and evaluation rather than direct extrapolation of the current 15-minute model.
