# Supplementary material for: An operational spatiotemporal prediction framework for sector congestion in air traffic management
Source: PLoS One. 2026 Oct 27;21(10):e0355927. doi: 10.1371/journal.pone.0355927 (PMC13712534; doi:10.1371/journal.pone.0355927)
Supplement: Supplementary file 1 [file pone.0355927.s002.pdf]

Review of Manuscript ID: PONE-D-26-08794R1
An operational spatiotemporal prediction framework for sector congestion in air traffic management

Summary

This manuscript presents a 15-minute-ahead framework for en-route sector congestion prediction based on grid-level operational data. The approach combines K-means++ pseudo-labeling of congestion states with a GNN-BiLSTM-Cross-Attention architecture for three-class congestion prediction and T2 hotspot warning, followed by post-hoc grouped attribution for operational interpretation. The study is evaluated on 2023 data from the ZGGGAR15 high-altitude en-route sector and reports strong predictive performance, together with comparisons against temporal and graph-temporal baselines, robustness analyses, and computational-feasibility estimates. The topic is relevant to air traffic flow management, and the overall methodological framework is clearly motivated and potentially useful for short-term tactical decision support.

General Summary

Overall, the manuscript is within the scope of PLOS ONE and provides a technically coherent and reproducible application of spatiotemporal learning to sector-congestion warning. I recommend Revision. The main points that still require attention concern the transparency of the temporal evaluation protocol and the interpretation of the very high short-horizon predictive performance. In particular, the manuscript should make the train/validation/test separation fully explicit and provide a simple check showing that the reported 15-minute-ahead performance is not driven predominantly by persistence of the current congestion state. In addition, a small moderation of the strongest operational claims would better reflect the present single-sector retrospective validation. These are focused revisions and do not require a redesign of the study.

Major Comments

- Temporal split and leakage control. The manuscript refers to a chronological test segment and states that the warning threshold is selected on the validation segment, but the exact temporal boundaries of the training, validation, and test partitions are not sufficiently explicit. Please report the corresponding date ranges (or an equivalently clear chronological definition) and state explicitly that all data-dependent preprocessing and model-selection steps relevant to the reported test performance are determined without using test-period information. This clarification is important because the model is built from rolling historical windows and the reported accuracy is very high.
- Distinguishing forecasting skill from short-term state persistence. The prediction horizon is 15 minutes, whereas the model uses a relatively long recent history and congestion states may remain stable over consecutive 5-minute windows. To make the predictive result easier to interpret, please add one simple persistence/last-state reference or, alternatively, report performance separately for transitions into T2 (e.g., T0/T1 to T2) and persistence of an already congested state (T2 to T2). A compact analysis of this type would provide direct evidence that the framework contributes to early warning rather than mainly reproducing short-term state continuity.
- Scope of the operational claim. The retrospective evaluation is based on one sector and one year of data, which is appropriate for a proof-of-concept study, but the Abstract currently describes the framework as providing “operationally usable” support. Please moderate this wording to emphasize potential operational applicability or decision-support value, and state clearly that validation across additional sectors and operational environments is still required before broader deployment claims can be made. No additional external dataset is essential for the present manuscript if the scope of the conclusions is stated proportionately.

Minor Comments

- Please standardize the terminology "spatiotemporal" versus "spatio-temporal" throughout the manuscript. Both forms are currently used; adopting one form consistently (preferably "spatiotemporal," consistent with the title) would improve editorial consistency.
- In Eq. (16), the output dimension is written as d_out, whereas the following sentence defines F_out as the output feature dimension. Please use a single symbol consistently and define it unambiguously.
- **Table 2:** Please report the **units of measurement** for the variables and thresholds presented in Table 2 (e.g., average grid dwell time and intervention frequencies). If any quantities are normalized or dimensionless, this should be stated explicitly to ensure unambiguous interpretation of the clustering boundaries.
