# Supplementary material for: An operational spatiotemporal prediction framework for sector congestion in air traffic management
Source: PLoS One. 2026 Oct 27;21(10):e0355927. doi: 10.1371/journal.pone.0355927 (PMC13712534; doi:10.1371/journal.pone.0355927)
Supplement: Supplementary file 2 [file pone.0355927.s001.docx]

Dear Dr. Jin and Reviewers,

We sincerely thank the Academic Editor and all reviewers for their careful evaluation of our manuscript and for the constructive comments. We also apologize for the long time taken to prepare this revised submission. Because the revision involved substantial methodological, experimental, reproducibility, visualization, and formatting changes, we took additional time to ensure that the manuscript, response letter, and submission materials were revised carefully and consistently. The comments have helped us improve the clarity, methodological transparency, experimental completeness, and reproducibility of the study. We have revised the manuscript and prepared this point-by-point response accordingly.

In the revised manuscript, we have strengthened the motivation for the GNN-BiLSTM-Cross-Attention architecture, expanded the related work, clarified the K-means++ labeling procedure and expert consistency check, and added representative spatiotemporal graph baselines. We also revised the original cause-factor prediction description: the model no longer treats cause weights as a supervised regression target; instead, congestion level and hotspot probability are predicted by the trained model, and SHAP/SHEP-style grouped attribution is computed after inference to explain external triggering and congestion-propagation contributions. In addition, we supplemented computational complexity and deployment analysis, added repeated-run and perturbation-based robustness evaluation, and revised the data availability approach so that the full-year de-identified processed grid-level data and all author-generated code required to reproduce the reported results are publicly available on Figshare with DOI: https://doi.org/10.6084/m9.figshare.32901059.

In the revised manuscript and response package, we have made a broad set of substantive and presentation-level changes. The model description has been aligned with the actual GNN-BiLSTM-Cross-Attention implementation; the former supervised cause-factor regression has been removed and replaced by post-hoc SHAP/SHEP attribution; the K-means++ pseudo-labeling procedure has been clarified and supported with candidate-K validation and expert plausibility checking; and the experimental section now includes graph-temporal baselines, repeated-run stability, perturbation robustness, computational-cost analysis, and an operational replay visualization. We also reorganized the reproducibility materials so that the processed full-year grid-level data, K-means++ validation code and model, training scripts, trained prediction model, and interface demonstration materials can be checked against the revised manuscript.

# Response to Journal Requirements

### Journal Requirement 1

**Comment:** Please ensure that the manuscript meets PLOS ONE style requirements, including file naming.

Response: We appreciate this instruction and treated it as part of the resubmission preparation rather than as a simple file-management matter. The submission package has been reorganized into clearly separated components, including the clean manuscript, the marked-up manuscript, the response letter, figure files, reproducibility materials, and interface demonstration materials. During this process, we checked table and figure callouts, corrected reference-list numbering, and made the manuscript files consistent with the PLOS ONE formatting expectations so that the editorial office can verify the revised materials more easily.

### Journal Requirement 2

**Comment:** Please ensure that the corresponding author has a verified ORCID iD in Editorial Manager.

Response: We have noted and followed this requirement. The corresponding author ORCID iD is indicated as verified in Editorial Manager, and the coauthors will be encouraged to complete ORCID verification before final acceptance if needed.

### Journal Requirement 3

**Comment:** Please follow PLOS ONE guidelines for sharing author-generated code.

Response: We agree that the code-sharing statement needed to be more explicit. The revision is based on a multi-stage computational workflow, including feature preparation, K-means++ labeling, graph construction, model training, baseline comparison, robustness testing, SHAP/SHEP attribution, and interface visualization. We have deposited the author-generated code and the corresponding de-identified grid-level data needed to reproduce the reported results on Figshare with DOI: https://doi.org/10.6084/m9.figshare.32901059. The package is organized so that readers can validate the K-means++ labels, reproduce model training from the raw15 grid-level dataset, and run the trained-model interface case without relying on private scripts.

### Journal Requirement 4

**Comment:** Funding information should not appear in the manuscript text.

Response: We have followed this instruction and removed funding information from the manuscript text. The updated funding statement will be provided through the online submission system and, where requested, in the resubmission letter rather than in the scientific text. The revised funding statement is: This work was supported by the Graduate Student Scientific Research Innovation Fund of Civil Aviation Flight University of China (26CAFUC10019), and the Science and Technology Projects of Xizang Autonomous Region, China (XZ202601ZY0091 and XZ202403ZY0014). The funders had no role in study design, data collection and analysis, decision to publish, or preparation of the manuscript. This keeps the manuscript consistent with PLOS ONE requirements while ensuring that the submitted funding metadata are complete and accurate.

### Journal Requirements 5-7

**Comment:** Please revise the data availability approach and deposit the minimal anonymized dataset necessary to replicate the findings in a stable public repository.

Response: We agree that reproducibility should not depend on data availability upon request. We have therefore revised the data-sharing plan and deposited the corresponding de-identified grid-level data used in the experiments, together with all author-generated code required for reproduction, on Figshare with DOI: https://doi.org/10.6084/m9.figshare.32901059. The public package includes the processed full-year feature table, K-means++ labels and validation files, graph/edge information, train/validation/test splits, configuration files, evaluation scripts, baseline scripts, robustness-test scripts, source data for reported figures, and the interface case input used for the operational visualization. With these materials, readers can independently rebuild the label generation, model training, evaluation, and visualization pipeline from the de-identified data released for this study.

### Journal Requirement 8

**Comment:** Please provide copies of the images for all figures included in the manuscript.

Response: We prepared the figures as separate upload-ready files while retaining figure captions and callouts in the manuscript. The figure package was checked against the revised text so that the production office can match each uploaded image with the corresponding figure number, caption, and discussion in the manuscript.

### Journal Requirement 9

**Comment:** Please ensure that Table 1 and Table 2 are referred to in the manuscript text.

Response: We checked the table sequence and agree that the manuscript needed clearer table callouts. The revised text now explicitly cites Table 1 when introducing the indicator system, Table 2 when reporting the K-means++ clustering results, Table 3 when comparing the proposed model with LSTM, DCRNN, and Graph WaveNet, and Table 4 when discussing repeated-run and perturbation robustness. This makes the flow from indicator construction, to pseudo-label validation, to model comparison, and finally to robustness testing easier to follow.

### Journal Requirement 10

**Comment:** Please include captions for Supporting Information files and update in-text citations accordingly.

Response: We have revised the manuscript to address this requirement. Because no additional Supporting Information data, code, or model files are submitted outside the Figshare repository, the Supporting Information item added for the manuscript is a figure-legend file. The revised manuscript now includes an in-text citation to S1 File after the final figure discussion, and a Supporting information section has been added at the end of the manuscript with the caption: "S1 File. Figure legends for separately uploaded manuscript figures." The corresponding S1_File_Figure_Legends.docx file lists the captions for Fig1.tif through Fig20.tif, which are uploaded separately as manuscript figure files. The reproducibility data and code remain available through Figshare with DOI: https://doi.org/10.6084/m9.figshare.32901059.

### Journal Requirement 11

**Comment:** Please evaluate reviewer-recommended references.

Response: We reviewed the recommended references and incorporated those that help position the study more clearly. The added literature now distinguishes three roles: direct spatiotemporal forecasting baselines, methodological inspiration from multi-task and graph-based learning, and broader intelligent-transportation perception work that motivates congestion warning as a traffic-perception problem. We did not present all cross-domain papers as direct ATM baselines, because doing so would overstate their comparability. Instead, the revised related-work section uses them where they clarify the methodological or conceptual background of this study.

# Response to Reviewer #1

We thank Reviewer #1 for the positive assessment of the manuscript structure and application value, and for the constructive suggestions on model positioning, labeling reliability, baseline comparison, graph sparsity, and related work.

### Reviewer #1, Comment 1

**Comment:** The stacked GNN-LSTM-Transformer architecture is common in spatiotemporal prediction. Please explain why this specific stacking is suitable for ATM and compare it with end-to-end models such as ST-GCN, ASTGCN, and Informer.

Response: We agree that the original manuscript did not sufficiently explain why this architecture is appropriate for en-route ATM or how it differs from existing end-to-end spatiotemporal models. We revised the Introduction, Section 2.4, and Section 4 to make the motivation explicit. In ATM sector congestion, a warning is not generated only by local traffic volume: congestion can build over several 5-minute windows, spread along route-contiguous and neighboring grids, and appear differently depending on upstream/downstream operating states. For this reason, the GNN component is used to encode grid adjacency, route continuity, and congestion propagation; the BiLSTM component captures the buildup, persistence, and dissipation of operating states over historical windows; and cross-attention fuses spatial propagation representations with temporal operating representations before the 15-minute-ahead prediction. We also added a comparison with ST-GCN, ASTGCN, Informer-type models, DCRNN, and Graph WaveNet. ST-GCN and ASTGCN provide useful references for structured spatiotemporal graphs, but their common settings are usually designed for denser and more regular road-sensor networks, while ATM grids may be sparse, temporarily disconnected, and affected by route-contiguity constraints. Informer is effective for long-sequence temporal modeling, but it does not by itself represent sector-grid topology or upstream/downstream propagation. DCRNN and Graph WaveNet are therefore included as graph-temporal baselines in Table 3, while the proposed architecture is presented as an ATM-adapted design that combines observable grid operation, spatial propagation, and short-term temporal evolution under the raw15 feature setting.

### Reviewer #1, Comment 2

**Comment:** K-means++ labels may cause the supervised model to learn the clustering logic rather than true operational boundaries. Please introduce controller validation or discuss the limitation.

Response: This concern is well taken. We revised the manuscript to make clear that the K-means++ labels are operationally interpreted pseudo-labels rather than manually observed ground truth. To make the labeling process more transparent, the revised version reports the observable feature set used for clustering, the candidate-K validation logic, internal validity indicators, cluster-center interpretation, and the final selection of three operating states. We also added a small expert plausibility check: representative conflict/congestion candidate windows and crowded transition windows were reviewed with personnel familiar with Guangdong and Chongqing regional control contexts. The experts did not treat the labels as absolute truth, but they considered the selected congestion and crowded cases operationally plausible when combined with traffic load, dwell time, maneuvering frequency, and delay evidence. This revision reduces the risk that readers interpret the pseudo-labels as direct manual annotations. These clarifications were added mainly in Section 3.4 and Section 5.3, with the remaining uncertainty discussed in Section 6.

### Reviewer #1, Comment 3

**Comment:** The comparison only includes Linear Regression and LSTM. Please compare with mainstream spatiotemporal graph neural network baselines such as DCRNN and Graph WaveNet.

Response: We agree that the earlier comparison was too narrow. The revised manuscript adds representative temporal and graph-temporal baselines under the same raw15 visible-feature setting and the same 15-minute prediction horizon. Specifically, Table 3 now includes LSTM, DCRNN, Graph WaveNet, and the proposed GNN-BiLSTM-Cross-Attention model. The comparison therefore tests whether the proposed combination of graph propagation, bidirectional temporal encoding, and cross-attention improves rare T2 hotspot recognition beyond sequence-only and graph-temporal alternatives. The new table also reports L2-focused metrics rather than relying only on overall performance, which is important because severe congestion is the rare and operationally critical class.

### Reviewer #1, Comment 4

**Comment:** The flight-flow graph may be extremely sparse at a 5-minute resolution, producing isolated nodes. Please explain how isolated nodes are handled and discuss the impact of graph sparsity.

Response: We agree that 5-minute flight-flow graphs may contain weakly connected or temporarily isolated grids. The revised method description now explains how these cases are handled. Self-loops preserve each grid's own state, static neighborhood and route-contiguity edges retain physically meaningful context, and dynamic flow edges add time-varying transfer information when flights move between grids. When a grid has few or no traversing flights in a short window, its node-level operating features and static context are still available to the model. This makes the graph formulation less dependent on a dense flow edge set at every time step. This explanation was added to the graph construction description in Section 4.2.

### Reviewer #1, Comment 5

**Comment:** Several recent spatiotemporal traffic congestion prediction references are missing.

Response: We updated the literature discussion to include the recent spatiotemporal traffic-event and graph-prediction studies suggested by the reviewer. These references are used to clarify how related fields model rare traffic events, tipping points, and graph-based temporal propagation, while the revised text also explains why ATM sector congestion differs from road-traffic event prediction. This helps position the proposed framework as an ATM-specific adaptation rather than a direct transfer of a road-network method. The related discussion and references were added in Section 2.3 and the reference list.

# Response to Reviewer #2

We thank Reviewer #2 for the constructive comments regarding technical contribution, clustering transparency, related work, supervised cause-weight ground truth, reproducibility, and deployment conditions.

### Reviewer #2, Q1

**Comment:** The manuscript combines existing techniques. Please better distinguish the technical contribution from existing spatiotemporal prediction models.

Response: We agree that the contribution needed to be stated as more than a combination of known components. The revision now frames the contribution around mechanism alignment and reproducibility. K-means++ converts observable operational indicators into three interpretable pseudo-label states; the GNN represents spatial and route-related propagation; the BiLSTM captures temporal evolution over historical 5-minute windows; cross-attention fuses spatial and temporal representations; and SHAP/SHEP attribution explains model warnings after inference rather than adding an unverifiable supervised cause-regression target. This reframing makes the contribution clearer: the paper is not claiming novelty from each module in isolation, but from an ATM-oriented pipeline that connects label construction, spatiotemporal prediction, and post-hoc operational explanation. This reframing is reflected in the contribution statements in the Introduction and in the method description in Sections 3.4 and 4.

### Reviewer #2, Q2

**Comment:** Please provide more details on K-means++ cluster number selection, cluster center interpretation, silhouette coefficient, elbow analysis, and expert validation.

Response: We agree that the clustering procedure needed to be more transparent. The revised manuscript now reports the observable clustering variables, normalization, candidate-K comparison, internal validity metrics, cluster-center interpretation, and the expert plausibility check. We also clarified that K=3 is supported by the data-driven validation and the operational interpretation of free-flow, crowded transition, and congested states, rather than being imposed after the fact to match a desired label count. This is important because the downstream model is trained on these pseudo-labels, so readers need to understand both the statistical and operational basis of the labeling process. These details are now reported in Section 3.4, Section 5.3, Figure 13, Figure 14, and Table 2.

### Reviewer #2, Q3

**Comment:** Please expand the related work with additional intelligent transportation, visual perception, and multi-task learning studies.

Response: We considered the recommended cross-domain works and incorporated them where they help the paper's logic. The dual-pathway driver-emotion study is used as a methodological reference for combining shared and task-specific representations; the M-GBT short-term load-forecasting work helps motivate regional graph coupling and Transformer-style temporal modeling; and visual perception studies are discussed as conceptual support for treating congestion prediction as an intelligent transportation perception problem. At the same time, the revision avoids presenting these studies as direct ATM baselines, because their data, operational constraints, and evaluation targets differ from sector congestion warning. These references and distinctions were added to Sections 2.3 and 2.4.

### Reviewer #2, Q4

**Comment:** The ground truth for the cause factor weight regression task is unclear.

Response: We agree that the original cause-weight regression description could be misunderstood as relying on unavailable ground-truth cause weights. We therefore made a substantive methodological revision. The supervised training objective now focuses on future congestion-level prediction and T2 hotspot warning, both using information available before the prediction time. The previous supervised cause-regression head and its multi-task loss were removed. Causal explanation is now handled after inference through SHAP/SHEP-style grouped attribution: the first-level chart separates external triggering risk from congestion-propagation risk, and the second-level chart decomposes the external component into weather, restricted-area, controller-workload, and wake-intensity contributions. This change better matches the available data, avoids unverifiable cause labels, and keeps explanation separate from the predictive training target. The corresponding method revision is described in Section 4.4, and the revised attribution presentation is used in Figure 5, Figure 19, and Figure 20.

### Reviewer #2, Q5

**Comment:** The data availability statement indicates that some processed data and scripts are available only upon reasonable request. Please provide reproducibility materials such as anonymized data, feature matrices, adjacency matrices, code, parameter settings, and preprocessing scripts.

Response: We agree that the data and code availability statement needed to be stronger and more specific. The corresponding de-identified grid-level dataset and all author-generated code needed to reproduce the study have been deposited on Figshare with DOI: https://doi.org/10.6084/m9.figshare.32901059. The package includes feature matrices, K-means++ validation data and model files, graph construction inputs, training/validation/test splits, model training and evaluation scripts, baseline scripts, robustness-test scripts, figure source data, and the interface replay input. These released materials are designed to let readers reproduce the reported labels, model results, and visual case study using the same de-identified data basis as the revised manuscript. The availability information will be provided through the online submission system and the Figshare repository record rather than as a separate funding or data statement in the manuscript text.

### Reviewer #2, Q6

**Comment:** The manuscript claims real-time application and decision-support value but does not sufficiently analyze practical deployment conditions.

Response: We agree that a real-time or operational-decision claim should be supported by deployment information, not only accuracy metrics. The revised manuscript now reports the dominant complexity terms for graph propagation, recurrent temporal encoding, and attention fusion, along with parameter count and empirical inference-time estimates. We also clarify that the system is intended for minute-level tactical warning within a 5-minute data update cycle, not for millisecond-level aircraft control. This makes the operational scope more precise and prevents the accuracy results from being interpreted as a complete deployment evaluation. The complexity and deployment discussion was added in Section 5.4.

# Response to Reviewer #3

We thank Reviewer #3 for the detailed and helpful comments on generalization, pseudo-labeling, threshold interpretation, computational cost, vertical dimensions, adjacency construction, robustness, deployment, and sustainability claims.

### Reviewer #3, Comment 1

**Comment:** The validation is limited to a single sector and one year of data. Please discuss generalization to other airspace structures, traffic densities, and control procedures.

Response: We agree that the empirical scope should be described carefully. In the revision, we distinguish between the generality of the method and the sector-specific nature of the trained model. The proposed framework is based on general data-driven and machine-learning procedures, including grid-level feature construction, K-means++ operating-state labeling, graph-temporal prediction, and post-hoc SHAP/SHEP attribution; these procedures are not limited to ZGGGAR15 in principle. However, the trained parameters, graph structure, normalization range, clustering centers, and warning threshold are learned from the Chinese ZGGGAR15 sector data used in this study. Therefore, when the framework is applied to another sector with different airspace geometry, traffic density, route layout, weather exposure, or control procedures, the local grid definition and graph should be rebuilt and the model should be recalibrated or retrained with the corresponding sector data. Section 6 now states this distinction more explicitly: the method is transferable, whereas the current trained model and numerical settings are sector-specific.

### Reviewer #3, Comment 2

**Comment:** K-means++ labels depend on feature selection and normalization. Please discuss whether the labels were reviewed by experienced controllers.

Response: We agree that K-means++ labels are sensitive to feature choice and normalization. The revised manuscript now states the clustering feature set explicitly and explains that external factors such as weather, restricted areas, controller workload, and wake intensity are not used to define the clusters. This separation is important because the clusters are intended to reflect internal operating-state congestion, while external variables are used later as predictors and attribution factors. We also added the expert plausibility check and a limitation noting that larger controller-labeled datasets would further strengthen future validation. These clarifications appear in Section 3.4, Section 5.3, and Section 6.

### Reviewer #3, Comment 3

**Comment:** The operational explanation of the binary hotspot threshold theta is weak.

Response: We agree that the operational meaning of the binary hotspot threshold theta needed to be stated more plainly. The model first outputs a probability that a grid-time unit will become a T2 hotspot within the 15-minute prediction horizon. The threshold theta is the alarm line used to convert this probability into an operational warning: when the predicted T2 probability is greater than or equal to theta, the grid is marked as a hotspot; otherwise it is not. In the revised manuscript, theta is selected on the validation segment to balance missed severe-congestion warnings and false alarms, and then it is fixed before testing. Thus, theta should be read as a warning-sensitivity setting rather than a new physical congestion index: a lower theta would issue more alerts earlier, while a higher theta would be more conservative. The manuscript now explains this rule in Section 3.4 and Section 5.4, with the fixed operating point illustrated in Figure 18.

### Reviewer #3, Comment 4

**Comment:** The hybrid architecture is complex, but computational complexity is insufficiently discussed.

Response: We agree that architecture complexity must be discussed together with computational feasibility. The revised manuscript reports the main costs of graph propagation, recurrent temporal encoding, and attention fusion, and relates them to the 5-minute update cycle used in the study. The model has approximately 0.18 million trainable parameters, with forward inference around 0.09 seconds on GPU and 0.68 seconds on CPU in the reported setting; the rolling update workflow is estimated at 20-45 seconds. These values do not replace a field deployment test, but they show that the method is compatible with the tactical warning interval considered in the paper. The computational-cost discussion is included in Section 5.4.

### Reviewer #3, Comment 5

**Comment:** The missing vertical dimension requires more critical discussion.

Response: We agree that the two-dimensional grid abstraction requires a more careful discussion. The revised manuscript explains that the model primarily represents horizontal grid interactions, while vertical operational complexity is partly reflected through altitude-change frequency and related maneuver indicators. We also clarified that this is a simplification: a full three-dimensional airspace graph could better represent altitude-layer interactions, vertical separation constraints, and climbing/descending conflicts. This limitation is now stated explicitly as an important direction for future work. This clarification appears in Section 3.2, Section 5.1, and Section 6.

### Reviewer #3, Comment 6

**Comment:** It is unclear whether the performance gain is worth the additional architecture complexity.

Response: We agree that performance improvement alone is not enough to justify a more complex model. The revised manuscript now connects complexity to operational value by comparing the proposed framework with LSTM, DCRNN, and Graph WaveNet under the same raw15 input setting. The comparison focuses on rare T2 hotspot precision, recall, F1-score, and average precision, because those metrics reflect the warning task more directly than overall accuracy. The accompanying computational-cost discussion then shows that the additional graph and attention components remain feasible within the intended 5-minute update cycle. The model comparison is reported in Section 5.5 and Table 3, with computational feasibility discussed in Section 5.4.

### Reviewer #3, Comment 7

**Comment:** The adjacency matrix based only on flight-flow transfer may oversimplify operational relationships such as workload balancing or rerouting constraints.

Response: We agree that direct flight-flow transfer alone cannot represent all operational relationships. The revised graph description therefore includes static neighboring-grid context and route-contiguity information in addition to dynamic flow edges. The limitations section also acknowledges that some operational relationships, such as workload balancing, rerouting constraints, common weather exposure, and restricted-area effects, may be better represented by a future dynamic multi-relation graph. This clarifies what the current graph captures and what remains outside its scope. The graph construction was revised in Section 4.2, and the remaining limitation is discussed in Section 6.

### Reviewer #3, Comment 8

**Comment:** Please cite Bakirci (10.1016/j.engappai.2025.111775) to strengthen the deployment discussion.

Response: We added the suggested deployment-related reference and used it to strengthen the discussion of accuracy-latency tradeoffs and resource-aware AI deployment. We cite it as a deployment and resource-efficiency reference rather than as a direct ATM congestion baseline. This keeps the related-work comparison accurate while still using the reference to support the practical deployment discussion. The added reference is discussed in Sections 2.3-2.4 and linked to deployment considerations in Section 5.4.

### Reviewer #3, Comment 9

**Comment:** Please discuss data imbalance, because severe congestion events are much rarer than normal operation.

Response: We agree that class imbalance is central to the problem, because severe congestion is much rarer than normal operation. The revised manuscript therefore emphasizes L2 precision, recall, F1-score, and average precision rather than relying on overall accuracy. We also describe the use of balanced temporal sampling and hotspot-oriented loss weighting so that training does not become dominated by free-flow windows. This makes the evaluation more consistent with the operational objective: detecting rare but consequential congestion warnings. The imbalance handling and L2-focused metrics are described in Section 5.4 and reported in Figure 18 and Section 5.5.

### Reviewer #3, Comment 10

**Comment:** There are no confidence intervals, repeated-run statistics, or robustness evaluation under noisy conditions.

Response: We agree that a single run is insufficient evidence for robustness. The revised manuscript adds five-seed repeated training and controlled perturbation tests, including Gaussian feature noise, random feature masking, and time-window dropout. The results show that the proposed model remains stable under moderate input disturbance, while also revealing that temporal-window dropout has the largest effect, which is consistent with the role of short-term congestion evolution in the model. This gives readers a clearer view of both stability and sensitivity. These robustness results are reported in Section 5.5 and Table 4.

### Reviewer #3, Comment 11

**Comment:** Real-time prediction is mentioned, but actual inference latency and deployment feasibility are not analyzed.

Response: We agree that the meaning of real-time prediction should be made concrete. The revised manuscript now reports model size, GPU/CPU inference time, rolling update time, and the intended 5-minute data update cycle. It also clarifies that the May 13, 2023 09:15 replay case uses the already trained model for 15-minute-ahead prediction and visualization, without retraining or parameter adjustment for that case. This helps distinguish model evaluation, operational replay, and visualization functions. The real-time interpretation is clarified in Section 5.4, while the replay case is described with Figure 19 and Figure 20 in Section 5.5.

### Reviewer #3, Comment 12

**Comment:** The sustainability and carbon-reduction claims appear speculative because no direct emissions or fuel-saving analysis is included.

Response: We agree that the sustainability discussion should not imply a direct emissions result. We revised the manuscript to state that the present work addresses congestion prediction under the FF-ICE operational concept and can provide indirect support for future fuel-use and emissions analysis. Direct carbon-emission quantification is now treated as outside the scope of this study and as future work requiring dedicated fuel-burn and emissions models. This revision avoids overstating the environmental conclusion while still acknowledging the operational relevance of earlier congestion warning. The wording was adjusted in the Abstract, Conclusion, and Section 6.

# Closing Statement

We again thank the Academic Editor and reviewers for their constructive feedback. We believe that the revised manuscript has been substantially improved in methodological explanation, experimental comparison, reproducibility, deployment analysis, and discussion of limitations. We hope that the revised version is now suitable for further consideration by PLOS ONE.

Sincerely,

Xiaoyu Yan, on behalf of all authors
