# Supplementary material for: An operational spatiotemporal prediction framework for sector congestion in air traffic management
Source: PLoS One. 2026 Oct 27;21(10):e0355927. doi: 10.1371/journal.pone.0355927 (PMC13712534; doi:10.1371/journal.pone.0355927)
Supplement: Supplementary file 3 [file pone.0355927.s003.docx]

Dear Dr. Weaver and Reviewers,

We sincerely thank the editor and the reviewers for their careful evaluation of our manuscript and for the constructive comments, which have helped us improve the clarity, rigor, and presentation of this work. Below we respond to the comments from the current review round and describe the corresponding changes in the manuscript, figures, and evaluation materials.

# Response to Reviewer #4

We thank Reviewer #4 for the detailed comments on data provenance, graph notation, training objectives, operational interpretation, figure readability, and terminology. These comments led us to make the method and experimental presentation more explicit while preserving the implemented prediction framework.

### Reviewer #4, Comment 1

**Comment:** The sources of the variables in Table 1 and the main processing steps from the source data to the model inputs are not sufficiently clear.

**Response:** Thank you for pointing out that the data lineage was not sufficiently transparent. This comment prompted us to distinguish the source observations, the intermediate five-minute grid records, and the variables actually exposed to the model. We expanded Section 3 to describe the processing chain from ADS-B trajectories, meteorological radar products, restricted-area notices, controller-operation records, and wake-related information through cleaning, temporal alignment, spatial mapping, five-minute aggregation, and feature generation. We also revised Table 1 to identify the source and construction of each variable. In particular, weather intensity is derived from matched weather observations, restricted-area impact and controller workload are constructed indicators rather than direct raw records, wake intensity is derived from ADS-B aircraft and traffic-state information, and congestion level and label confidence are generated through K-means++ clustering and label diagnostics. The revised description makes the boundary between source data, derived indicators, and model inputs explicit, thereby improving methodological transparency and reproducibility.

### Reviewer #4, Comment 2

**Comment:**Section 4.2 should clarify the aggregation interval and whether the adjacency coefficienta_ij_has a time dimension.

**Response:**We appreciate this clarification request. We revised Section 4.2 to state that each graph snapshot represents one five-minute aggregation window and that the 15-minute prediction horizon corresponds to three subsequent time steps. The adjacency matrix is written as the static matrix A=[a_ij_], defined from spatial neighborhood and route-contiguity relations; it does not carry a time index. Temporal variation is represented by the node-feature sequenceX^(t)^and the historical graph sequence, rather than by changing the notation of A. The same convention is now used in the related equations and Figure 6A. This removes ambiguity about the graph structure and makes the temporal modeling procedure easier to reproduce.

### Reviewer #4, Comment 3

**Comment:**In Eq. (10), the meaning of the superscript t inH^(t,l)^should be defined.

**Response:**Thank you for identifying this notation gap. We added a definition immediately after Eq. (10): t denotes the time step in the historical five-minute sequence, l denotes the graph layer, andH^(t,l)^denotes the node representation at time t after graph layer l. We checked Eqs. (10)-(15) and the accompanying text so that t consistently denotes time and l consistently denotes the graph layer. This local clarification prevents readers from confusing a time index with a layer index and improves the readability of the model derivation.

### Reviewer #4, Comment 4

**Comment:**The notation in Figure 6A, includingA_(n-1)_^(t,l-1)^, appears inconsistent with the assumption of a static adjacency matrix.

**Response:**We agree that the original annotation could be read as implying a time-varying adjacency matrix. Figure 6A has been revised so that the graph structure is labeled consistently as the static adjacency matrix A, and the notation in the surrounding explanation has been aligned with this convention. The revised text explicitly states that route and neighborhood relationships remain fixed across graph snapshots, while time-varying operating conditions are carried byX^(t)^and the historical sequence. This correction makes the figure consistent with the mathematical assumption used by the model.

**Revised Figure 6A. Static adjacency matrix and graph update representation.**

### Reviewer #4, Comment 5

**Comment:**The loss function in Eq. (39) does not provide enough detail aboutL_cls_andL_hotspot_.

**Response:**Thank you for requesting the missing training details. We expanded the explanation after Eq. (39) by definingL_cls_as the class-weighted focal loss for the three congestion states T0, T1, and T2, andL_hotspot_as the binary focal loss for T2 hotspot identification. The revised text reports the implemented settingsγ_cls_=1.5,α_hotspot_=0.80,γ_hotspot_=2.0, and the combined objective L=L_cls_+0.50L_hotspot_. It also clarifies that the attribution output is computed after inference and is not an additional supervised loss term. These additions make the optimization target and the role of the hotspot head directly auditable without changing the model architecture or reported experiments.

### Reviewer #4, Comment 6

**Comment:** Figure 16 is difficult to interpret, and the sources and meanings of controller load and traffic-flow variables should be clarified.

**Response:** We appreciate this comment because the original wording overstated what the figure represented. Figure 16 is a violin plot with sampled observations and summary markers; it compares controller-workload groups with speed-adjustment frequency, rather than directly comparing controller load with traffic flow. We revised the figure caption and the corresponding paragraph in Section 5.3 to define controller workload as a derived operational-pressure indicator and speed-adjustment frequency as the number of speed-adjustment events per observed aircraft in a five-minute grid window. We also clarified that the figure is descriptive and is not used to claim causality. The correction improves the alignment among the plotted variables, their data sources, and the interpretation of the result.

**Revised Figure 16. Speed-adjustment frequency across controller-load groups.**

### Reviewer #4, Comment 7

**Comment:** Please explain why a 15-minute prediction horizon was selected and how it relates to air traffic flow management actions.

**Response:** Thank you for encouraging us to make the operational meaning of the prediction horizon explicit. We added an explanation in the model-training and prediction description: with five-minute aggregation, a 15-minute horizon corresponds to three future time steps and is long enough to identify the buildup and spatial spread of congestion while remaining relevant to short-term flow-management coordination. It can provide time for tactical measures such as speed control, metering, rerouting coordination, or holding preparation, without presenting the result as a replacement for longer-horizon strategic planning. We also clarify that 30-minute and 60-minute horizons would require separately trained and validated models. This establishes the practical rationale and scope of the selected horizon.

### Reviewer #4, Comment 8

**Comment:** Please discuss how FF-ICE/R2 shared trajectory information could contribute to future prediction.

**Response:** We appreciate this forward-looking suggestion. We added a discussion in the Introduction and Discussion explaining that FF-ICE/R2 planning and trajectory information could provide earlier indications of demand, route pressure, and potential downstream congestion. Such information could be fused with observed operational states to improve future-horizon prediction and support coordinated flow-management planning. We present FF-ICE/R2 as a possible complementary information source for future development, while keeping the current prediction method based on the operational data and feature definitions evaluated in this study. This distinction makes the future extension clear without changing the evidence base of the present work.

### Reviewer #4, Comment 9

**Comment:** The literature review is concentrated on Chinese studies; representative ATM research from Europe and the United States should also be included.

**Response:** Thank you for this important positioning suggestion. We expanded and checked the Introduction and Related Work sections to include representative international ATM and spatiotemporal forecasting research relevant to capacity management, trajectory information, and operational prediction. We also clarified the different roles of these studies: they provide international operational and methodological context, whereas the present work focuses on short-term en-route sector congestion-state prediction and hotspot localization. This broader comparison places the contribution in a more balanced international research context without treating studies with different data and targets as direct one-to-one baselines.

### Reviewer #4, Comment 10

**Comment:** The notation and the meanings of K1 and K2 in Sections 3.1-3.2 and Figure 2 should be made consistent.

**Response:** We appreciate this notation check. We reviewed Sections 3.1 and 3.2 and Figure 2 together, defined the coordinate and operational variables at their first occurrence, and corrected the correspondence of K1 and K2 in the figure and caption. Figure 2 now uses the intended source-information grouping, while the text consistently distinguishes spatial or operational structure from external and management-related information. The definitions of P_i(X_i,Y_i), H_max and H_1max, (lambda, phi, h), (v, alpha), and Type(P) were also clarified where needed. These local corrections reduce symbol ambiguity and ensure that the figure can be read consistently with the equations and narrative.

**Revised Figure 2. Corrected K1 and K2 source-information labels.**

### Reviewer #4, Comment 11

**Comment:** The height labels in Figure 9 should use ft or FL rather than m, or otherwise be clearly defined.

**Response:** Thank you for identifying the unit inconsistency. We revised Figure 9 and its caption so that altitude values are displayed in ft where numerical altitude is shown, and flight-level labels use FL where standardized flight levels are shown. The text explains that metric units may be used internally for geometric calculations, but the figure presentation follows the operational altitude convention. This separates computational units from operational display units and makes the vertical information easier to interpret.

**Revised Figure 9. Traffic distribution with operational altitude notation.**

### Reviewer #4, Comment 12

**Comment:** The horizontal and vertical axes in Figure 10 are not sufficiently clear.

**Response:** We agree that the original axis descriptions were too abbreviated. Figure 10, its caption, and the related paragraph were revised to identify the horizontal variable, the vertical variable, the observation period, and the statistical meaning of the plotted values. The wording now distinguishes the number of flights from broader traffic-volume indicators and states how the values were aggregated for the ZGGGAR15 sector. These changes make the plotted relationship and its time scale unambiguous without changing the underlying data.

**Revised Figure 10. Monthly traffic volume and flight-delay time in ZGGGAR15.**

### Reviewer #4, Comment 13

**Comment:** The correspondence between routes and flight levels in Figure 12 is unclear.

**Response:** Thank you for pointing out this important operational detail. We revised Figure 12 to preserve the original route structure while adding the corresponding flight-level information and key waypoints. The caption and text now explain that the route-level display follows the applicable Chinese civil-aviation flight-level convention, including the direction-dependent odd/even flight-level assignment, and that FL is used for the displayed flight levels. Panel A presents the horizontal route and sector geometry, while Panel B gives the representative vertical route-level correspondence. This makes the relationship between route direction and altitude layer clear without implying that the figure uses arbitrary constructed levels.

**Revised Figure 12. Horizontal route geometry and vertical flight-level correspondence.**

### Reviewer #4, Comment 14

**Comment:** The Label Code column in Table 2 lacks units and the meanings of the reported variables are not sufficiently specified.

**Response:** We appreciate this request for a more precise table definition. We revised the note below Table 3 to distinguish dimensionless labels from measured or aggregated variables. The note now explains that time_step represents a five-minute aggregation window, traffic_load is the number of distinct aircraft per grid window, average dwell time is reported in minutes, and speed-adjustment, heading-deviation, altitude-change, and waiting-instruction values are event frequencies per observed aircraft per five-minute grid window. The label code is a dimensionless K-means++ category, while congestion thresholds are expressed on the corresponding feature scales. This makes the table auditable and prevents label codes from being mistaken for physical measurements.

### Reviewer #4, Comment 15

**Comment:** Section 5.4 repeats material already presented in Section 4 and should be made more concise.

**Response:** Thank you for noting the repetition. We revised Section 5.4 so that Section 4 remains the location for the general model formulation, equations, and mechanism description, whereas Section 5.4 focuses on the implemented training and validation configuration. Repeated derivations were removed or shortened, and the section now reports the model settings used in the experiment, including the graph encoder, BiLSTM, cross-attention module, sequence length, prediction horizon, and computational-cost measurements. This separation improves the manuscript flow and allows readers to distinguish the general method from its experimental instantiation.

### Reviewer #4, Comment 16

**Comment:** Abbreviations such as FF-ICE, T0/T1/T2 hotspot, SHAP/SHEP, and GAT should be expanded at first use and used consistently thereafter.

**Response:** We appreciate this careful presentation check. We reviewed the manuscript, equations, figure captions, tables, and response text to expand abbreviations at first use and standardize their later forms. Graph attention network (GAT), FF-ICE, the T0/T1/T2 congestion-state notation, and the T2 hotspot definition are now introduced with their operational meanings. The post-hoc grouped attribution terminology is also kept consistent with the implementation and the figure captions. This editorial revision reduces the cognitive load for readers and prevents the same symbol from being interpreted differently in different sections.

# Response to Reviewer #5

We thank Reviewer #5 for the comments on chronological evaluation, state-transition behavior, application scope, notation, and parameter definitions. These comments helped us make the evaluation protocol and the operational meaning of the reported results easier to verify.

### Reviewer #5, Comment 1

**Comment:** The chronological data split and the controls against information leakage are not described transparently enough. Please provide the exact training, validation, and test periods and explain how preprocessing and model selection use only permissible information.

**Response:** Thank you for raising this central reproducibility issue. We added the exact chronological split to the experimental description: training data cover 2023-01-01 to 2023-10-19, validation data cover 2023-10-20 to 2023-11-24, and test data cover 2023-11-25 to 2023-12-31. We also clarified that the historical input sequence and future target windows are formed within the chronological workflow, that scaling and model-selection decisions are determined through the training/validation procedure, and that the test segment is reserved for final evaluation. The released audit materials record the split and the associated preprocessing and evaluation files. These additions make the evaluation protocol and its temporal ordering explicit.

### Reviewer #5, Comment 2

**Comment:** Please include a persistence or last-state baseline, or provide a state-transition analysis, to show whether the model learns changes in congestion rather than simply copying the current state.

Response: We appreciate this suggestion because both representative prediction baselines and a current-state continuation reference are necessary for short-horizon congestion prediction. We added reproducible LSTM, DCRNN, and Graph WaveNet baselines in Section 5.5 and Table 4. They use the same processed grid-level table, chronological split, 24-step history, and 15-minute prediction horizon as the proposed framework; training-period normalization, positive-class weighting, and validation-set threshold selection are applied under the common evaluation protocol. The proposed framework achieves an L2 F1-score of 0.9602 and AP of 0.9767, compared with L2 F1-scores of 0.6172, 0.6935, and 0.3256 for LSTM, DCRNN, and Graph WaveNet, respectively; its AP also exceeds the strongest baseline AP of 0.7450. We also added a 15-minute state-transition analysis in Section 5.5 and Table 5. The transition-specific results shown below cover escalation, recovery, and persistent T2 cases, demonstrating performance beyond simply copying the current state. In Table 5, Transition identifies the current-to-future congestion-state change; Events is the number of observed cases; Precision and Recall measure transition-specific detection; Model F1 is the proposed model's F1-score; and Persistence F1 is the last-state reference.

**Table 5. Test-set state-transition evaluation and persistence reference for the 15-minute horizon.**

| **Transition** | **Events** | **Precision** | **Recall** | **Model F1** | **Persistence F1** |
| --- | --- | --- | --- | --- | --- |
| T0 to T1 | 104 | 0.7027 | 1.0000 | 0.8254 | 0.0000 |
| T1 to T2 | 40 | 0.8696 | 1.0000 | 0.9302 | 0.0000 |
| T2 to T2 | 445 | 0.9650 | 0.9910 | 0.9778 | 0.9448 |
| T2 to T1 | 52 | 0.9000 | 0.6923 | 0.7826 | 0.0000 |
| T1 to T0 | 166 | 1.0000 | 0.8855 | 0.9393 | 0.0000 |

### Reviewer #5, Comment 3

**Comment:** The application claims should be moderated because the validation is retrospective and based on one sector and one year of data.

**Response:** Thank you for encouraging a more precise statement of the application scope. We revised the Abstract, Discussion, and Conclusion so that the system is presented as a data-driven decision-support framework demonstrated on the ZGGGAR15 sector and 2023 retrospective data, rather than as a universally validated operational system. The revised wording retains the practical value of real-time data integration, short-term warning, spatial hotspot localization, and post-hoc explanation, while avoiding claims that the reported results automatically generalize to all sectors or operating environments. This makes the practical contribution clearer and keeps the strength of the claims proportional to the evidence presented.

### Reviewer #5, Comment 4

**Comment:** The manuscript should use one spelling consistently for spatiotemporal and spatio-temporal.

**Response:** Thank you for identifying this consistency issue. We conducted a terminology pass across the title, abstract, keywords, main text, equations, figure captions, table notes, and response materials, and standardized the manuscript to the form spatiotemporal. This small revision removes avoidable variation and makes the terminology consistent throughout the paper.

### Reviewer #5, Comment 5

**Comment:**The notationd_out_in Eq. (16) is inconsistent withF_out_used later for the output dimension.

**Response:** We appreciate this notation check. We reviewed Eq. (16), the surrounding derivation, and the later model-description paragraphs and aligned the output-dimension notation with the implemented model. The first occurrence now defines the output dimension explicitly, and subsequent equations and captions use the same symbol consistently. This local notation correction removes a possible ambiguity without changing the model computation, output structure, or reported results.

### Reviewer #5, Comment 6

**Comment:** The parameters in Table 2 need clearer units, aggregation periods, and threshold definitions.

**Response:** Thank you for requesting this additional precision. We revised the Table 3 note to specify the five-minute aggregation period, the unit of average dwell time, and the event-frequency interpretation of the maneuver-related variables. We also clarified that the congestion label is a dimensionless K-means++ category, while thresholds and cluster centers remain on the relevant feature scales. These definitions are now aligned with the feature-generation description and the equations, so readers can interpret the reported parameters without inferring units or time windows from the column names alone.

We sincerely thank the reviewers again for their time and constructive suggestions. The revisions have improved the transparency of the data-processing chain, the consistency of the mathematical and visual descriptions, and the interpretability of the evaluation results.
