# Supplementary material for: An operational spatiotemporal prediction framework for sector congestion in air traffic management
Source: PLoS One. 2026 Oct 27;21(10):e0355927. doi: 10.1371/journal.pone.0355927 (PMC13712534; doi:10.1371/journal.pone.0355927)
Supplement: Supplementary file 4 [file pone.0355927.s004.docx]

Dear Dr. Liu and Reviewer

We sincerely thank the Academic Editor for the opportunity to revise our manuscript and Reviewer for the careful follow-up assessment. We sincerely apologize for our oversight: in the previous round, we inadvertently did not upload the correct version of the manuscript, which resulted in the two points raised by Reviewer not being reflected in the manuscript. We are very grateful to Reviewer for pointing this out and for the careful follow-up. We have now revised the manuscript accordingly.

**Comment 1**

**Comment:** In Eq. (10), the meaning of the superscript *t* in *H^(t,l)^* should be defined.

**Response:** Thank you for identifying this notation that needed clarification. We revised the paragraph immediately following Eq. (10) to define the time-step index *t*, the graph-layer index *l*, and the representation *H^(t,l)^*. In the same paragraph, we clarified the role of the static adjacency matrix so that the time dependence of the model representations is not confused with changes in graph topology. The revised manuscript text is as follows:

*In Eq. (10), ⊙ denotes element-wise multiplication, and* **1** *is an all-ones vector of compatible dimension. The index t denotes the time step in the historical five-minute sequence, l denotes the graph layer, and H^(t,l)^ denotes the node representation at time t after graph layer l. The adjacency matrix A remains static across graph snapshots, while temporal variation is represented by the node-feature sequence and the time-indexed hidden representations.*

**Comment 2**

**Comment:** Please explain why a 15-minute prediction horizon was selected and how it relates to air traffic flow management actions.

**Response:** Thank you for encouraging us to clarify the operational rationale. We revised the prediction-horizon discussion to explain that, with five-minute aggregation, 15 minutes corresponds to three future steps and balances tactical warning lead time against uncertainty in rapidly changing traffic conditions. We also connected the rolling five-minute updates to short-term ATFM coordination actions and clarified that this horizon is intended for tactical pre-warning, not strategic planning. The revised manuscript text is as follows:

*Operationally, the 15-minute horizon is selected as a compromise between warning lead time and forecast reliability. With five-minute aggregation, it corresponds to three future steps, which provides enough lead time to identify the buildup and spatial spread of congestion before the next few tactical decision cycles, while limiting uncertainty growth in a rapidly changing en-route traffic state. It also matches the rolling-update logic of the framework: new observations can be incorporated every five minutes, while a 15-minute warning remains actionable for short-term ATFM coordination, such as speed control, metering, rerouting coordination, or preparation for holding. The horizon is therefore intended for tactical pre-warning rather than long-horizon strategic planning.*

**Additional change in the future-work section:** To avoid suggesting that the current 15-minute model can be extrapolated directly to longer horizons, we added the following clarification:

*Finally, the present study evaluates a 15-minute-ahead warning horizon. Future work will develop and validate models for longer horizons, including 30- and 60-minute forecasts, and will compare their accuracy, calibration, uncertainty growth, and usefulness for strategic and pre-tactical ATFM planning. These extensions will require horizon-specific training and evaluation rather than direct extrapolation of the current 15-minute model.*

We have also reviewed the reference list and confirmed that it is complete and correct. We again thank the Academic Editor and Reviewer for their time and constructive guidance. We hope these clarifications adequately address the remaining comments.

Sincerely,

Xiaoyu Yan

On behalf of all authors
