# Supplementary material for: An operational spatiotemporal prediction framework for sector congestion in air traffic management
Source: PLoS One. 2026 Oct 27;21(10):e0355927. doi: 10.1371/journal.pone.0355927 (PMC13712534; doi:10.1371/journal.pone.0355927)
Supplement: Peer review history

# Peer review: Decision Letter 0

**DOI:** 10.1371/journal.pone.0355927.r001

**Authors:** Guangyin Jin

26 May 2026

Dear Dr. YAN,

Thank you for submitting your manuscript to PLOS ONE. After careful consideration, we feel that it has merit but does not fully meet PLOS ONE’s publication criteria as it currently stands. Therefore, we invite you to submit a revised version of the manuscript that addresses the points raised during the review process.

Please submit your revised manuscript by Jul 10 2026 11:59PM. If you will need more time than this to complete your revisions, please reply to this message or contact the journal office at plosone@plos.org. When you’re ready to submit your revision, log on to https://www.editorialmanager.com/pone/ and select the 'Submissions Needing Revision' folder to locate your manuscript file.

- A letter that responds to each point raised by the academic editor and reviewer(s). You should upload this letter as a separate file labeled 'Response to Reviewers'.
- A marked-up copy of your manuscript that highlights changes made to the original version. You should upload this as a separate file labeled 'Revised Manuscript with Track Changes'.
- An unmarked version of your revised paper without tracked changes. You should upload this as a separate file labeled 'Manuscript'.

If you would like to make changes to your financial disclosure, please include your updated statement in your cover letter. Guidelines for resubmitting your figure files are available below the reviewer comments at the end of this letter.

If applicable, we recommend that you deposit your laboratory protocols in protocols.io to enhance the reproducibility of your results. Protocols.io assigns your protocol its own identifier (DOI) so that it can be cited independently in the future. For instructions see: https://journals.plos.org/plosone/s/submission-guidelines#loc-laboratory-protocols. Additionally, PLOS ONE offers an option for publishing peer-reviewed Lab Protocol articles, which describe protocols hosted on protocols.io. Read more information on sharing protocols at https://plos.org/protocols?utm_medium=editorial-email&utm_source=authorletters&utm_campaign=protocols.

As the corresponding author, your ORCID iD is verified in the submission system and will appear in the published article. PLOS supports the use of ORCID, and we encourage all coauthors to register for an ORCID iD and use it as well. Please encourage your coauthors to verify their ORCID iD within the submission system before final acceptance, as unverified ORCID iDs will not appear in the published article. *Only*  the individual author can complete the verification step; PLOS staff *cannot*  verify ORCID iDs on behalf of authors.

We look forward to receiving your revised manuscript.

Kind regards,

Guangyin Jin

Academic Editor

PLOS One

Journal Requirements:

When submitting your revision, we need you to address these additional requirements.

1.Please ensure that your manuscript meets PLOS ONE's style requirements, including those for file naming. The PLOS ONE style templates can be found at

https://journals.plos.org/plosone/s/file?id=wjVg/PLOSOne_formatting_sample_main_body.pdf and

https://journals.plos.org/plosone/s/file?id=ba62/PLOSOne_formatting_sample_title_authors_affiliations.pdf

2. PLOS requires an ORCID iD for the corresponding author in Editorial Manager on papers submitted after December 6th, 2016. Please ensure that you have an ORCID iD and that it is validated in Editorial Manager. To do this, go to ‘Update my Information’ (in the upper left-hand corner of the main menu), and click on the Fetch/Validate link next to the ORCID field. This will take you to the ORCID site and allow you to create a new iD or authenticate a pre-existing iD in Editorial Manager.

3. Please note that PLOS One has specific guidelines on code sharing for submissions in which author-generated code underpins the findings in the manuscript. In these cases, we expect all author-generated code to be made available without restrictions upon publication of the work. Please review our guidelines at https://journals.plos.org/plosone/s/materials-and-software-sharing#loc-sharing-code and ensure that your code is shared in a way that follows best practice and facilitates reproducibility and reuse.

4. Please note that funding information should not appear in any section or other areas of your manuscript. We will only publish funding information present in the Funding Statement section of the online submission form. Please remove any funding-related text from the manuscript.

5. We note that you have indicated that there are restrictions to data sharing for this study. PLOS only allows data to be available upon request if there are legal or ethical restrictions on sharing data publicly. For more information on unacceptable data access restrictions, please see http://journals.plos.org/plosone/s/data-availability#loc-unacceptable-data-access-restrictions.

Before we proceed with your manuscript, please address the following prompts:

a) If there are ethical or legal restrictions on sharing a de-identified data set, please explain them in detail (e.g., data contain potentially identifying or sensitive patient information, data are owned by a third-party organization, etc.) and who has imposed them (e.g., a Research Ethics Committee or Institutional Review Board, etc.). Please also provide contact information for a data access committee, ethics committee, or other institutional body to which data requests may be sent.

b) If there are no restrictions, please upload the minimal anonymized data set necessary to replicate your study findings to a stable, public repository and provide us with the relevant URLs, DOIs, or accession numbers. For a list of recommended repositories, please see

https://journals.plos.org/plosone/s/recommended-repositories. You also have the option of uploading the data as Supporting Information files, but we would recommend depositing data directly to a data repository if possible

We will update your Data Availability statement on your behalf to reflect the information you provide.

6. In the online submission form you indicate that your data is not available for proprietary reasons and have provided a contact point for accessing this data. Please note that your current contact point is a co-author on this manuscript. According to our Data Policy, the contact point must not be an author on the manuscript and must be an institutional contact, ideally not an individual. Please revise your data statement to a non-author institutional point of contact, such as a data access or ethics committee, and send this to us via return email. Please also include contact information for the third party organization, and please include the full citation of where the data can be found.

7. Thank you for uploading your study's underlying data set. Unfortunately, the repository you have noted in your Data Availability statement does not qualify as an acceptable data repository according to PLOS's standards.

At this time, please upload the minimal data set necessary to replicate your study's findings to a stable, public repository (such as figshare or Dryad) and provide us with the relevant URLs, DOIs, or accession numbers that may be used to access these data. For a list of recommended repositories and additional information on PLOS standards for data deposition, please see https://journals.plos.org/plosone/s/recommended-repositories.

8. Please provide copies of the images for all figures included in the manuscript.

9. We note you have included a table to which you do not refer in the text of your manuscript. Please ensure that you refer to Table 1 and 2 in your text; if accepted, production will need this reference to link the reader to the Table.

10. Please include captions for your Supporting Information files at the end of your manuscript, and update any in-text citations to match accordingly. Please see our Supporting Information guidelines for more information: http://journals.plos.org/plosone/s/supporting-information.

11. If the reviewer comments include a recommendation to cite specific previously published works, please review and evaluate these publications to determine whether they are relevant and should be cited. There is no requirement to cite these works unless the editor has indicated otherwise.

[Note: HTML markup is below. Please do not edit.]

Reviewer's Responses to Questions

**Comments to the Author**

1. Is the manuscript technically sound, and do the data support the conclusions?

Reviewer #1: Yes

Reviewer #2: Partly

Reviewer #3: Yes

**********

2. Has the statistical analysis been performed appropriately and rigorously?

Reviewer #1: Yes

Reviewer #2: Yes

Reviewer #3: No

**********

3. Have the authors made all data underlying the findings in their manuscript fully available?

The PLOS Data policy

Reviewer #1: Yes

Reviewer #2: Yes

Reviewer #3: No

**********

4. Is the manuscript presented in an intelligible fashion and written in standard English?

Reviewer #1: Yes

Reviewer #2: Yes

Reviewer #3: Yes

**********

Reviewer #1: This is a well-structured, application-driven research paper. The authors propose a data-driven framework for en-route sector congestion prediction, integrating objective congestion identification with deep spatiotemporal modeling. The work innovatively translates multi-source operational data into objective congestion labels and employs a hybrid GNN-LSTM-Transformer architecture for the multi-task prediction of congestion levels, hotspots, and causal weights. The paper achieves outstanding predictive metrics on real-world operational data, offering practical guidance for proactive air traffic flow management. However, there are still some problems need to be improved:

1. The stacked GNN-LSTM-Transformer architecture adopted in this paper is already relatively common in the field of spatiotemporal prediction (e.g., traffic and weather forecasting). The authors need to provide a deeper explanation of why this specific stacking is the optimal choice for the specific context of Air Traffic Management (ATM), rather than just a simple concatenation of modules. It is recommended to deeply contrast this framework with state-of-the-art end-to-end spatiotemporal graph models (e.g., ST-GCN, ASTGCN, Informer) in the Introduction or Related Work sections.

2. The paper uses the unsupervised K-means++ clustering algorithm to generate congestion level labels, which are then used to supervise the training of the deep learning model. Using data generated by clustering to train a deep learning model may result in the model simply learning to approximate the clustering algorithm's mathematical logic, masking actual physical and operational boundaries. It is recommended that the authors introduce a small set of ground-truth labels annotated by ATC experts to validate the reliability of the clustering results, or detail the potential limitations of this approach in the discussion section.

3. In the comprehensive comparative experiments in Table 3, the authors only compared the proposed hybrid framework with traditional statistical methods (Linear Regression) and a single LSTM model. To fully demonstrate the cutting-edge nature and effectiveness of the proposed framework in the current AI landscape, the authors must compare its performance with more mainstream and recognized spatiotemporal graph neural network baselines (such as DCRNN, Graph WaveNet, etc.).

4. The paper mentions that the graph model built on flight flows is inherently sparse because only grid pairs connected by actual flight trajectories have non-zero weights. Given the high temporal resolution of 5 minutes used in the experiments, this dynamic graph may exhibit extreme sparsity within such short time windows. This could hinder the Graph Neural Network (GNN) when aggregating neighbor information, producing isolated nodes. The authors should detail how they handle these isolated nodes and discuss the specific impact of extreme graph sparsity on the model's inference performance.

5. Many relevant literatures in recent years about spatio-temporal traffic congestion prediction have been ignored by the authors in related works. For examples:

[1] Spatio-temporal graph neural point process for traffic congestion event prediction[C]//Proceedings of the AAAI conference on artificial intelligence. 2023, 37(12): 14268-14276.

[2] Exploring to predict the tipping points in traffic flow: A lightweight spatio-temporal information-enhanced neural point process approach[J]. Physica A: Statistical Mechanics and its Applications, 2025: 131122.

[3] JSpatio-temporal graph neural networks for predictive learning in urban computing: A survey[J]. IEEE transactions on knowledge and data engineering, 2023, 36(10): 5388-5408.

Reviewer #2: The manuscript proposes a data-driven framework for en-route sector congestion prediction method. The topic is interesting, while some key issues should be further clarified before acceptance.

Q1: The manuscript combines several existing techniques, including K-means++, GNN, LSTM, Transformer-based attention, and multi-task learning. While the integration is reasonable, the paper does not sufficiently distinguish its technical contribution from existing spatiotemporal prediction models in air traffic or transportation systems.

Q2: The manuscript does not provide sufficient evidence that the K-means++ clusters truly correspond to operationally meaningful congestion levels. The authors should provide more details on the selection of the number of clusters, cluster center interpretation, silhouette scores, elbow analysis, and possibly expert validation from air traffic controllers.

Q3: The literature review is not sufficiently comprehensive. The manuscript discusses several studies on traffic congestion prediction and spatiotemporal learning. I suggest that the authors further expand the related work section and discuss the relevant study, e.g., A Dual-Pathway Driver Emotion Classification Network Using Multi-Task Learning Strategy: A Joint Verification; M-GBT: Multi-region Short-term Electricity Load Forecasting Framework based on Graph Bayesian Transformer Network; Vehicle-Mounted Adaptive Traffic Sign Detector for Small-Sized Signs in Multiple Working Conditions; SpikeTOD: A Biologically Interpretable Spike-Driven Object Detection in Challenging Traffic Scenarios; Metaverse Meets Intelligent Transportation System: An Efficient and Instructional Visual Perception Framework.

Q4: The manuscript reports a causal factor weight estimation task and gives MAE, RMSE, and R² values for this regression task. However, it is not clearly explained how the ground-truth causal weights are obtained.

Q5: The current data availability statement indicates that some processed data and scripts are only available upon reasonable request. For a data-driven deep learning study, the authors should provide as much reproducible material as possible, such as anonymized sample data, processed feature matrices, adjacency matrices, code, parameter settings, and preprocessing scripts.

Q6: The manuscript claims real-time applicability and decision-support value, but the operational deployment conditions are not fully analyzed.

Reviewer #3: The paper deals with a highly relevant problem in modern air traffic management, especially considering the increasing complexity of high-density en-route operations and the growing interest in predictive ATFM systems. The proposed GNN–LSTM–Transformer framework is technically ambitious and the integration of spatiotemporal learning with congestion analysis is generally fine. However, the paper still has several shortcomings that need to be addressed. See my comments below:

1) Firstly, you frequently emphasize operational applicability, but the validation is still limited to a single sector and a single year of data. So, it is difficult to know whether the framework would generalize well to different airspace structures, traffic densities, or ATC procedures outside the studied Chinese sector. What do you say about this?

2) Congestion labeling process using K-means++ seems somewhat problematic. You treat clusters as objective congestion levels, but clustering itself is still highly dependent on feature selection and normalization choices. You should discuss more explicitly whether the resulting labels were reviewed or validated by experienced air traffic controllers.

3) I also found the explanation of the congestion threshold selection relatively weak. The binary hotspot definition relies on a threshold theta, but the rationale for choosing that threshold is not really explained in operational terms.

4) Hybrid architecture seems technically sophisticated, however, there is surprisingly little discussion about computational complexity.

5) I think the omission of the vertical dimension deserves more critical discussion. You argue that horizontal interactions dominate congestion formation, but altitude conflicts, climb/descent interactions, and flight-level imbalances can still play major operational roles in en-route complexity.

6) You compare the proposed model with several approaches, but it remains unclear whether the performance gain truly justifies the added architectural complexity.

7) Adjacency matrix construction based purely on flight flow transitions is interesting, but it may oversimplify operational relationships. Because, neighboring grids can still influence each other through controller workload balancing or rerouting constraints even without direct trajectory transitions. Right?

8) Also, expand the related work with “10.1016/j.engappai.2025.111775” by Bakirci, which presents a comprehensive evaluation of DL models under real-time operational constraints by analyzing inference latency, computational efficiency, and deployment feasibility on resource-constrained edge platforms. Including this work would strengthen the discussion on the practical deployment and operational scalability of AI-driven congestion prediction systems in real-world air traffic management environments.

9) You should also provide more discussion regarding data imbalance. Severe congestion events are naturally much rarer than normal operations, yet the manuscript does not deeply analyze how class imbalance affects training stability and prediction reliability.

10) There are no confidence intervals, repeated-run statistics, or robustness evaluations under noisy operational conditions. That makes it difficult to judge how stable the framework actually is.

11) You frequently refer to real-time prediction, but the actual inference latency and deployment feasibility are not analyzed in sufficient detail.

12) You also claim that the framework supports aviation sustainability and carbon reduction, but this part seems more speculative than experimentally demonstrated. There is no direct emission analysis or quantitative fuel-saving evaluation included in the experiments.

**********

what does this mean?). If published, this will include your full peer review and any attached files.

If you choose “no”, your identity will remain anonymous but your review may still be made public.

**Do you want your identity to be public for this peer review?**  For information about this choice, including consent withdrawal, please see our Privacy Policy

Reviewer #1: No

Reviewer #2: No

Reviewer #3: No

**********

[NOTE: If reviewer comments were submitted as an attachment file, they will be attached to this email and accessible via the submission site. Please log into your account, locate the manuscript record, and check for the action link "View Attachments". If this link does not appear, there are no attachment files.]

To ensure your figures meet our technical requirements, please review our figure guidelines: https://journals.plos.org/plosone/s/figures

You may also use PLOS’s free figure tool, NAAS, to help you prepare publication quality figures: https://journals.plos.org/plosone/s/figures#loc-tools-for-figure-preparation.

NAAS will assess whether your figures meet our technical requirements by comparing each figure against our figure specifications.

# Author comment: Author response to Decision Letter 1

**DOI:** 10.1371/journal.pone.0355927.r002

5 Jul 2026

Journal Requirement 1

Comment: Please ensure that the manuscript meets PLOS ONE style requirements, including file naming.

Response: We appreciate this instruction and treated it as part of the resubmission preparation rather than as a simple file-management matter. The submission package has been reorganized into clearly separated components, including the clean manuscript, the marked-up manuscript, the response letter, figure files, reproducibility materials, and interface demonstration materials. During this process, we checked table and figure callouts, corrected reference-list numbering, and made the manuscript files consistent with the PLOS ONE formatting expectations so that the editorial office can verify the revised materials more easily.

Journal Requirement 2

Comment: Please ensure that the corresponding author has a verified ORCID iD in Editorial Manager.

Response: We have noted and followed this requirement. The corresponding author ORCID iD is indicated as verified in Editorial Manager, and the coauthors will be encouraged to complete ORCID verification before final acceptance if needed.

Journal Requirement 3

Comment: Please follow PLOS ONE guidelines for sharing author-generated code.

Response: We agree that the code-sharing statement needed to be more explicit. The revision is based on a multi-stage computational workflow, including feature preparation, K-means++ labeling, graph construction, model training, baseline comparison, robustness testing, SHAP/SHEP attribution, and interface visualization. We have deposited the author-generated code and the corresponding de-identified grid-level data needed to reproduce the reported results on Figshare with DOI: https://doi.org/10.6084/m9.figshare.32901059. The package is organized so that readers can validate the K-means++ labels, reproduce model training from the raw15 grid-level dataset, and run the trained-model interface case without relying on private scripts.

Journal Requirement 4

Comment: Funding information should not appear in the manuscript text.

Response: We have followed this instruction and removed funding information from the manuscript text. The updated funding statement will be provided through the online submission system and, where requested, in the resubmission letter rather than in the scientific text. The revised funding statement is: This work was supported by the Graduate Student Scientific Research Innovation Fund of Civil Aviation Flight University of China (26CAFUC10019), and the Science and Technology Projects of Xizang Autonomous Region, China (XZ202601ZY0091 and XZ202403ZY0014). The funders had no role in study design, data collection and analysis, decision to publish, or preparation of the manuscript. This keeps the manuscript consistent with PLOS ONE requirements while ensuring that the submitted funding metadata are complete and accurate.

Journal Requirements 5-7

Comment: Please revise the data availability approach and deposit the minimal anonymized dataset necessary to replicate the findings in a stable public repository.

Response: We agree that reproducibility should not depend on data availability upon request. We have therefore revised the data-sharing plan and deposited the corresponding de-identified grid-level data used in the experiments, together with all author-generated code required for reproduction, on Figshare with DOI: https://doi.org/10.6084/m9.figshare.32901059. The public package includes the processed full-year feature table, K-means++ labels and validation files, graph/edge information, train/validation/test splits, configuration files, evaluation scripts, baseline scripts, robustness-test scripts, source data for reported figures, and the interface case input used for the operational visualization. With these materials, readers can independently rebuild the label generation, model training, evaluation, and visualization pipeline from the de-identified data released for this study.

Journal Requirement 8

Comment: Please provide copies of the images for all figures included in the manuscript.

Response: We prepared the figures as separate upload-ready files while retaining figure captions and callouts in the manuscript. The figure package was checked against the revised text so that the production office can match each uploaded image with the corresponding figure number, caption, and discussion in the manuscript.

Journal Requirement 9

Comment: Please ensure that Table 1 and Table 2 are referred to in the manuscript text.

Response: We checked the table sequence and agree that the manuscript needed clearer table callouts. The revised text now explicitly cites Table 1 when introducing the indicator system, Table 2 when reporting the K-means++ clustering results, Table 3 when comparing the proposed model with LSTM, DCRNN, and Graph WaveNet, and Table 4 when discussing repeated-run and perturbation robustness. This makes the flow from indicator construction, to pseudo-label validation, to model comparison, and finally to robustness testing easier to follow.

Journal Requirement 10

Comment: Please include captions for Supporting Information files and update in-text citations accordingly.

Response: We have revised the manuscript to address this requirement. The figure files in the upload package have been renamed according to the PLOS ONE Supporting Information naming convention, from S1_Fig.tif to S20_Fig.tif. The main manuscript text retains the standard main-text figure callouts and captions in the form Fig 1 through Fig 20, while a Supporting information section has been added at the end of the manuscript with matching S1 Fig through S20 Fig captions.

Journal Requirement 11

Comment: Please evaluate reviewer-recommended references.

Response: We reviewed the recommended references and incorporated those that help position the study more clearly. The added literature now distinguishes three roles: direct spatiotemporal forecasting baselines, methodological inspiration from multi-task and graph-based learning, and broader intelligent-transportation perception work that motivates congestion warning as a traffic-perception problem. We did not present all cross-domain papers as direct ATM baselines, because doing so would overstate their comparability. Instead, the revised related-work section uses them where they clarify the methodological or conceptual background of this study.

Response to Reviewer #1

We thank Reviewer #1 for the positive assessment of the manuscript structure and application value, and for the constructive suggestions on model positioning, labeling reliability, baseline comparison, graph sparsity, and related work.

Reviewer #1, Comment 1

Comment: The stacked GNN-LSTM-Transformer architecture is common in spatiotemporal prediction. Please explain why this specific stacking is suitable for ATM and compare it with end-to-end models such as ST-GCN, ASTGCN, and Informer.

Response: We agree that the original manuscript did not sufficiently explain why this architecture is appropriate for en-route ATM or how it differs from existing end-to-end spatiotemporal models. We revised the Introduction, Section 2.4, and Section 4 to make the motivation explicit. In ATM sector congestion, a warning is not generated only by local traffic volume: congestion can build over several 5-minute windows, spread along route-contiguous and neighboring grids, and appear differently depending on upstream/downstream operating states. For this reason, the GNN component is used to encode grid adjacency, route continuity, and congestion propagation; the BiLSTM component captures the buildup, persistence, and dissipation of operating states over historical windows; and cross-attention fuses spatial propagation representations with temporal operating representations before the 15-minute-ahead prediction. We also added a comparison with ST-GCN, ASTGCN, Informer-type models, DCRNN, and Graph WaveNet. ST-GCN and ASTGCN provide useful references for structured spatiotemporal graphs, but their common settings are usually designed for denser and more regular road-sensor networks, while ATM grids may be sparse, temporarily disconnected, and affected by route-contiguity constraints. Informer is effective for long-sequence temporal modeling, but it does not by itself represent sector-grid topology or upstream/downstream propagation. DCRNN and Graph WaveNet are therefore included as graph-temporal baselines in Table 3, while the proposed architecture is presented as an ATM-adapted design that combines observable grid operation, spatial propagation, and short-term temporal evolution under the raw15 feature setting.

Reviewer #1, Comment 2

Comment: K-means++ labels may cause the supervised model to learn the clustering logic rather than true operational boundaries. Please introduce controller validation or discuss the limitation.

Response: This concern is well taken. We revised the manuscript to make clear that the K-means++ labels are operationally interpreted pseudo-labels rather than manually observed ground truth. To make the labeling process more transparent, the revised version reports the observable feature set used for clustering, the candidate-K validation logic, internal validity indicators, cluster-center interpretation, and the final selection of three operating states. We also added a small expert plausibility check: representative conflict/congestion candidate windows and crowded transition windows were reviewed with personnel familiar with Guangdong and Chongqing regional control contexts. The experts did not treat the labels as absolute truth, but they considered the selected congestion and crowded cases operationally plausible when combined with traffic load, dwell time, maneuvering frequency, and delay evidence. This revision reduces the risk that readers interpret the pseudo-labels as direct manual annotations. These clarifications were added mainly in Section 3.4 and Section 5.3, with the remaining uncertainty discussed in Section 6.

Reviewer #1, Comment 3

Comment: The comparison only includes Linear Regression and LSTM. Please compare with mainstream spatiotemporal graph neural network baselines such as DCRNN and Graph WaveNet.

Response: We agree that the earlier comparison was too narrow. The revised manuscript adds representative temporal and graph-temporal baselines under the same raw15 visible-feature setting and the same 15-minute prediction horizon. Specifically, Table 3 now includes LSTM, DCRNN, Graph WaveNet, and the proposed GNN-BiLSTM-Cross-Attention model. The comparison therefore tests whether the proposed combination of graph propagation, bidirectional temporal encoding, and cross-attention improves rare T2 hotspot recognition beyond sequence-only and graph-temporal alternatives. The new table also reports L2-focused metrics rather than relying only on overall performance, which is important because severe congestion is the rare and operationally critical class.

Reviewer #1, Comment 4

Comment: The flight-flow graph may be extremely sparse at a 5-minute resolution, producing isolated nodes. Please explain how isolated nodes are handled and discuss the impact of graph sparsity.

Response: We agree that 5-minute flight-flow graphs may contain weakly connected or temporarily isolated grids. The revised method description now explains how these cases are handled. Self-loops preserve each grid's own state, static neighborhood and route-contiguity edges retain physically meaningful context, and dynamic flow edges add time-varying transfer information when flights move between grids. When a grid has few or no traversing flights in a short window, its node-level operating features and static context are still available to the model. This makes the graph formulation less dependent on a dense flow edge set at every time step. This explanation was added to the graph construction description in Section 4.2.

Reviewer #1, Comment 5

Comment: Several recent spatiotemporal traffic congestion prediction references are missing.

Response: We updated the literature discussion to include the recent spatiotemporal traffic-event and graph-prediction studies suggested by the reviewer. These references are used to clarify how related fields model rare traffic events, tipping points, and graph-based temporal propagation, while the revised text also explains why ATM sector congestion differs from road-traffic event prediction. This helps position the proposed framework as an ATM-specific adaptation rather than a direct transfer of a road-network method. The related discussion and references were added in Section 2.3 and the reference list.

Response to Reviewer #2

We thank Reviewer #2 for the constructive comments regarding technical contribution, clustering transparency, related work, supervised cause-weight ground truth, reproducibility, and deployment conditions.

Reviewer #2, Q1

Comment: The manuscript combines existing techniques. Please better distinguish the technical contribution from existing spatiotemporal prediction models.

Response: We agree that the contribution needed to be stated as more than a combination of known components. The revision now frames the contribution around mechanism alignment and reproducibility. K-means++ converts observable operational indicators into three interpretable pseudo-label states; the GNN represents spatial and route-related propagation; the BiLSTM captures temporal evolution over historical 5-minute windows; cross-attention fuses spatial and temporal representations; and SHAP/SHEP attribution explains model warnings after inference rather than adding an unverifiable supervised cause-regression target. This reframing makes the contribution clearer: the paper is not claiming novelty from each module in isolation, but from an ATM-oriented pipeline that connects label construction, spatiotemporal prediction, and post-hoc operational explanation. This reframing is reflected in the contribution statements in the Introduction and in the method description in Sections 3.4 and 4.

Reviewer #2, Q2

Comment: Please provide more details on K-means++ cluster number selection, cluster center interpretation, silhouette coefficient, elbow analysis, and expert validation.

Response: We agree that the clustering procedure needed to be more transparent. The revised manuscript now reports the observable clustering variables, normalization, candidate-K comparison, internal validity metrics, cluster-center interpretation, and the expert plausibility check. We also clarified that K=3 is supported by the data-driven validation and the operational interpretation of free-flow, crowded transition, and congested states, rather than being imposed after the fact to match a desired label count. This is important because the downstream model is trained on these pseudo-labels, so readers need to understand both the statistical and operational basis of the labeling process. These details are now reported in Section 3.4, Section 5.3, Figure 13, Figure 14, and Table 2.

Reviewer #2, Q3

Comment: Please expand the related work with additional intelligent transportation, visual perception, and multi-task learning studies.

Response: We considered the recommended cross-domain works and incorporated them where they help the paper's logic. The dual-pathway driver-emotion study is used as a methodological reference for combining shared and task-specific representations; the M-GBT short-term load-forecasting work helps motivate regional graph coupling and Transformer-style temporal modeling; and visual perception studies are discussed as conceptual support for treating congestion prediction as an intelligent transportation perception problem. At the same time, the revision avoids presenting these studies as direct ATM baselines, because their data, operational constraints, and evaluation targets differ from sector congestion warning. These references and distinctions were added to Sections 2.3 and 2.4.

Reviewer #2, Q4

Comment: The ground truth for the cause factor weight regression task is unclear.

Response: We agree that the original cause-weight regression description could be misunderstood as relying on unavailable ground-truth cause weights. We therefore made a substantive methodological revision. The supervised training objective now focuses on future congestion-level prediction and T2 hotspot warning, both using information available before the prediction time. The previous supervised cause-regression head and its multi-task loss were removed. Causal explanation is now handled after inference through SHAP/SHEP-style grouped attribution: the first-level chart separates external triggering risk from congestion-propagation risk, and the second-level chart decomposes the external component into weather, restricted-area, controller-workload, and wake-intensity contributions. This change better matches the available data, avoids unverifiable cause labels, and keeps explanation separate from the predictive training target. The corresponding method revision is described in Section 4.4, and the revised attribution presentation is used in Figure 5, Figure 19, and Figure 20.

Reviewer #2, Q5

Comment: The data availability statement indicates that some processed data and scripts are available only upon reasonable request. Please provide reproducibility materials such as anonymized data, feature matrices, adjacency matrices, code, parameter settings, and preprocessing scripts.

Response: We agree that the data and code availability statement needed to be stronger and more specific. The corresponding de-identified grid-level dataset and all author-generated code needed to reproduce the study have been deposited on Figshare with DOI: https://doi.org/10.6084/m9.figshare.32901059. The package includes feature matrices, K-means++ validation data and model files, graph construction inputs, training/validation/test splits, model training and evaluation scripts, baseline scripts, robustness-test scripts, figure source data, and the interface replay input. These released materials are designed to let readers reproduce the reported labels, model results, and visual case study using the same de-identified data basis as the revised manuscript. The availability information will be provided through the online submission system and the Figshare repository record rather than as a separate funding or data statement in the manuscript text.

Reviewer #2, Q6

Comment: The manuscript claims real-time application and decision-support value but does not sufficiently analyze practical deployment conditions.

Response: We agree that a real-time or operational-decision claim should be supported by deployment information, not only accuracy metrics. The revised manuscript now reports the dominant complexity terms for graph propagation, recurrent temporal encoding, and attention fusion, along with parameter count and empirical inference-time estimates. We also clarify that the system is intended for minute-level tactical warning within a 5-minute data update cycle, not for millisecond-level aircraft control. This makes the operational scope more precise and prevents the accuracy results from being interpreted as a complete deployment evaluation. The complexity and deployment discussion was added in Section 5.4.

Response to Reviewer #3

We thank Reviewer #3 for the detailed and helpful comments on generalization, pseudo-labeling, threshold interpretation, computational cost, vertical dimensions, adjacency construction, robustness, deployment, and sustainability claims.

Reviewer #3, Comment 1

Comment: The validation is limited to a single sector and one year of data. Please discuss generalization to other airspace structures, traffic densities, and control procedures.

Response: We agree that the empirical scope should be described carefully. In the revision, we distinguish between the generality of the method and the sector-specific nature of the trained model. The proposed framework is based on general data-driven and machine-learning procedures, including grid-level feature construction, K-means++ operating-state labeling, graph-temporal prediction, and post-hoc SHAP/SHEP attribution; these procedures are not limited to ZGGGAR15 in principle. However, the trained parameters, graph structure, normalization range, clustering centers, and warning threshold are learned from the Chinese ZGGGAR15 sector data used in this study. Therefore, when the framework is applied to another sector with different airspace geometry, traffic density, route layout, weather exposure, or control procedures, the local grid definition and graph should be rebuilt and the model should be recalibrated or retrained with the corresponding sector data. Section 6 now states this distinction more explicitly: the method is transferable, whereas the current trained model and numerical settings are sector-specific.

Reviewer #3, Comment 2

Comment: K-means++ labels depend on feature selection and normalization. Please discuss whether the labels were reviewed by experienced controllers.

Response: We agree that K-means++ labels are sensitive to feature choice and normalization. The revised manuscript now states the clustering feature set explicitly and explains that external factors such as weather, restricted areas, controller workload, and wake intensity are not used to define the clusters. This separation is important because the clusters are intended to reflect internal operating-state congestion, while external variables are used later as predictors and attribution factors. We also added the expert plausibility check and a limitation noting that larger controller-labeled datasets would further strengthen future validation. These clarifications appear in Section 3.4, Section 5.3, and Section 6.

Reviewer #3, Comment 3

Comment: The operational explanation of the binary hotspot threshold theta is weak.

Response: We agree that the operational meaning of the binary hotspot threshold theta needed to be stated more plainly. The model first outputs a probability that a grid-time unit will become a T2 hotspot within the 15-minute prediction horizon. The threshold theta is the alarm line used to convert this probability into an operational warning: when the predicted T2 probability is greater than or equal to theta, the grid is marked as a hotspot; otherwise it is not. In the revised manuscript, theta is selected on the validation segment to balance missed severe-congestion warnings and false alarms, and then it is fixed before testing. Thus, theta should be read as a warning-sensitivity setting rather than a new physical congestion index: a lower theta would issue more alerts earlier, while a higher theta would be more conservative. The manuscript now explains this rule in Section 3.4 and Section 5.4, with the fixed operating point illustrated in Figure 18.

Reviewer #3, Comment 4

Comment: The hybrid architecture is complex, but computational complexity is insufficiently discussed.

Response: We agree that architecture complexity must be discussed together with computational feasibility. The revised manuscript reports the main costs of graph propagation, recurrent temporal encoding, and attention fusion, and relates them to the 5-minute update cycle used in the study. The model has approximately 0.18 million trainable parameters, with forward inference around 0.09 seconds on GPU and 0.68 seconds on CPU in the reported setting; the rolling update workflow is estimated at 20-45 seconds. These values do not replace a field deployment test, but they show that the method is compatible with the tactical warning interval considered in the paper. The computational-cost discussion is included in Section 5.4.

Reviewer #3, Comment 5

Comment: The missing vertical dimension requires more critical discussion.

Response: We agree that the two-dimensional grid abstraction requires a more careful discussion. The revised manuscript explains that the model primarily represents horizontal grid interactions, while vertical operational complexity is partly reflected through altitude-change frequency and related maneuver indicators. We also clarified that this is a simplification: a full three-dimensional airspace graph could better represent altitude-layer interactions, vertical separation constraints, and climbing/descending conflicts. This limitation is now stated explicitly as an important direction for future work. This clarification appears in Section 3.2, Section 5.1, and Section 6.

Reviewer #3, Comment 6

Comment: It is unclear whether the performance gain is worth the additional architecture complexity.

Response: We agree that performance improvement alone is not enough to justify a more complex model. The revised manuscript now connects complexity to operational value by comparing the proposed framework with LSTM, DCRNN, and Graph WaveNet under the same raw15 input setting. The comparison focuses on rare T2 hotspot precision, recall, F1-score, and average precision, because those metrics reflect the warning task more directly than overall accuracy. The accompanying computational-cost discussion then shows that the additional graph and attention components remain feasible within the intended 5-minute update cycle. The model comparison is reported in Section 5.5 and Table 3, with computational feasibility discussed in Section 5.4.

Reviewer #3, Comment 7

Comment: The adjacency matrix based only on flight-flow transfer may oversimplify operational relationships such as workload balancing or rerouting constraints.

Response: We agree that direct flight-flow transfer alone cannot represent all operational relationships. The revised graph description therefore includes static neighboring-grid context and route-contiguity information in addition to dynamic flow edges. The limitations section also acknowledges that some operational relationships, such as workload balancing, rerouting constraints, common weather exposure, and restricted-area effects, may be better represented by a future dynamic multi-relation graph. This clarifies what the current graph captures and what remains outside its scope. The graph construction was revised in Section 4.2, and the remaining limitation is discussed in Section 6.

Reviewer #3, Comment 8

Comment: Please cite Bakirci (10.1016/j.engappai.2025.111775) to strengthen the deployment discussion.

Response: We added the suggested deployment-related reference and used it to strengthen the discussion of accuracy-latency tradeoffs and resource-aware AI deployment. We cite it as a deployment and resource-efficiency reference rather than as a direct ATM congestion baseline. This keeps the related-work comparison accurate while still using the reference to support the practical deployment discussion. The added reference is discussed in Sections 2.3-2.4 and linked to deployment considerations in Section 5.4.

Reviewer #3, Comment 9

Comment: Please discuss data imbalance, because severe congestion events are much rarer than normal operation.

Response: We agree that class imbalance is central to the problem, because severe congestion is much rarer than normal operation. The revised manuscript therefore emphasizes L2 precision, recall, F1-score, and average precision rather than relying on overall accuracy. We also describe the use of balanced temporal sampling and hotspot-oriented loss weighting so that training does not become dominated by free-flow windows. This makes the evaluation more consistent with the operational objective: detecting rare but consequential congestion warnings. The imbalance handling and L2-focused metrics are described in Section 5.4 and reported in Figure 18 and Section 5.5.

Reviewer #3, Comment 10

Comment: There are no confidence intervals, repeated-run statistics, or robustness evaluation under noisy conditions.

Response: We agree that a single run is insufficient evidence for robustness. The revised manuscript adds five-seed repeated training and controlled perturbation tests, including Gaussian feature noise, random feature masking, and time-window dropout. The results show that the proposed model remains stable under moderate input disturbance, while also revealing that temporal-window dropout has the largest effect, which is consistent with the role of short-term congestion evolution in the model. This gives readers a clearer view of both stability and sensitivity. These robustness results are reported in Section 5.5 and Table 4.

Reviewer #3, Comment 11

Comment: Real-time prediction is mentioned, but actual inference latency and deployment feasibility are not analyzed.

Response: We agree that the meaning of real-time prediction should be made concrete. The revised manuscript now reports model size, GPU/CPU inference time, rolling update time, and the intended 5-minute data update cycle. It also clarifies that the May 13, 2023 09:15 replay case uses the already trained model for 15-minute-ahead prediction and visualization, without retraining or parameter adjustment for that case. This helps distinguish model evaluation, operational replay, and visualization functions. The real-time interpretation is clarified in Section 5.4, while the replay case is described with Figure 19 and Figure 20 in Section 5.5.

Reviewer #3, Comment 12

Comment: The sustainability and carbon-reduction claims appear speculative because no direct emissions or fuel-saving analysis is included.

Response: We agree that the sustainability discussion should not imply a direct emissions result. We revised the manuscript to state that the present work addresses congestion prediction under the FF-ICE operational concept and can provide indirect support for future fuel-use and emissions analysis. Direct carbon-emission quantification is now treated as outside the scope of this study and as future work requiring dedicated fuel-burn and emissions models. This revision avoids overstating the environmental conclusion while still acknowledging the operational relevance of earlier congestion warning. The wording was adjusted in the Abstract, Conclusion, and Section 6.

Attachment Submitted filename: Response to Reviewers.docx

# Peer review: Decision Letter 1

**DOI:** 10.1371/journal.pone.0355927.r003

**Authors:** Brian Patrick Weaver

27 Aug 2026

Dear Dr. YAN,

Thank you for submitting your manuscript to PLOS One. After careful consideration, we feel that it has merit but does not fully meet PLOS One’s publication criteria as it currently stands. Therefore, we invite you to submit a revised version of the manuscript that addresses the points raised during the review process.

The manuscript has been evaluated by two new reviewers, and their comments are available below. The reviewers have raised a number of concerns with your manuscript that need attention. Could you please revise the manuscript to carefully address the concerns raised?

Please submit your revised manuscript by Oct 11 2026 11:59PM. If you will need more time than this to complete your revisions, please reply to this message or contact the journal office at plosone@plos.org. When you're ready to submit your revision, log on to https://www.editorialmanager.com/pone/ and select the 'Submissions Needing Revision' folder to locate your manuscript file.

- A letter that responds to each point raised by the academic editor and reviewer(s). You should upload this letter as a separate file labeled 'Response to Reviewers'.
- A marked-up copy of your manuscript that highlights changes made to the original version. You should upload this as a separate file labeled 'Revised Manuscript with Track Changes'.
- An unmarked version of your revised paper without tracked changes. You should upload this as a separate file labeled 'Manuscript'.

If you would like to make changes to your financial disclosure, please include your updated statement in your cover letter. Guidelines for resubmitting your figure files are available below the reviewer comments at the end of this letter.

If applicable, we recommend that you deposit your laboratory protocols in protocols.io to enhance the reproducibility of your results. Protocols.io assigns your protocol its own identifier (DOI) so that it can be cited independently in the future. For instructions see: https://journals.plos.org/plosone/s/submission-guidelines#loc-laboratory-protocols. Additionally, PLOS One offers an option for publishing peer-reviewed Lab Protocol articles, which describe protocols hosted on protocols.io. Read more information on sharing protocols at https://plos.org/protocols?utm_medium=editorial-email&utm_source=authorletters&utm_campaign=protocols.

As the corresponding author, your ORCID iD is verified in the submission system and will appear in the published article. PLOS supports the use of ORCID, and we encourage all coauthors to register for an ORCID iD and use it as well. Please encourage your coauthors to verify their ORCID iD within the submission system before final acceptance, as unverified ORCID iDs will not appear in the published article. *Only*  the individual author can complete the verification step; PLOS staff *cannot*  verify ORCID iDs on behalf of authors.

We look forward to receiving your revised manuscript.

Kind regards,

Brian Patrick Weaver, Ph.D.

Staff Editor

PLOS One

Journal Requirements:

If the reviewer comments include a recommendation to cite specific previously published works, please review and evaluate these publications to determine whether they are relevant and should be cited. There is no requirement to cite these works unless the editor has indicated otherwise.

[Note: HTML markup is below. Please do not edit.]

Reviewers' comments:

Reviewer's Responses to Questions

**Comments to the Author**

Reviewer #2: All comments have been addressed

Reviewer #3: All comments have been addressed

Reviewer #4: (No Response)

Reviewer #5: (No Response)

**********

2. Is the manuscript technically sound, and do the data support the conclusions?

Reviewer #2: Yes

Reviewer #3: Yes

Reviewer #4: Yes

Reviewer #5: Partly

**********

3. Has the statistical analysis been performed appropriately and rigorously?

Reviewer #2: Yes

Reviewer #3: Yes

Reviewer #4: Yes

Reviewer #5: Yes

**********

4. Have the authors made all data underlying the findings in their manuscript fully available?

The PLOS Data policy

Reviewer #2: Yes

Reviewer #3: Yes

Reviewer #4: Yes

Reviewer #5: Yes

**********

5. Is the manuscript presented in an intelligible fashion and written in standard English?

Reviewer #2: Yes

Reviewer #3: Yes

Reviewer #4: Yes

Reviewer #5: Yes

**********

Reviewer #2: All the questions have been addressed well. I thank the authors for the prompt responses. I thus recommend it for publication as is.

Reviewer #3: Good job on the revision. I have no further comments. Good job on the revision. I have no further comments.

Reviewer #4: My commendations to the authors for the comprehensive revisions and the good work. Overall, I think the article is technically sound and provides a reasonable contribution to the ATM domain. My recommendation is 'accept with minor revisions'.

Since this is my first time reviewing the manuscript, I have added some suggestions for the authors' consideration. I have tried to provide suggestions for primarily superficial revisions, as I can see that there have already been extensive technical revisions from the first round of review. My apologies if the comments here result in an extended manuscript processing time.

-- Technical Comments --

1. In Section 3, it is not clear how the following variables in Table 1 are derived: i) Congestion level, ii) Label confidence, iii) Weather intensity, iv) Restricted-area impact, v) Controller workload, and vi) Wake intensity.

For the above variables, further details should be provided on the type of data used (e.g., track data, SIGMET, NOTAM, etc) and high-level data processing. Non-standard variables (e.g., controller workload) may need more explanation.

2. In Section 4.2, please provide a short elaboration on the aggregation interval and if a_ij is a temporal variable (e.g., a_ijt)

3. For Eq (10), the 't' in superscript H^(t.l) is referring to the timestep dimension?

4. In Figure 6.A., please double-check the use of adjacency matrix A. My understanding is that the adjacency matrix is constant through all grid-points and layers, so I do not understand the use of A_(n-1)^(t,l-1)

5. For Eq (39), there is insufficient detail on the loss components. It will be good to expand L_cls and L_hotspot.

6. For Fig 16, the scatter points on this plot looks strange, but without more information on how the variables 'Control load' and 'Traffic flow' are derived, I am not able to comment further.

-- Non-Technical Comments --

7. It is important to qualify why the 15-minute lookahead prediction horizon was used (e.g., line 80, or in the conclusion), especially if there are any particular operational significance or use cases. In my opinion, 15 min is far too short a horizon for effective ATFM or ENR mitigation measures (e.g., speed adjustments or rerouting). With 15 minute advance warning, ATCOs will usually need to resort to more workload intensive measures such as vectoring or holding.

8. FF-ICE and trajectory sharing are mentioned in the introduction. It may be worthwhile to consider whether shared trajectories (e.g., via FF-ICE/R2) in future trajectory-based operations are sufficiently accurate such that there is no need for flow-based forecasting models such as the one presented in the paper, or if such models can be augmented with the new trajectory information.

9. For the literature review in Section 2, it is noted that a great deal of references are primarily from Chinese researchers. I would suggest to also include some of the more significant ATM works from US or Europe for completeness and also for visibility of the paper (e.g., concepts related to airspace permeability, ATFM hotspot prediction, or weather-impact forecast).

10. Some terms require further clarification

10.i. Section 3.1:

- P_i(X_i, Y_i): is P_i the polygon, and X_i and Y_i the set of polygon coordinates?

- H_max, H_1max: it is not clear what is the difference between H_max and H_1max

- Position (lambda, phi, h): I assume these are lat, lon, alt, but it is not explicitly stated

- Velocity (v, alpha): it is not clear what are these variables

- Type (P): it is not clear what is this variable (wake turbulence category?)

10.ii. Section 3.2:

- For Fig 2, I believe that K_1 and K_2 are mislabelled in the figure vs in the text (MET impacts are labelled as K_2 in the figure, and restricted airspace as K_1).

11. For Fig 9, the altitude labels are in metres. I suggest to use 'ft' or 'FL', where FL is in 100s of ft, as this is the more conventional representation?

12. For Fig 10, to clarify, the x-axis on the chart = month of year, and y-axis = traffic volume through ZGGG AR15?

13. For Fig 12, it is not clear what the flight levels represent for the air routes in the figure.

14. For Table 2, please provide units of the various variables under the column 'Label Code'.

15. In Section 5.4, some parts are repeated from Section 4. To help with the conciseness of the article, it is suggested to refer the reader back to Section 4 instead of repeating the content here.

16. Some acronyms have not been expanded (e.g., FF-ICE, T0 / T1 / T2 hotspot, SHAP/SHEP, GAT)

Reviewer #5: General Summary

Overall, the manuscript is within the scope of PLOS ONE and provides a technically coherent and reproducible application of spatiotemporal learning to sector-congestion warning. I recommend Revision. The main points that still require attention concern the transparency of the temporal evaluation protocol and the interpretation of the very high short-horizon predictive performance. In particular, the manuscript should make the train/validation/test separation fully explicit and provide a simple check showing that the reported 15-minute-ahead performance is not driven predominantly by persistence of the current congestion state. In addition, a small moderation of the strongest operational claims would better reflect the present single-sector retrospective validation. These are focused revisions and do not require a redesign of the study.

Major Comments

• Temporal split and leakage control. The manuscript refers to a chronological test segment and states that the warning threshold is selected on the validation segment, but the exact temporal boundaries of the training, validation, and test partitions are not sufficiently explicit. Please report the corresponding date ranges (or an equivalently clear chronological definition) and state explicitly that all data-dependent preprocessing and model-selection steps relevant to the reported test performance are determined without using test-period information. This clarification is important because the model is built from rolling historical windows and the reported accuracy is very high.

• Distinguishing forecasting skill from short-term state persistence. The prediction horizon is 15 minutes, whereas the model uses a relatively long recent history and congestion states may remain stable over consecutive 5-minute windows. To make the predictive result easier to interpret, please add one simple persistence/last-state reference or, alternatively, report performance separately for transitions into T2 (e.g., T0/T1 to T2) and persistence of an already congested state (T2 to T2). A compact analysis of this type would provide direct evidence that the framework contributes to early warning rather than mainly reproducing short-term state continuity.

• Scope of the operational claim. The retrospective evaluation is based on one sector and one year of data, which is appropriate for a proof-of-concept study, but the Abstract currently describes the framework as providing “operationally usable” support. Please moderate this wording to emphasize potential operational applicability or decision-support value, and state clearly that validation across additional sectors and operational environments is still required before broader deployment claims can be made. No additional external dataset is essential for the present manuscript if the scope of the conclusions is stated proportionately.

Minor Comments

• Please standardize the terminology “spatiotemporal” versus “spatio-temporal” throughout the manuscript. Both forms are currently used; adopting one form consistently (preferably “spatiotemporal,” consistent with the title) would improve editorial consistency.

• In Eq. (16), the output dimension is written as d_out, whereas the following sentence defines F_out as the output feature dimension. Please use a single symbol consistently and define it unambiguously.

• Table 2: Please report the units of measurement for the variables and thresholds presented in Table 2 (e.g., average grid dwell time and intervention frequencies). If any quantities are normalized or dimensionless, this should be stated explicitly to ensure unambiguous interpretation of the clustering boundaries.

**********

what does this mean?). If published, this will include your full peer review and any attached files.

If you choose “no”, your identity will remain anonymous but your review may still be made public.

**Do you want your identity to be public for this peer review?**  For information about this choice, including consent withdrawal, please see our Privacy Policy

Reviewer #2: No

Reviewer #3: No

Reviewer #4: **Yes:**  Yixiang Lim

Reviewer #5: No

**********

[NOTE: If reviewer comments were submitted as an attachment file, they will be attached to this email and accessible via the submission site. Please log into your account, locate the manuscript record, and check for the action link "View Attachments". If this link does not appear, there are no attachment files.]

To ensure your figures meet our technical requirements, please review our figure guidelines: https://journals.plos.org/plosone/s/figures

You may also use PLOS’s free figure tool, NAAS, to help you prepare publication quality figures: https://journals.plos.org/plosone/s/figures#loc-tools-for-figure-preparation.

NAAS will assess whether your figures meet our technical requirements by comparing each figure against our figure specifications.

Attachment Submitted filename: Reviewer_Report_PONE-D-26-08794R1.pdf

# Author comment: Author response to Decision Letter 2

**DOI:** 10.1371/journal.pone.0355927.r004

15 Sep 2026

We sincerely thank the Editor and the reviewers for their careful evaluation and constructive comments. We have revised the manuscript in accordance with all comments, including clarification of the data processing procedure, model methodology, chronological data splitting, state-transition analysis, baseline comparison, and the presentation of figures and tables. Detailed point-by-point responses and all corresponding revisions are provided in the uploaded “Response to Reviewers” file.

Attachment Submitted filename: Response_to_Reviewers_auresp_2.docx

# Peer review: Decision Letter 2

**DOI:** 10.1371/journal.pone.0355927.r005

**Authors:** Shuangxi Liu

4 Oct 2026

Dear Dr. YAN,

Thank you for submitting your manuscript to PLOS One. After careful consideration, we feel that it has merit but does not fully meet PLOS One’s publication criteria as it currently stands. Therefore, we invite you to submit a revised version of the manuscript that addresses the points raised during the review process.

==============================

**Comments from AE:**

==============================

Please submit your revised manuscript by Nov 18 2026 11:59PM. If you will need more time than this to complete your revisions, please reply to this message or contact the journal office at plosone@plos.org. When you're ready to submit your revision, log on to https://www.editorialmanager.com/pone/ and select the 'Submissions Needing Revision' folder to locate your manuscript file.

- A letter that responds to each point raised by the academic editor and reviewer(s). You should upload this letter as a separate file labeled 'Response to Reviewers'.
- A marked-up copy of your manuscript that highlights changes made to the original version. You should upload this as a separate file labeled 'Revised Manuscript with Track Changes'.
- An unmarked version of your revised paper without tracked changes. You should upload this as a separate file labeled 'Manuscript'.

If you would like to make changes to your financial disclosure, please include your updated statement in your cover letter. Guidelines for resubmitting your figure files are available below the reviewer comments at the end of this letter.

If applicable, we recommend that you deposit your laboratory protocols in protocols.io to enhance the reproducibility of your results. Protocols.io assigns your protocol its own identifier (DOI) so that it can be cited independently in the future. For instructions see: https://journals.plos.org/plosone/s/submission-guidelines#loc-laboratory-protocols. Additionally, PLOS One offers an option for publishing peer-reviewed Lab Protocol articles, which describe protocols hosted on protocols.io. Read more information on sharing protocols at https://plos.org/protocols?utm_medium=editorial-email&utm_source=authorletters&utm_campaign=protocols.

As the corresponding author, your ORCID iD is verified in the submission system and will appear in the published article. PLOS supports the use of ORCID, and we encourage all coauthors to register for an ORCID iD and use it as well. Please encourage your coauthors to verify their ORCID iD within the submission system before final acceptance, as unverified ORCID iDs will not appear in the published article. *Only*  the individual author can complete the verification step; PLOS staff *cannot*  verify ORCID iDs on behalf of authors.

We look forward to receiving your revised manuscript.

Kind regards,

Shuangxi Liu, Ph.D.

Academic Editor

PLOS One

Journal Requirements:

If the reviewer comments include a recommendation to cite specific previously published works, please review and evaluate these publications to determine whether they are relevant and should be cited. There is no requirement to cite these works unless the editor has indicated otherwise.

Please review your reference list to ensure that it is complete and correct. If you have cited papers that have been retracted, please include the rationale for doing so in the manuscript text, or remove these references and replace them with relevant current references. Any changes to the reference list should be mentioned in the rebuttal letter that accompanies your revised manuscript. If you need to cite a retracted article, indicate the article’s retracted status in the References list and also include a citation and full reference for the retraction notice.

Additional Editor Comments:

Comments from AE:

Two reviewers recommended acceptance, while one reviewer still has some issues that require further clarification from the authors. I therefore recommend minor revision.

[Note: HTML markup is below. Please do not edit.]

Reviewers' comments:

Reviewer's Responses to Questions

**Comments to the Author**

Reviewer #2: All comments have been addressed

Reviewer #3: All comments have been addressed

Reviewer #4: (No Response)

**********

2. Is the manuscript technically sound, and do the data support the conclusions?

Reviewer #2: (No Response)

Reviewer #3: Yes

Reviewer #4: Yes

**********

3. Has the statistical analysis been performed appropriately and rigorously?

Reviewer #2: (No Response)

Reviewer #3: Yes

Reviewer #4: Yes

**********

4. Have the authors made all data underlying the findings in their manuscript fully available?

The PLOS Data policy

Reviewer #2: (No Response)

Reviewer #3: Yes

Reviewer #4: Yes

**********

5. Is the manuscript presented in an intelligible fashion and written in standard English?

Reviewer #2: (No Response)

Reviewer #3: Yes

Reviewer #4: Yes

**********

Reviewer #2: All the questions have been addressed well. I thank the authors for the prompt responses. I thus recommend it for publication as is.

Reviewer #3: Good job on the revision. I have no further comments. Good job on the revision. I have no further comments.

Reviewer #4: Thank you for the huge effort in addressing my previous comments. As I was not able to locate the changes referred to by some of the responses, I would kindly request for your assistance to point me to the right para / section, or otherwise paste the relevant changes directly in your follow-on response. I apologies for the additional review stage, and am happy to recommend for publication once the items below are satisfied.

Item #1 (Previously Comment 3)

Comment: In Eq. (10), the meaning of the superscript t in H(t,l) should be defined.

Response: Thank you for identifying this notation gap. We added a definition immediately after Eq. (10):

t denotes the time step in the historical five-minute sequence, l denotes the graph layer, and H(t,l) denotes

the node representation at time t after graph layer l. We checked Eqs. (10)-(15) and the accompanying

text so that t consistently denotes time and l consistently denotes the graph layer. This local clarification

prevents readers from confusing a time index with a layer index and improves the readability of the model

derivation.

Item #2 (Previously Comment 7)

Comment: Please explain why a 15-minute prediction horizon was selected and how it relates to air

traffic flow management actions.

Response: Thank you for encouraging us to make the operational meaning of the prediction horizon

explicit. We added an explanation in the model-training and prediction description: with five-minute

aggregation, a 15-minute horizon corresponds to three future time steps and is long enough to identify the

buildup and spatial spread of congestion while remaining relevant to short-term flow-management

coordination. It can provide time for tactical measures such as speed control, metering, rerouting

coordination, or holding preparation, without presenting the result as a replacement for longer-horizon

strategic planning. We also clarify that 30-minute and 60-minute horizons would require separately

trained and validated models. This establishes the practical rationale and scope of the selected horizon.

**********

what does this mean?). If published, this will include your full peer review and any attached files.

If you choose “no”, your identity will remain anonymous but your review may still be made public.

**Do you want your identity to be public for this peer review?**  For information about this choice, including consent withdrawal, please see our Privacy Policy

Reviewer #2: No

Reviewer #3: No

Reviewer #4: **Yes:**  Yixiang Lim

**********

[NOTE: If reviewer comments were submitted as an attachment file, they will be attached to this email and accessible via the submission site. Please log into your account, locate the manuscript record, and check for the action link "View Attachments". If this link does not appear, there are no attachment files.]

To ensure your figures meet our technical requirements, please review our figure guidelines: https://journals.plos.org/plosone/s/figures

You may also use PLOS’s free figure tool, NAAS, to help you prepare publication quality figures: https://journals.plos.org/plosone/s/figures#loc-tools-for-figure-preparation.

NAAS will assess whether your figures meet our technical requirements by comparing each figure against our figure specifications.

# Author comment: Author response to Decision Letter 3

**DOI:** 10.1371/journal.pone.0355927.r006

5 Oct 2026

We sincerely apologize for our oversight. In the previous round, we inadvertently did not upload the correct version of the manuscript, which resulted in the two points raised by Reviewer #4 not being reflected in the manuscript. We are very grateful to Reviewer #4 for pointing this out and for the careful follow-up. We have now revised the manuscript accordingly. For details, please refer to the revised manuscript and the point-by-point response.

Attachment Submitted filename: Response_to_Reviewers_auresp_3.docx

# Peer review: Decision Letter 3

**DOI:** 10.1371/journal.pone.0355927.r007

**Authors:** Shuangxi Liu

8 Oct 2026

An operational spatiotemporal prediction framework for sector congestion in air traffic management

PONE-D-26-08794R3

Dear Dr. YAN,

We’re pleased to inform you that your manuscript has been judged scientifically suitable for publication and will be formally accepted for publication once it meets all outstanding technical requirements.

Within one week, you’ll receive an e-mail detailing the required amendments. When these have been addressed, you’ll receive a formal acceptance letter and your manuscript will be scheduled for publication.

An invoice will be generated when your article is formally accepted. Please note, if your institution has a publishing partnership with PLOS and your article meets the relevant criteria, all or part of your publication costs will be covered. Please make sure your user information is up-to-date by logging into Editorial Manager at Editorial Manager® and clicking the ‘Update My Information' link at the top of the page. For questions related to billing, please contact billing support.

If your institution or institutions have a press office, please notify them about your upcoming paper to help maximize its impact. If they’ll be preparing press materials, please inform our press team as soon as possible -- no later than 48 hours after receiving the formal acceptance. Your manuscript will remain under strict press embargo until 2 pm Eastern Time on the date of publication. For more information, please contact onepress@plos.org.

Kind regards,

Shuangxi Liu, Ph.D.

Academic Editor

PLOS One

Additional Editor Comments (optional):

**Comments from AE:**

Reviewer 4 endorses the authors' revisions. The paper satisfies acceptance requirements, and I agree to its publication.

Reviewers' comments:

Reviewer's Responses to Questions

**Comments to the Author**

Reviewer #4: All comments have been addressed

**********

2. Is the manuscript technically sound, and do the data support the conclusions?

Reviewer #4: Yes

**********

3. Has the statistical analysis been performed appropriately and rigorously?

Reviewer #4: Yes

**********

4. Have the authors made all data underlying the findings in their manuscript fully available?

The PLOS Data policy

Reviewer #4: Yes

**********

5. Is the manuscript presented in an intelligible fashion and written in standard English?

Reviewer #4: Yes

**********

Reviewer #4: My thanks to the authors for the quick turnaround. No further comments and happy to recommend for publication.

Congratulations and good work!

**********

what does this mean?). If published, this will include your full peer review and any attached files.

If you choose “no”, your identity will remain anonymous but your review may still be made public.

**Do you want your identity to be public for this peer review?**  For information about this choice, including consent withdrawal, please see our Privacy Policy

Reviewer #4: **Yes:**  Yixiang Lim

**********

# Editor report: Acceptance letter

**DOI:** 10.1371/journal.pone.0355927.r008

**Authors:** Shuangxi Liu

PONE-D-26-08794R3

PLOS One

Dear Dr. Yan,

I'm pleased to inform you that your manuscript has been deemed suitable for publication in PLOS One. Congratulations! Your manuscript is now being handed over to our production team.

At this stage, our production department will prepare your paper for publication. This includes ensuring the following:

* All references, tables, and figures are properly cited

* All relevant supporting information is included in the manuscript submission,

* There are no issues that prevent the paper from being properly typeset

You will receive further instructions from the production team, including instructions on how to review your proof when it is ready. Please keep in mind that we are working through a large volume of accepted articles, so please give us a few days to review your paper and let you know the next and final steps.

Lastly, if your institution or institutions have a press office, please let them know about your upcoming paper now to help maximize its impact. If they'll be preparing press materials, please inform our press team within the next 48 hours. Your manuscript will remain under strict press embargo until 2 pm Eastern Time on the date of publication. For more information, please contact onepress@plos.org.

You will receive an invoice from PLOS for your publication fee after your manuscript has reached the completed accept phase. If you receive an email requesting payment before acceptance or for any other service, this may be a phishing scheme. Learn how to identify phishing emails and protect your accounts at https://explore.plos.org/phishing.

If we can help with anything else, please email us at customercare@plos.org.

Thank you for submitting your work to PLOS One and supporting open access.

Kind regards,

PLOS One Editorial Office Staff

on behalf of

Dr. Shuangxi Liu

Academic Editor

PLOS One
